# Public support for novel carbon removal hinges on procedural and distributive fairness

**DOI:** 10.1038/s41558-026-02741-7

**Published:** 2026-09-24

**Authors:** Livia Fritz, Lucilla Losi, Christine Merk, Michela Boldrini, Valentina Bosetti, Chad M. Baum, Benjamin K. Sovacool

**Affiliations:** 1 Department of Geography and Environment and Institute for Environmental Sciences, University of Geneva, Geneva, Switzerland; 2 Department of Business Development and Technology, School of Business and Social Sciences, Aarhus University, Herning, Denmark; 3 Kiel Institute for the World Economy, Kiel, Germany; 4 Department of Economics, Bocconi University, Milan, Italy; 5 CMCC Foundation–Euro-Mediterranean Center on Climate Change, Lecce, Italy; 6 RFF-CMCC European Institute on Economics and the Environment, Milan, Italy; 7 Department of Earth and Environment, Institute for Global Sustainability, Boston University, Boston, MA USA; 8 Bennett Institute for Innovation and Policy Acceleration, University of Sussex Business School, Brighton, UK

**Keywords:** Climate-change mitigation, Climate-change mitigation

## Abstract

Alongside the need for rapid emissions reductions, removing carbon dioxide from the atmosphere is emerging as a central pillar of global climate policy. Scaling up carbon removal raises pressing questions about how this can be done in an equitable and socially legitimate manner. Using vignette experiments with nationally representative surveys in six global south and global north countries (*N* = 10,852 respondents across Brazil, Malaysia, Saudi Arabia, Italy, Norway and the UK), we examine which modes of implementing novel carbon removal methods gain public support across diverse socio-political settings. We find that support hinges on procedural and distributive fairness: opening planning processes to public and expert scrutiny, benefit sharing and not-for-profit arrangements consistently increase public support for carbon removal across all countries and technologies. Respondents are unwilling to trade such fairness considerations for technical performance. Just governance and implementation are critical for securing public support when scaling up novel carbon removal methods.

## Abstract (web summary)

Public support is crucial for carbon removal deployment, yet the role of fairness remains unclear. Using surveys from six countries (*N* = 10,852), this study shows that procedural and distributive fairness are key determinants of support for carbon removal projects across diverse contexts.

## Main

Carbon dioxide removal (CDR) is increasingly recognized as a crucial component of strategies to address climate change, complementing deep system-wide decarbonization^1^ and transformative change in views, structures and practices^2^. Yet deploying CDR at the scale required to make a meaningful contribution to global mitigation goals raises pressing questions about how to do so in ways that are environmentally sustainable and socially just. This includes considerations of who bears the costs and who the benefits^3–6^, how responsibilities are allocated across and within countries^7,8^ and how implementation can reflect the needs and values of local communities and secure public support^9–12^.

As methods for removing atmospheric CO_2_ such as direct air carbon capture and storage (DACCS), bioenergy carbon capture and storage (BECCS) and enhanced rock weathering (ERW) move from research and pilot stages to deployment, there is a critical opportunity to align their implementation pathways with societal values and needs before technological and institutional arrangements become entrenched^13^. Comparative policy work shows currently low levels of governance and regulation for (novel) CDR in both member countries of the Organisation for Economic Co-operation and Development (OECD)^14^ and emerging economies^15^. Nonetheless, distinct types of nascent CDR policymaking have been identified, including incremental modification of existing national policy mixes, early integration and fungibility between emissions reductions and removals, proactive CDR policy entrepreneurship^14^ and repurposing policies, particularly those related to land use, land-use change and forestry^15^. Whereas different methods entail distinct sets of risks, benefits and cost profiles^16^, future CDR deployment overall will both shape and be shaped by nationally varying socio-political, economic and legal structures^17^.

Within these broader governance structures, public perceptions and responses can play a decisive role. Although public support is not always a prerequisite for deployment (for example, the US CO_2_ pipeline network built from the 1970s onward to serve enhanced oil recovery^18^ or subsea telecommunications cables^19^), public opposition can considerably slow down or even halt infrastructure deployment^20–22^, and (perceived) injustices can create public backlash to climate policies^23–25^. For example, the successful deployment of low-carbon electricity technologies depends on securing acceptance across three interconnected domains: socio-political acceptance among regulators and policymakers, market acceptance among financiers and firms and community acceptance among local host populations^26^. Failure to secure such acceptance can trigger organized opposition, manifesting in rallies, protests or litigation and ultimately leading to project delays, scope revisions, cancellation or compensation claims^27^. Beyond lessons drawn from other technologies, early signals of opposition to CDR projects, such as marine carbon removal experiments in the UK and Canada^28,29^ or the Coda Terminal for geological storage in Iceland^30^, underscore the importance of engaging with public concerns and expectations ‘upstream’^31^.

Understanding public perceptions of CDR is therefore essential for gauging its socio-political feasibility. To date, research has focused on how individual attitudes, beliefs and characteristics shape such perceptions. Relevant factors include the strength of climate beliefs, risk–benefit perceptions, trust in actors and institutions and concerns about mitigation deterrence or tampering with nature^32–38^. Such research finds that support depends on the CDR method assessed, with methods perceived as ‘natural’, such as afforestation, receiving more support than novel ‘engineered’ ones^36,39–45^. Qualitative research shows that public concerns are often tied to technology-specific trade-offs: land-use conflicts and distributive injustices for BECCS, energy costs for DACCS and mineral extraction and transportation burdens for ERW^46–48^. In addition, concerns about CO_2_ leakage, long-term storage integrity and broader environmental and community safety risks cut across multiple methods^10,35,46,47^, reinforcing perceptions that deployment entails not only technical but also social and ethical uncertainties. At the same time, survey research consistently finds that public familiarity with CDR methods remains low, with the exception of afforestation^32,49–51^. In such low-information contexts, opinions may rely more heavily on heuristics, analogies to other technologies or broader trust and fairness considerations than on detailed technical understanding.

Responses to novel technologies in the past suggest that support is contingent on the socio-technical and governance systems in which they are deployed and which influence how their prospective risks, benefits and opportunities are managed and distributed^52,53^. Lessons from renewable energy technologies show that deployment modes strongly influence public responses^54–57^. Anticipating public responses to CDR, thus, requires attention to the socio-technical configurations in which implementation unfolds^16,17,46^. Whereas insights from renewable energy provide an important starting point, CDR differs in several structurally crucial ways. Unlike renewables, CDR typically delivers diffuse and long-term climate benefits^58^ rather than salient local gains such as energy supply. Moreover, it is frequently entangled in debates about mitigation deterrence and moral hazard^59–61^, raising concerns that reliance on removals may delay emissions reductions. CDR is also often framed within broader discourses of geoengineering, which can evoke perceptions of technological overreach and tampering with nature^32,36,62^. These distinctive features complicate direct extrapolation from energy acceptance studies and underscore the need for targeted empirical investigation into CDR specifically.

Emerging evidence for the importance of socio-technical and governance systems in shaping public views of CDR predominantly comes from qualitative studies. Place-based research on ERW, ocean-based CDR and DACCS reveals that public support is contingent upon transparent decision-making, credible benefit sharing and clear allocation of responsibilities^9,10,63,64^. Deliberative exercises highlight public concerns about the unequal distribution of risks and benefits across communities and countries and emphasize the need for inclusive decision-making processes^11,47,65^. Quantitative research has begun to examine public perceptions of CDR implementation scenarios, though so far focused on few global north countries and few CDR methods^17,43,46^. How public support for CDR implementation relates to considerations of fairness remains unexplored, as do the interactions between governance and technical design features.

To address these gaps, we examine public support for different implementation pathways of novel CDR methods across six countries that vary markedly in political systems, political economies and geographic contexts: Brazil, Malaysia, Saudi Arabia, Italy, Norway and the UK. These countries span liberal democracies and autocratic political systems, include fossil fuel producers and diversified economies and represent global north and global south contexts where CDR governance remains nascent. By encompassing this institutional and socio-political diversity, we can assess whether fairness-related determinants of support are context specific or robust across heterogeneous settings. This comparative design strengthens the external validity of our findings and allows us to identify patterns that may generalize beyond single-country case studies.

## Results

To disentangle how socio-technical configurations shape public support for CDR, we conducted a multifactorial vignette experiment using nationally representative surveys of about 1,800 respondents in each country (total *N* = 10,852 respondents). Respondents were assigned on a least-fill basis to read a description, coupled with an illustration, of one of three CDR technologies (DACCS, BECCS, ERW; Supplementary Questionnaire 1). Respondents were then presented with five out of 36 different projects implementing that technology. The projects, or vignettes, differed along four socio-technical attributes, grounded in a multi-dimensional conception of justice^66^: implementing actor, decision-making process, profit distribution, carbon removal capacity. Respondents reported support for and perceived fairness of each project individually (Methods and Supplementary Note 2 show experimental design).

### Similar levels of tentative support across novel carbon removal technologies

**Fig. 1:**
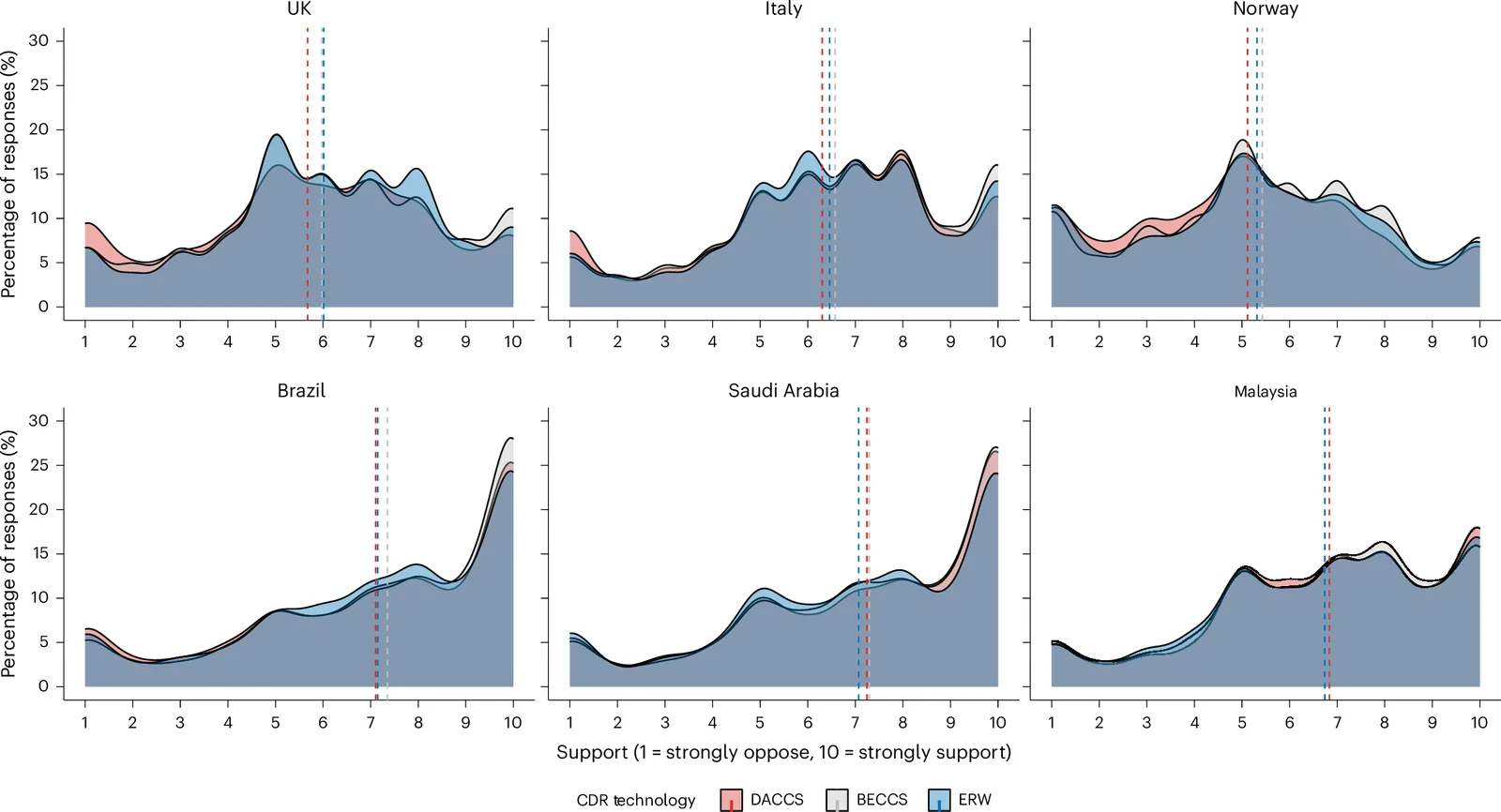
Average levels and distributions of support for novel carbon removal technologies across six countries. Density plots showing the distribution of respondents in terms of support (1 = ‘strongly oppose’, 10 = ‘strongly support’) for each CDR technology for all vignette evaluation tasks; this is based on around 3,000 evaluations per technology for each country, with a total of *N* = 54,260 observations (5 per 10,852 respondents). The *x* axis reflects all values of the support scale; *y* axis shows proportion of responses at each support level, in percentages. The areas under the coloured density curves add up to 100%, representing the full distribution of responses for each technology. The dashed vertical lines indicate the mean support score for each CDR technology within each country. Pairwise differences in mean support within and between countries for each technology were tested using two-tailed parametric Welch’s *t*-tests with Bonferroni correction applied for multiple comparisons. Within countries, mean support scores of the three novel CDR technologies are significantly different (Bonferroni adjusted *p* < 0.05), with the following exceptions: BECCS vs ERW in UK (*p* = 1), Italy (*p* = 0.20) and Norway (*p* = 0.30); DACCS vs ERW in Italy (*p* = 0.05), Brazil (*p* = 1) and Saudi Arabia (*p* = 0.07); DACCS vs BECCS in Saudi Arabia (*p* = 1); all CDR technologies in Malaysia (DACCS vs BECCS, *p* = 0.42; DACCS vs ERW, *p* = 0.37, BECCS vs ERW, *p* = 1). Between countries, pairwise differences of the mean support scores are statistically significantly different (*p* < 0.05) for each technology except for all CDR technologies in Brazil vs Saudi Arabia (*p* = 1); BECCS in Italy vs Malaysia (*p* = 0.47). Complete results and significance tests are reported in Supplementary Tables 2–5.

We find comparable levels of support across CDR technologies in each of the surveyed countries, based on all the vignettes presented to the respondents (Fig. 1; full tables in Supplementary Tables 2 and 3). Whereas all three technologies are, tentatively, supported (mean support scores between 5.12 and 7.35, standard deviations (sd) between 2.44 and 2.88), a few differences are noteworthy: DACCS receives lower support among European respondents than BECCS and ERW (for example, UK DACCS mean = 5.67, sd = 2.63, ERW mean = 6.01, sd = 2.44), and this difference is consistently statistically significant (*p* < 0.05). In the non-European countries, differences in support for the three technologies are generally modest. In Brazil, BECCS receives statistically significant higher support than DACCS and ERW, and in Saudi Arabia, it receives significantly higher support than ERW. In Malaysia, we find no statistically significant differences in support for the three technologies.

Comparing support across countries, we identify two distinct profiles: those with broadly high support and those with more moderate support accompanied by higher levels of opposition. Irrespective of the technology, support is higher in Brazil, Saudi Arabia (means between 7.07 and 7.3; sd ≈ 2.8) and to a lesser extent Malaysia (means ≈ 6.8; sd ≈ 2.5). In European countries, support is more moderate, with Norwegian respondents reporting the lowest levels of support (DACCS mean = 5.12, sd = 2.62; BECCS mean = 5.43, sd = 2.57) and Italian respondents reporting the highest (DACCS mean = 6.30, sd = 2.59; BECCS mean = 6.57, sd = 2.52). Support for BECCS is similar in Italy and Malaysia—the only such overlap between global north and global south countries. When interpreting these results, cross-cultural variation in response tendencies deserves careful consideration. The pronounced skew towards high support particularly for Brazil and Saudi Arabia may reflect contextual differences, cross-cultural response styles, social desirability or other unobserved factors. Whereas our design and quality-control procedures reduce concerns about data-quality artefacts, they cannot eliminate them. We therefore interpret cross-country differences in absolute support cautiously and emphasize the marginal effects of experimentally varied vignette attributes.

### Procedural and distributive fairness increase public support for carbon removal

We calculated the average marginal component effects (AMCEs) of the four project attributes on support for CDR projects, using separate ordinary least squares regressions for each country and clustered standard errors at the individual level. We present results obtained from pooling responses for DACCS, BECCS and ERW. Technology-specific analyses reveal no meaningful divergences (Supplementary Fig. 1 and Supplementary Tables 6 and 7): the relative importance of socio-technical features is remarkably consistent across technologies.

**Fig. 2:**
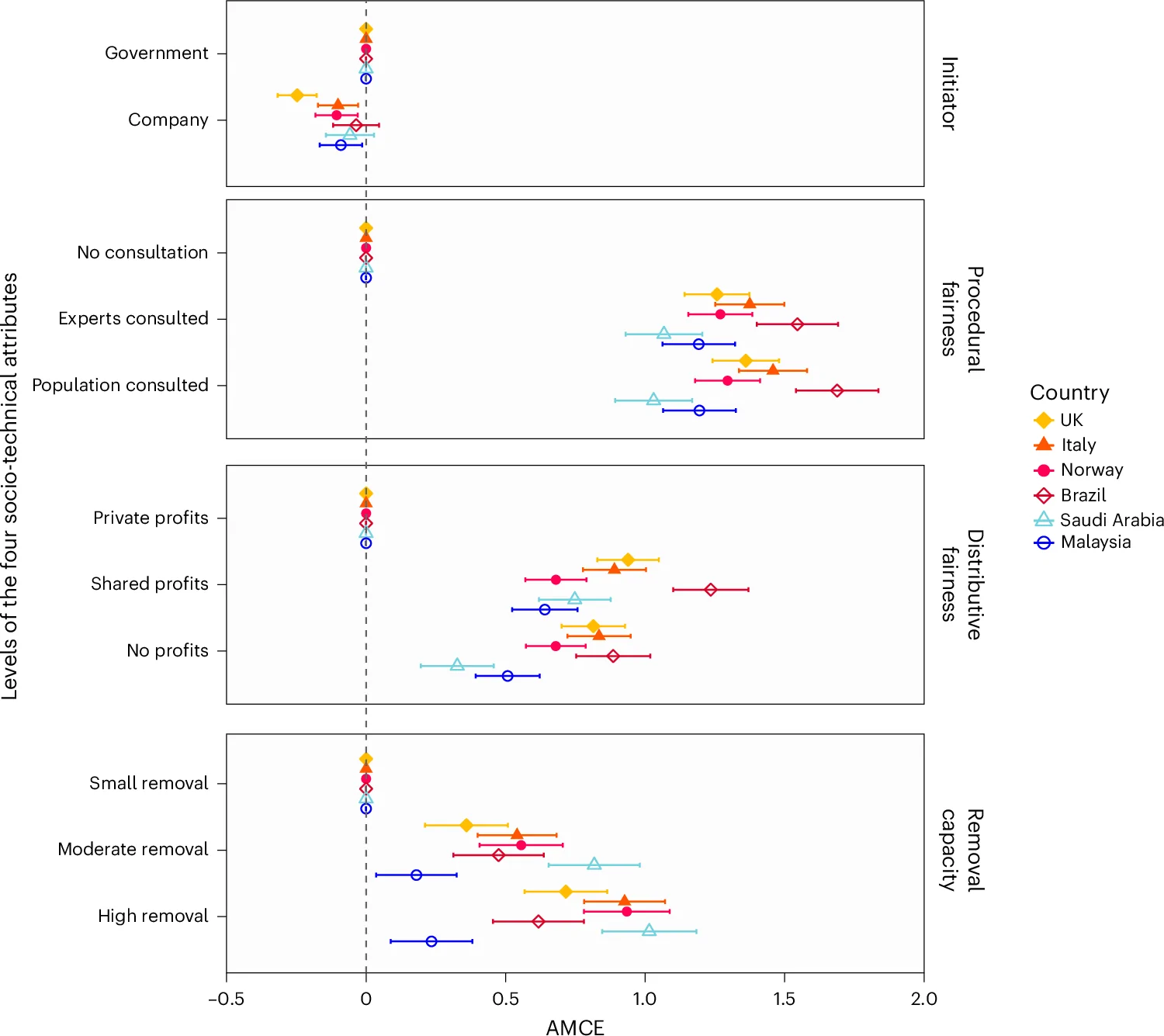
AMCEs of governance and technical attributes on public support for carbon removal projects across six countries. *N* = 10,852 respondents, with a total of 54,260 observations (five per participant). AMCE (unstandardized effect sizes) for the socio-technical attributes of the vignette experiments calculated using ordinary least squares regressions (one per country) with individual clustered standard errors. *Y* axis includes the levels of the four socio-technical attributes. *X* axis shows AMCEs on the support scale (1 = ‘strongly oppose’, 10 = ‘strongly support’). The dashed vertical line highlights the base level 0.0 (that is, for the base attribute levels: Government, No Consultation, Private Profits, Small Removal). The mean value of the AMCE is represented by the respective symbol for each country and lines represent the 95% confidence intervals. No adjustment for multiple comparisons was applied. Exact *p*-values and additional regression statistics are reported in Supplementary Table 8.

Across all countries, procedural fairness has the strongest effect on public support for CDR projects (Fig. 2). Compared to no consultation being undertaken, opening the decision-making process through consultation of local communities or scientific experts, substantially increases support by around 1.0 to 1.7 points on a 1–10 scale. We find little evidence of systematic differences between consultation of local communities and consultation of scientific experts. Procedural fairness has the largest effect in Brazil (‘experts consulted’ estimate = 1.54, confidence interval = [1.40–1.69]; ‘population consulted’ 1.68 [1.54–1.83]) and the smallest in Saudi Arabia (‘experts consulted’ 1.06 [0.92–1.20]; ‘population consulted’ 1.03 [0.89–1.17]). Despite currently limited opportunities for public consultations in the Saudi Arabian context, the effect of procedural fairness on support is substantial and, in absolute terms, not far from those observed in Malaysia or Norway. This should not be interpreted as evidence that consultation is understood identically across political contexts; it shows only that the experimentally specified contrast between no opinion-seeking and opinion-seeking increased support in Saudi Arabia as elsewhere.

The second most important driver of public support is distributive fairness. Compared to projects where the developer retains all profits, alternative ways of handling profits lead to more favourable evaluations, with some differences between countries. Revenue redistribution via direct payments to local communities increases support by around 0.50 to 1.25 scale points. Overall, we find minimal differences in support at the country level between the two profit-sharing alternatives. The effect of profit sharing is largest in Brazil (1.23 [1.10–1.37]), followed by the UK and Italy. Not-for-profit set-ups where CDR is conducted as a public service without generating profits also increase support, though less so in Saudi Arabia (0.33 [0.20–0.46]).

Although less relevant than fairness considerations, the project’s removal capacity also plays a role in respondents’ evaluations: the higher the amount of CO_2_ removed, the more support a project secures. Removal capacity matters the most in Saudi Arabia, Italy and Norway. Especially, in Saudi Arabia, the marginal effect of a project’s capacity for high CO_2_ removal increases support by 1 scale point [0.83–1.17], outweighing the effect of distributive fairness attributes, associated with a marginal increase of 0.33 [0.20–0.46] for a project running as a public service and 0.75 [0.62–0.88] for a project redistributing revenues locally. Whereas support grows with removal capacity, the increase is not linear. Some countries (Malaysia, Brazil) show minimal difference in how a larger removal capacity influences support. The effect of removal capacity on support is weakest in Malaysia, where the capacity to remove a high amount of CO_2_ increases support by only 0.23 [0.08–0.37] scale points.

Finally, whether a CDR project is carried out by a company or by a regional government does not substantially affect public support. The actor attribute matters most in the UK, where company implementation reduces support by −0.24 [−0.32 to −0.18] points. Although the effect remains small, a similar negative impact of company involvement is observed in Italy, Norway and Malaysia.

The socio-technical features in our experiment are relevant for respondents’ assessments irrespective of whether they would oppose or support CDR projects in ‘their backyard’. Using responses to a post-experiment item measuring support for CDR projects located within 10 km of respondents’ homes, we divide the country sub-samples into three groups: opponents, moderates and supporters of CDR in ‘their backyard’ (Supplementary Note 3 and Supplementary Tables 9 and 10). Respondents evaluated only the local implementation of the CDR technology to which they were assigned in the experiment. We observe similar preference structures in the experiment results among both ‘supporters’ and ‘opponents’ of CDR in ‘their backyard’ (Supplementary Fig. 2 and Supplementary Table 11). Opponents respond similarly, or even more strongly, than moderates and supporters, to both the governance and the technical attributes. That is, opponents do not outright reject CDR but are responsive to how projects are developed. This result provides further evidence that support is conditional on careful design of implementation.

### High carbon removal capacity cannot compensate for lack of fairness

We next considered trade-offs and tested whether a high removal capacity can buy developers out of due consideration of fairness in project implementation. Using ordinary least squares regressions including interactions of removal capacity with the decision-making attribute and the profit distribution attribute, we confirm the high importance of fairness considerations, irrespective of the amount of carbon a project promises to remove.

**Fig. 3:**
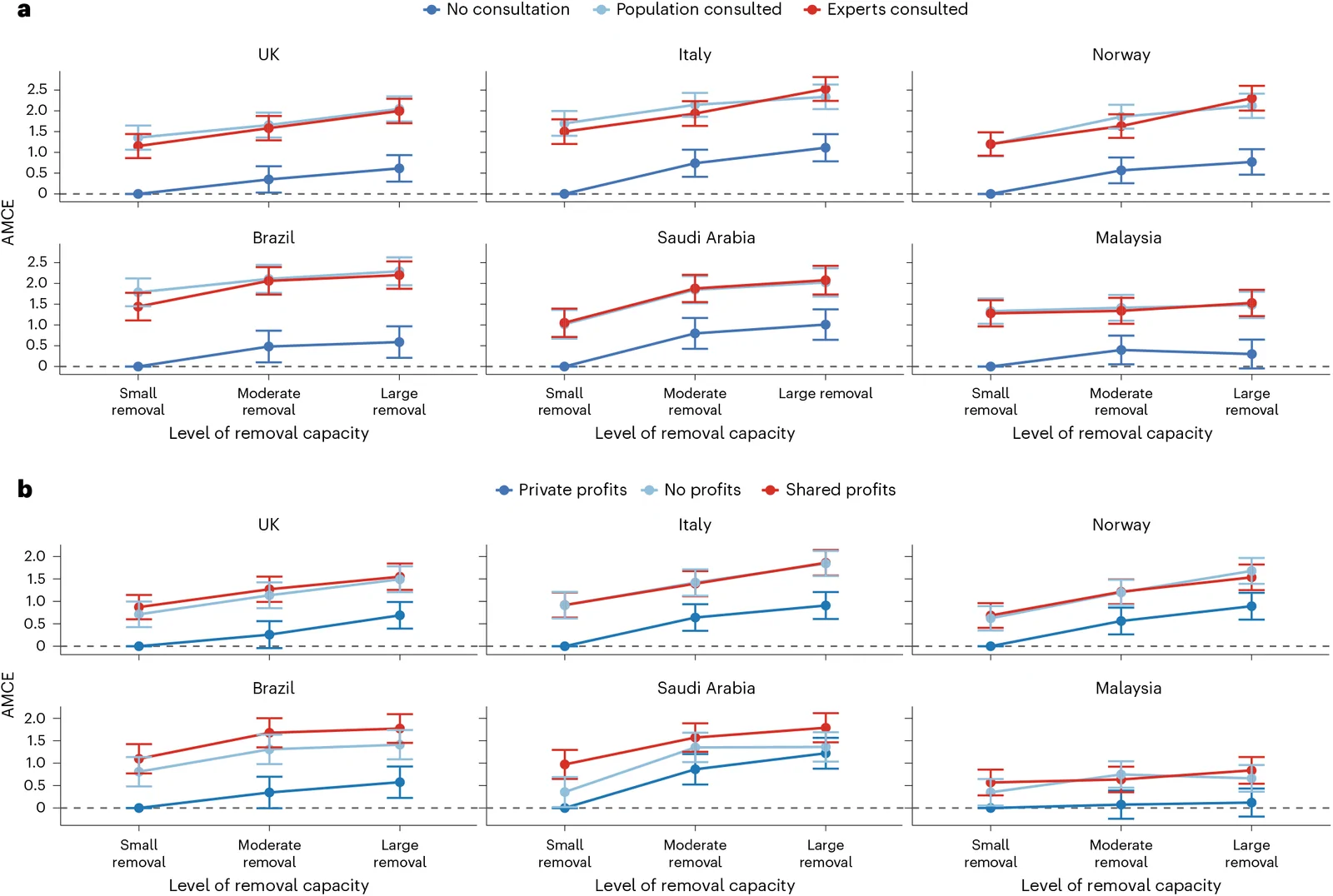
Interaction effects between technical attributes of carbon removal projects and procedural fairness or distributive fairness across six countries. *N* = 10,852 respondents, with total of 54,260 observations (five per participant). **a**,**b**, AMCEs of the interaction between removal capacity and attributes for consultation during the decision-making process (**a**) or distribution of profits (**b**). Calculations based on ordinary least squares regressions (one per country) with individual clustered standard errors across countries support as the dependent variable and the full set of attributes as predictors. *Y* axis shows AMCEs for support (1 = ‘strongly oppose’, 10 = ‘strongly support’). *X* axis relates effect to the levels of the removal capacity attribute. Dashed horizontal lines highlight the reference values 0.0 (for the base attribute levels: Small Removal and No Consultation (**a**), Small Removal and Private Profits (**b**)). The mean value of the AMCE is represented by the dots (for each combination in each country) and vertical lines represent 95% confidence intervals. Connecting lines only intended to ease visual comparison of the interactions. No adjustment for multiple comparisons was applied. Exact *p*-values and additional regression statistics are reported in Supplementary Table 12.

Across all countries, respondents reward projects that are both effective and fairly governed, but these two considerations largely operate independently (Fig. 3). The levels of support differ between the decision-making process (Fig. 3a) and profit distribution (Fig. 3b) attributes, whereas the effect of increasing CO_2_ removal is remarkably similar regardless of consultation or profit-sharing conditions. This pattern is visible for all countries except Malaysia and Saudi Arabia. The Malaysian results can be explained by the low importance of removal capacity in this context. The same applies to the lack of statistical significance in the effects of profit distribution in Saudi Arabia.

This analysis suggests that to garner public support attention to both technical and governance features of CDR projects is key. High removal capacities cannot compensate for a lack of consultation or the privatization of profits. The opposite can also be said. From the perspective of public support, it is not possible to compensate for a project’s small removal capacity with progressive project governance.

### Active roles for local communities wanted, particularly in European countries

**Fig. 4:**
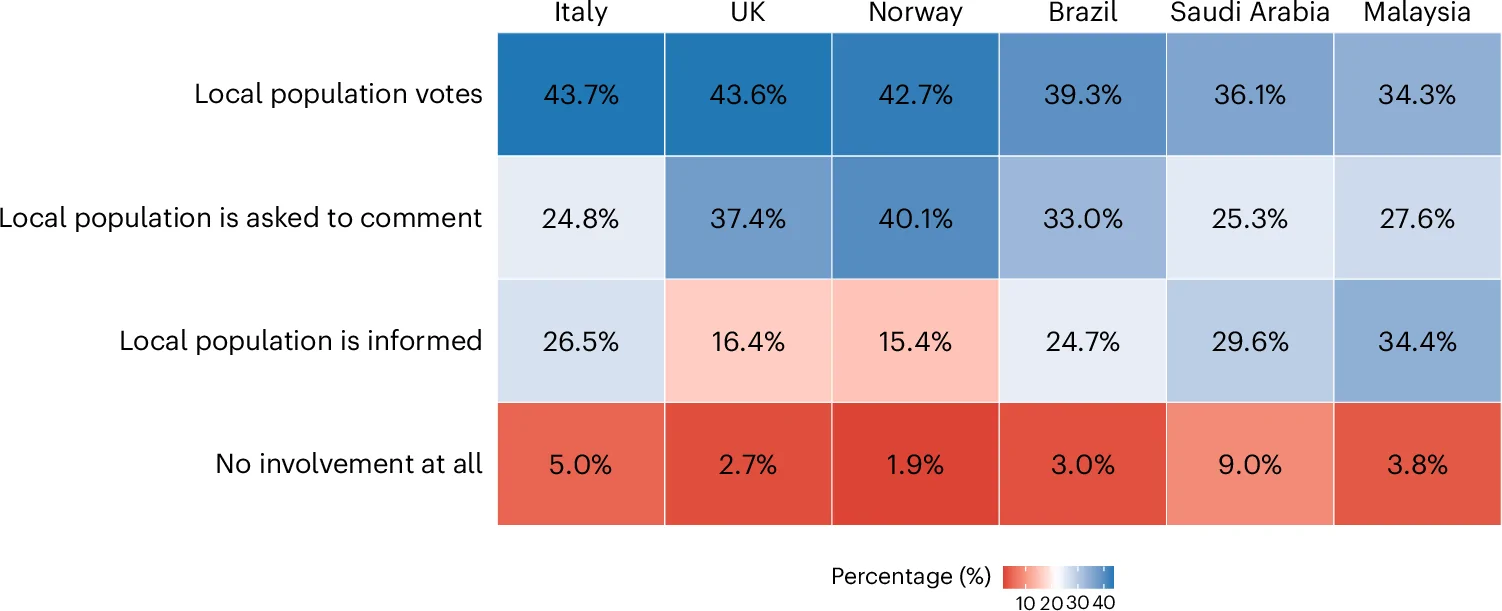
Public preferences for fairly engaging local communities in governance of carbon removal projects, across countries. *N* = 10,526 respondents. After completing all the vignette evaluations, respondents were asked “When starting a new [DACCS/BECCS/ERW] project, there are various decisions to take and different ways to involve the local population. When it comes to the planning of such a project, what level of involvement of the local population do you think is fair for each of the following decisions?” This question was asked for three decisions: (**1**) whether the project is permitted, (**2**) the location where to implement the project, (**3**) how to distribute profits. Only decision (**1**) shown in the figure; the other two are included in Supplementary Figs. 3 and 4. ‘Don’t know’ responses (*n* = 326 respondents) have not been included in the analysis. Each cell shows the percentage of respondents, in each country, that selected this type of involvement as most fair.

The decision-making attribute in the survey experiment was complemented by an item on preferred degrees of engaging the public in planning and implementing CDR projects. We identified a preference for higher degrees of engagement—notably, for the public to have a vote on key decisions—in the western democracies included in our sample. When it comes to permitting CDR projects, allocating decision-making authority to local communities is the fairest option for around 44% of respondents in the UK and Italy, 43% in Norway versus 39% in Brazil, 36% in Saudi Arabia and 34% in Malaysia (Fig. 4). Only a small fraction of respondents (ranging from 2% in Norway to 9% in Saudi Arabia) considers no involvement of local communities to be the fairest option. This confirms the general preference for engagement expressed in the vignettes, while showing how preferred degrees vary across socio-political contexts. Comparing responses across types of decision, we find only minimal differences. Across countries, respondents do not strongly differentiate whether engagement happens in relation to permitting, siting or the distribution of profits, although we do observe a slightly lower desire for direct involvement in decisions on how to distribute profits (Supplementary Figs. 3 and 4).

### Effects of individual characteristics and attitudes

**Fig. 5:**
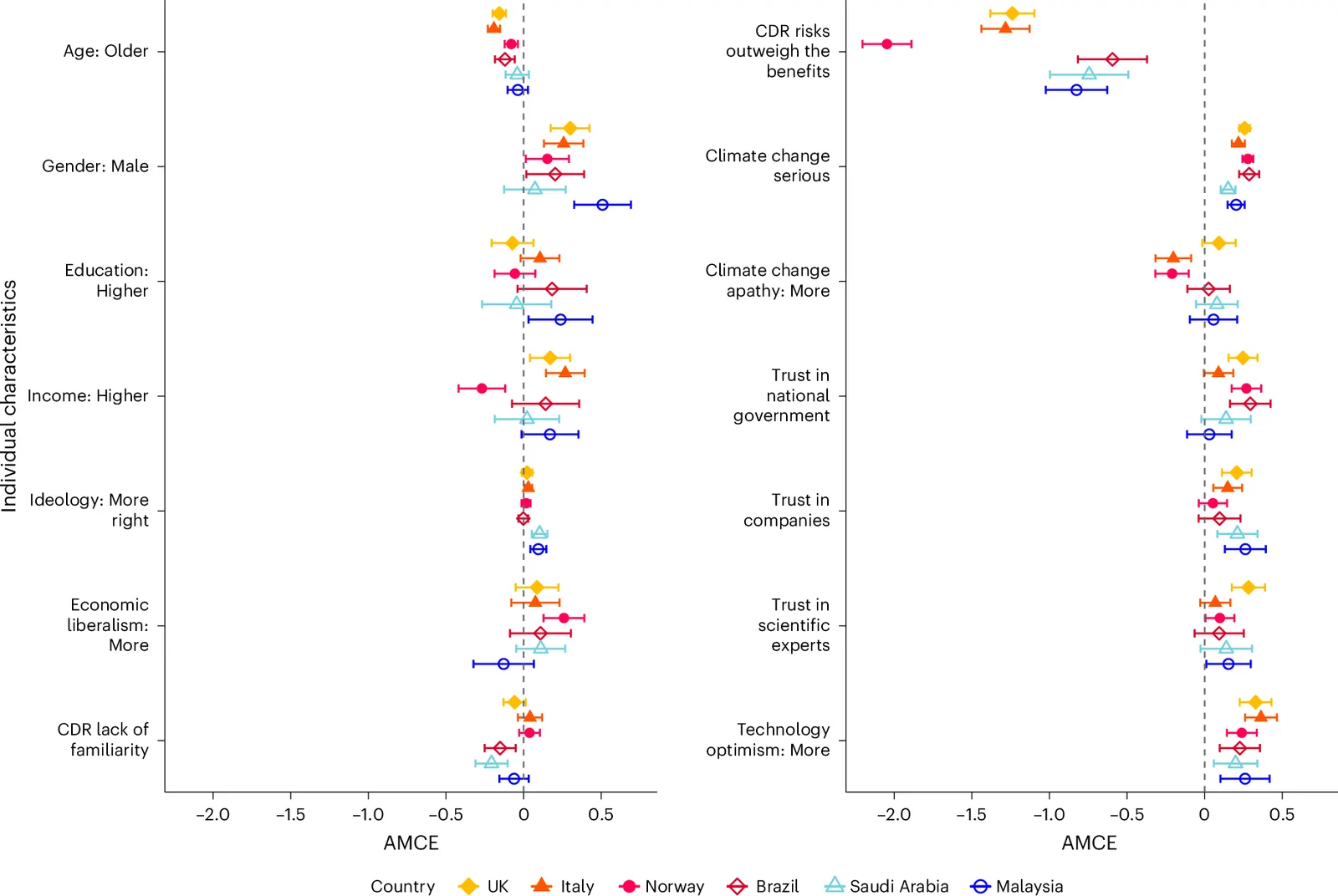
AMCEs of respondents’ selected individual characteristics on support for carbon removal projects across countries. *N* = 7,501 respondents, with total of 37,505 observations (five per participant). The lower number of observations is due to the exclusion from the analysis of observations with missing values in at least one of the covariates. The *y* axis presents the individual characteristics included in the analysis. The *x* axis shows the average change in effect on support (1 = ‘strongly oppose’, 10 = ‘strongly support’). The dashed vertical line highlights the baseline value 0.0 (reference values not shown). The mean value of the AMCEs is represented by the respective symbol for each country, and lines represent the 95% confidence intervals. The effects of the vignette attributes are not shown. No adjustment for multiple comparisons was applied. Exact *p*-values and additional regression statistics are reported in Supplementary Table 16.

Random intercept multilevel models show that in each country a substantial share of the variation in support across vignettes is explained by individual characteristics (intra-class correlation coefficients between 0.37 in Saudi Arabia and 0.52 in Norway; Supplementary Table 13). Thus, we explore how individual socio-demographic characteristics and attitudes relate to support for the CDR projects in the experiment. We investigate this by including individual-level items in the main ordinary least squares regressions with individual clustered standard errors (Supplementary Note 4 and Supplementary Table 14 provide covariates). Inspection of pairwise correlations among the covariates and variance inflation factors in each country denotes only moderate multicollinearity, which does not require any of the factors to be removed (Supplementary Fig. 5 and Supplementary Table 15). Consistent with previous research^32–35^, risk perception, concerns about climate change, trust in relevant actors and techno-optimism are the strongest correlates of support (Fig. 5). Socio-demographic factors also matter, though their effects are smaller and less consistent across countries.

Strikingly, political orientation exerts only limited influence on support for novel CDR implementation, especially when contrasted with its well-documented role in shaping climate attitudes more broadly^67^. In most countries, left–right ideology and economic liberalism show near-zero associations with support, suggesting that CDR has not yet become politicized in the way climate mitigation often is. This relative insulation from partisan divides may reflect the novelty of CDR methods or the fact that public judgements are driven more by perceptions of risk, effectiveness and trust than by political worldviews.

## Discussion and conclusion

In this article, we presented the results of a cross-country, experimental survey on drivers of public support for the implementation of CDR. We specifically illustrate how socio-technical and governance systems matter for deployment and establish that procedural and distributive fairness are crucial determinants of public support for novel CDR projects. These results are broadly robust across the six countries with diverse political systems and political economies, across constituencies and for all CDR technologies included in our study.

The results presented, and particularly the limited political polarization and moderate support, need to be interpreted in light of low public familiarity and only nascent CDR sectors in the six countries. Experiences with other climate-mitigation technologies caution against reading these patterns as stable equilibria. Extrapolating from energy technologies^26,68^, controversies and polarization will probably become more marked as real-world implementation advances, especially when trade-offs, costs and distributional implications become tangible or projects become entangled with incumbent interests or partisan narratives. The relative insulation of CDR from ideological polarization observed in our data may therefore reflect its current novelty rather than entrenched consensus. Because CDR governance will not emerge from a blank slate—and may instead adapt or repurpose existing policy instruments^15^—understanding how historical developments and current debates in adjacent policy fields influence perceptions of CDR is crucial. For example, conflict around biofuels^69^ might spill over to debates on BECCS, and national regulatory contexts and controversies on CCS^70^ or other technologies such as shale gas fracturing^71^ and nuclear waste^47^ might shape perceptions of CDR methods involving geological storage. In addition to such ‘technology spillovers’, ‘historical spillovers’ from past injustices and negative experiences with extractivist and pollutive industries such as mining, oil and gas^28,64,72^ require attention. Identifying potential nationally distinct controversy spillovers^73^ early on can help policymakers and developers anticipate and prepare for contestations around CDR.

Aligning emerging CDR policies and strategies with principles of justice will be key for socially robust implementation pathways. Our findings offer four core takeaways for the governance and deployment of CDR technologies:

First, promoting procedural fairness by opening up decision processes to public and expert scrutiny consistently increases support for CDR projects. These findings corroborate qualitative evidence showing that publics envision an active role for themselves^9,11,46^, while also having high expectations towards experts in the governance of climate technologies^74,75^. Whereas publics clearly demand some form of engagement in the implementation of CDR, in the current context where any of these technologies are still largely unfamiliar to the public^32,36,49^, exact modes and timings of engagement are not at the forefront of their concerns. This opens space for societal deliberation and experimentation with engagement formats tailored to specific places and contexts^76^. What engagement—operationalized in our experiment as asking for and listening to the opinions of local populations—means to publics in diverse political systems, particularly in authoritarian contexts, requires further attention and qualitative, deliberative research. Furthermore, community-level engagement activities should be accompanied by socio-political measures for building and restoring trust in industry and governmental actors, which our study confirmed as an important prerequisite for public support of climate technologies^33,35,62,77^.

Second, pursuing distributive fairness and not-for profit arrangements consistently lead to higher support. CDR projects that privatize profits garner the least support in our analysis. Our study thus contributes empirical insights into how the public views expert propositions to treat CDR as a public or common good whose governance should be inspired by public utility models^4,13,78^. Public preferences for the broader sharing of profits and not-for-profit approaches suggest an openness towards exploring alternatives to private sector-led removals. Future research and societal deliberation are needed in pursuit of alternative visions and governance systems.

Third, the public does not show willingness to trade off fairness considerations for technical performance. Even if CDR is fundamentally motivated by climate considerations, these results underscore that the long-term efficacy and legitimacy of implementing climate technologies also depend on social considerations. As such, the complexities of deploying CDR call for integrated socio-technical perspectives and interdisciplinary collaborations^79,80^.

Fourth, tensions between the need for accelerating deployment to close critical CDR gaps^16,81^ and the safeguarding of procedural considerations warrant attention moving forward^82^. Responding to the preferences for public engagement highlighted in this study raises questions about how to do so meaningfully and effectively. Project-based and developer-led engagement^83^ might not be best suited for ensuring just and responsible deployment in the given timeframes. Instead, policymakers could experiment with regional-level and systemic engagement beyond discrete projects and at planning rather than implementation stages. Such upstream practices could allow for deliberating place-based scenarios for CDR in the context of wider climate action portfolios, each with their respective trade-offs around land use, costs or efficacy from a mitigation perspective, and for productively engaging with controversy as an opportunity for learning^84^ rather than as a problem downstream. Furthermore, the preference for non-profit configurations points towards the consistently low trust in private companies^85^ and the tension this creates for developing business models and raising the investments to deploy CDR at scale. Navigating the inherent tensions between urgency, low trust and due process also calls for innovations in impact assessment practices in the context of the climate transition^86^. Taken together, these tensions underscore the need for a prudent approach to estimating the future scale and pace of CDR deployment, avoiding overreliance on promises of future removals at the expense of emissions reductions today^3^.

## Methods

### Country selection

The survey was conducted in six countries: three European (Italy, Norway, the UK) and three non-European (Brazil, Malaysia, Saudi Arabia) countries. Country selection aimed to capture variation in geography, political systems and political economies, while focusing on contexts that share both considerable fossil fuel sectors and potential for CDR deployment. This combination provides a strong basis for assessing the context specificity—or robustness—of public preferences for implementation arrangements in settings where the socio-technical and political stakes of large-scale deployment may be particularly high in the future (detailed selection criteria in Supplementary Note 2).

Our experimental design does not estimate the effects of national policy trajectories directly; rather, it tests whether selected implementation attributes are evaluated similarly or differently across countries whose CDR pathways, governance and incentives are nascent. Whereas the European Union and the UK have pledged land use, land-use change and forestry (LULUCF) targets until 2030, Norway, Brazil, Malaysia and Saudi Arabia have not quantified removals. At present, only the UK distinguishes explicitly between emissions reductions and removals, with a target to deploy 5 MtCO_2_ of novel CDR by 2030. Whereas small-scale pilot and research and development projects for novel CDR exist in all six countries, national policy landscapes and discourse are at best emerging (a short overview of national variations in CDR pathways is in Supplementary Note 1).

### Data collection and sampling

The questionnaire was administered by Norstat Denmark, a professional data-collection company. The company recruited participants from national online panels and took care of coding, translating, distributing and administering the survey. Quotas for national representativeness in terms of age and gender, along with broad quotas for education, have been defined according to the survey provider’s possibilities. Moreover, in each country, specific quotas for geographic regions were set to avoid oversampling from specific areas. As with gender and age quotas, these regional quotas reflected the population distribution in each country. Researchers were provided with documentation showing how the final sample matched these population targets, which they deemed appropriate. In addition, to participate in the survey, respondents had to be between 18 and 75 and able to understand the language of survey administration. The survey was distributed in the respective national languages (English, Italian, Norwegian, Portuguese–Brazilian, Arabic, Malay) with an additional English-language option in Malaysia and Saudi Arabia. Data collection took place between 23 June and 21 July 2025, and the final dataset was received by Aarhus University on 22 July 2025.

The data-collection company recruited participants through their online panels. Panel members are invited to join the survey based on set quotas for each country and join voluntarily. Although online panels allow rapid data collection and targeted quota matching, they introduce certain limitations: respondents must have an internet connection and a suitable digital device, thus, they are typically more engaged online and more accustomed to survey taking. These characteristics might imply that they differ from the general population in unobserved ways. To mitigate these drawbacks, the researchers and the survey provider agreed on strict quality-control procedures such as three attention checks, response-time monitoring and removal of low-quality responses. These measures were aimed at enhancing data reliability, yet it remains possible that the panel-based recruitment influenced data quality by favouring individuals more familiar with survey environments.

### Experimental design and vignette attributes

In the first step, respondents were allocated to one of three CDR methods: DACCS, BECCS or ERW. Allocation to these groups was based on a least-fill criterion, meaning respondents were allocated to whichever method had the fewest respondents when they entered the survey. The allocation also accounted for age, region and gender of respondents to ensure a broadly balanced demographic distribution across each CDR method. Each of these groups is composed of around 600 respondents per country (quota information for the full sample and each sub-sample in Supplementary Tables 17 and 18). Respondents were provided with information texts on the respective CDR method, describing in simple terms how it worked and outlining potential benefits and risks (Supplementary Questionnaire 1). Different use cases of the method and varying roles in the wider portfolio of climate-mitigation measures (for example, using CDR for counterbalancing residual emissions or reversing temperature overshoot) were not introduced to respondents. Following these descriptions, respondents were presented with one scenario (that is, vignette) at a time for which they reported their opposition or support and rated who fair they thought it was on two numeric scales ranging from 1 (strongly oppose / very unfair) to 10 (strongly support / very fair).

Two of the four attributes in the vignette experiment centred on justice considerations in the implementation of CDR, including distributive and procedural dimensions (Supplementary Note 2 provides conceptual background). These attributes are composed of three levels. Decision-making process: our baseline is a decision by the respective authorities without any consultation, and the other two include the opening up of the decision-making process, one through consultation of the affected local population and the other through consultation of scientific experts. Distribution of profits: one level corresponds to the ‘business as usual’ set-up, another corresponds to the redistribution of profits between the implementer and the affected local population and the last builds on work of scholars exploring possibilities for treating CDR as public good and correspond to the absence of any profit^4,13^. Two additional attributes were included in the experimental vignette to contextualize the justice considerations. Implementer of the projects: composed of two levels, company or regional government. Carbon removal capacity of the projects: small, moderate or high. To make this attribute relatable to the lay public, we have chosen the number of passenger cars whose emissions could be removed by the corresponding project. The calculations assumed that a mid-range car emits 18 kg of CO_2_ per 100 km and runs for an average of 10.000 km per year (refs. ^87,88^). Both information on the implementers and the carbon removal efficacy of the projects have been employed in previous studies on public perceptions of CDR methods and have shown mixed results^17,43,77^.

In a full factorial design for these attribute levels, there would have been 54 possible scenarios (2 × 3 × 3 × 3). We reduced the number of scenarios by employing a fractional factorial design with fold over^89^. With this design, including orthogonality and level balance, we were able to separate main effects and two-way interactions. This resulted in 36 vignettes, the same for each CDR technology. Respondents were randomly assigned five of these vignettes without replacement, although with some backend constraints making sure each attribute level was shown at least once, except for removal capacity on which there was no constraint. Programming the allocation of vignettes this way made the distinction between the vignettes easier for respondents while guaranteeing random allocation of respondents to vignettes. Each vignette was assessed around 250 times per country.

### Data analysis

The analysis follows the preregistration protocol. To assess the impact of the attributes, we computed average marginal component effects (AMCE) using ordinary least squares regressions with individual clustered standard errors^56,90^. Given the randomized set-up of the experiment, no control variables were necessary. These results are shown in Fig. 2 and the full tables in the Supplementary Information. To test the robustness of the results, we ran similar models excluding respondents who selected the same level of support across all five vignettes; this rendered similar results (Supplementary Tables 19 and 20). The effects shown in Fig. 3 were estimated in the same way; one model included an interaction between the attributes on removal capacity and decision-making process (Fig. 3a), and the other model an interaction between removal capacity and profit distribution (Fig. 3b). Figure 5 was produced by adding the shown covariates to the model in Fig. 2 to observe their main effects. All analysis was performed using R command *lm* from the basic package stats for the main regressions and the package ggplot2 for visualizations.

Several caveats of cross-country survey research are noteworthy. Response styles may differ across countries. For instance, Asian respondents are less likely to choose extreme response categories and more prone to acquiescence bias, that is, agreeing with statements or responding affirmatively, compared to western respondents^91,92^. Caution is, thus, warranted when interpreting responses across contexts as they may partly reflect cultural differences in survey behaviour, rather than underlying preferences. This applies to the interpretation of our findings on overall support. Figure 1 shows higher average support for CDR technologies in global south countries compared to global north countries, consistent with previous studies^49^. Response-style differences may partly explain this pattern, although our design does not allow us to fully separate response-style effects from substantive contextual differences. As we are aware of this issue, we reported level differences but focused on the interpretation of the differences and similarities in the marginal effects of the vignette attributes. Cross-country survey research, furthermore, carries the risk that key concepts may not be interpreted equivalently across contexts. When formulating the vignette attributes (Supplementary Questionnaire 1), we paid due attention to wording that would work across contexts. For example, instead of speaking of ‘public consultation’, we described opening the decision process in the following way: “This project is permitted by the authorities without asking/after listening to the opinion of the affected local population”. However, the caveat remains that respondents in democratic vs authoritarian contexts might envision such processes differently. Future qualitative research is needed to elicit more nuanced, context-specific understandings and expectations regarding formats of public engagement, especially in relation to the emerging area of CDR governance.

### Ethics statement

The study has been approved by the Aarhus University’s Research Ethics Committee for the faculty of Business and Social Sciences (Institutional Review Board) in accordance with the guidelines of the university’s Research Ethics Committee (approval number: BSS-2024-121-S2). All survey respondents provided their full and informed consent.

### Reporting summary

Further information on research design is available in the Nature Portfolio Reporting Summary linked to this article.

## Online content

Any methods, additional references, Nature Portfolio reporting summaries, source data, extended data, supplementary information, acknowledgements, peer review information; details of author contributions and competing interests; and statements of data and code availability are available at https://doi.org/10.1038/s41558-026-02741-7.

## Supplementary information

Supplementary Information Supplementary Figs. 1–5, Tables 1–20, Questionnaire 1 and references.

Reporting Summary

Peer Review File

## Data Availability

Preregistration information and the data that support the findings of this study are available at https://doi.org/10.17605/OSF.IO/2XGC6 (ref. ^93^).

## Code availability

Replicating this study requires R and RStudio (R version 4.4.2, RStudio version 2024.10.31 were used). All code is available at https://doi.org/10.17605/OSF.IO/2XGC6.
