# Supplementary material for: Public support for novel carbon removal hinges on procedural and distributive fairness
Source: Nat Clim Chang. 2026 Sep 24;16(10):1152–61. doi: 10.1038/s41558-026-02741-7 (PMC13638447; doi:10.1038/s41558-026-02741-7)
Supplement: Supplementary file 1 [file 41558_2026_2741_MOESM1_ESM.pdf]

nature climate change

Supplementary information https://doi.org/10.1038/s41558-026-02741-7

Public support for novel carbon removal hinges on procedural and distributive fairness

In the format provided by the authors and unedited

Table of Contents

I. Further information on experimental procedure 4

Supplementary Note 1. CDR technology and country selection. 4

Supplementary Note 2. Conceptual background informing the vignette design. 5

Supplementary Table 1. Key elements of the experimental vignette. 5

II. Figure 1 Corresponding Tables 6

Supplementary Table 2. Percentages of responses for support for novel carbon removal technologies (Direct Air Capture (DACCS); Bioenergy with Carbon Capture and Storage (BECCS); Enhanced Rock Weathering (ERW)) across six countries (1= strongly oppose; 10=strongly support). 6

Supplementary Table 3. Means of support for novel carbon removal technologies (Direct Air Capture (DACCS); Bioenergy with Carbon Capture and Storage (BECCS); Enhanced Rock Weathering (ERW)) across six countries (1= strongly oppose; 10= strongly support). 6

Supplementary Table 4. Within-countries two-tailed parametric Welch's t-tests with Bonferroni correction applied for multiple comparisons of means of support for novel carbon removal technologies. 7

Supplementary Table 5. Between-countries two-tailed parametric Welch's t-tests with Bonferroni correction applied for multiple comparisons of means of support for novel carbon removal technologies. 8

III. Vignette Results across CDR Technologies and Countries on Support and Fairness 9

Supplementary Figure 1. Average marginal component effects of governance and technical features on public support and perception of fairness for carbon removal projects (1-10 scale: 1= strongly oppose; 10=strongly support) across six countries and carbon removal technologies. 9

Supplementary Table 6. Estimated average marginal component effects of governance and technical features on public support for carbon removal projects (1-10 scale: 1= strongly oppose; 10=strongly support) across six countries and carbon removal technologies. 9

Supplementary Table 7. Estimated average marginal component effects of governance and technical features on public perception of fairness for carbon removal projects (1-10 scale: 1= very unfair; 10=very fair) across six countries and carbon removal technologies. 13

IV. Figure 2 Corresponding Table 17

Supplementary Table 8. Estimated average marginal component effects of governance and technical features on public support for carbon removal projects (1-10 scale: 1= strongly oppose; 10=strongly support) across six countries. 17

V. CDR in Backyard 19

Supplementary Note 3. Operationalization of CDR in Backyard. 19

Supplementary Table 9. Distribution of responses across CDR items, in percentages. 19

Supplementary Table 10. Distribution of the three groups as used in the analysis, in percentages. . 21

Supplementary Figure 2. Average marginal component effects of governance and technical features on public support for carbon removal projects (1-10 scale: 1= strongly oppose; 10=strongly support) across six countries and levels of CDR in Backyard. 22

Supplementary Table 11. Estimated average marginal component effects of governance and technical features on public support for carbon removal projects (1-10 scale: 1= strongly oppose; 10=strongly support) across six countries and levels of CDR in Backyard. 22

VI. Figure 3 Corresponding Table 26

Supplementary Table 12. Estimated average marginal component effects with interaction effects between technical attributes of carbon removal projects (1-10 scale: 1= strongly oppose; 10=strongly support) and procedural fairness or distributive fairness across six countries. 26

VII. Figure 4 Additional Items 29

Supplementary Figure 3. Public preferences for fairly engaging local communities in governance of carbon removal projects, across countries (percentages, countries ordered from highest to lowest on "Local population votes"). 29

Supplementary Figure 4. Public preferences for fairly engaging local communities in governance of carbon removal projects, across countries (percentages, countries ordered from highest to lowest on "Local population votes"). 29

VIII. Individual Characteristics 30

Supplementary Table 13. Intra Class Correlation. 30

Supplementary Note 4. Operationalization. 30

Supplementary Table 14. Descriptive statistics. 31

Supplementary Figure 5. Correlation Matrix. 32

Supplementary Table 15. Variance Inflation Factor (VIF). 33

Supplementary Table 16. Estimated average marginal component effects of governance and technical features and selected individual-level characteristics on public support for carbon removal projects (1-10 scale: 1= strongly oppose; 10=strongly support) across six countries. 33

IX. Quotas Information by Country (percentages) – Whole Sample 37

Supplementary Table 17. Descriptive statistics of quota variables by country, in percentages. 37

X. Quotas Information by Country (percentages) – CDR Technologies Sub-Samples 37

Supplementary Table 18. Descriptive statistics of quota variables by country within CDR sub-samples, in percentages. 37

XI. Robustness check excluding respondents selecting the same value across vignettes ... 39

Supplementary Table 19. Count of respondents selecting the same value across all vignette and corresponding values, in percentages. 39

Supplementary Table 20. Estimated average marginal component effects of governance and technical features on public support for carbon removal projects (1-10 scale: 1= strongly oppose; 10=strongly support) across six countries, after excluding respondents who selected the same value across all vignettes. 40

XII. Questions used in the analysis: formulation 41
Supplementary Questionnaire 1..41
XIII. References 52

I. Further information on experimental procedure

Supplementary Note 1. CDR technology and country selection.

The country selection aimed to capture variation in geography, political systems and political economies, while focusing on contexts that share both significant fossil fuel sectors and potential for CDR deployment. Several criteria guided the choice of CDR technologies and countries. Geographic diversity: a mix of European and non-European countries with a total of no more than six countries. Governance: countries represent a diversity of political systems, including liberal democracies, constitutional monarchies, and autocratic regimes, alongside variation in economic systems. Political economy: a mix of fossil fuel regimes and producers and lower-carbon countries. Feasibility and technological potential: each country has potential for at least one CDR method, with a focus on novel ones (BECCS, DACCS, ERW) for which similar levels of public (un)familiarity can be assumed; Novelty: countries that are not already overstudied in the literature (e.g. United States or Germany). Data efficacy: countries where representative data can be collected within our budget and reliably by a professional data collection company.

In all countries, there are emerging projects and initiatives for novel CDR such as DACCS, BECCS, and ERW. The following characterization of each country relies on the State of CDR 2nd Edition unless otherwise stated and focuses on the methods of interest in this study. The statements regarding geological storage potentials are based on Gidden et al. (2025).

Brazil has geological storage potential and extensive biomass resources, creating scope for novel pathways such as BECCS, biochar, and enhanced rock weathering. Currently, there are emerging pilots (e.g., ERW and DACCS). National strategies, including the updated NDC and climate plans acknowledge fossil-linked carbon management.

Italy holds geological storage potential. Current deployment plans are centred on CCS infrastructure such as the Ravenna Hub. Novel CDR approaches, including DACCS and ocean alkalinity enhancement pilots (see e.g. Limenet), remain at early stages (Carbon Gap Policy Tracker). Italy does not have a national carbon management strategy (Global CCS Institute 2025).

Malaysia combines notable geological storage capacity with significant biomass residues, particularly from palm oil production. Current CDR activity is limited to small-scale biochar pilots and discontinued ERW trials (e.g. Cox et al 2025), while policy frameworks emphasize CCUS development and large CCS projects such as Kasawari (see Petronas announcement).

Norway has extensive geological storage resources and established experience with CO_2 storage. It is developing major CCS infrastructure, including Northern Lights, alongside waste-to-energy with CCS and DACCS pilot projects. While multiple CCS facilities are operational or planned, dedicated CDR instruments such as proposals for tax incentives are emerging.

Saudi Arabia possesses large geological storage potential and is piloting DACCS technologies. Its carbon management strategy prioritizes CCS hubs, including the Jubail facility, alongside carbon market development. CDR is framed within the Circular Carbon Economy approach, combining storage, removals, and continued industrial decarbonization efforts.

The United Kingdom has introduced legally binding targets for novel CDR and policy instruments such as carbon contracts for difference. Deployment includes proposed BECCS projects (notably Drax), six DACCS pilots, and 18 small operational CDR projects. At the same

time, large-scale CCUS cluster investments and 39 CCS projects focus primarily on industrial decarbonization and CO_2 storage.

In all six selected countries, CDR pathways are only beginning to emerge; governance arrangements and incentives are nascent. While the EU and the UK have pledged LULUCF targets until 2030, Norway, Brazil, Malaysia, and Saudi Arabia have not quantified removals. Furthermore, none of the countries except the UK has integrated novel removal methods (UK pledge for novel CDR: 5 $MtCO_2$/yr by 2030; Lamb et al. 2025; UNFCC 2025a, b). DACCS and BECCS are covered in the EU Carbon Removal Certification Framework, but they are not yet part of the EU or the UK ETS (Global CCS Institute). Furthermore, the share of novel CDR sold on the voluntary market in 2022/2023 was only 4%. To put this number into perspective: the voluntary market constituted only 2% of total carbon trade (Smith et al. 2024). This means also national policy landscapes and discourse are at best emerging.

Supplementary Note 2. Conceptual background informing the vignette design.

The survey design is rooted in distributional and procedural dimensions of justice. While justice encompasses additional dimensions, including recognition and restorative concerns (Newell et al. 2021; Sovacool et al. 2019), here we focus on procedural and distributive dimensions as they are most directly implicated in implementation processes and particularly salient for the local governance of CDR. Distributional or distributive dimensions of justice concern the way in which costs and benefits, social goods and ills, are allocated across societies and in space and time (Sovacool et al. 2019). For the implementation of CDR, this incites us to ask for example: how are the costs and benefits of implementing a given CDR method distributed across different social groups, geographies and sectors? Distributional dimensions of justice arise alongside procedural dimensions related to mechanisms of decision-making, due process, planning and community engagement as well as ownership (Sovacool et al. 2019). For the implementation of CDR, a procedural justice perspective invites us to ask for example: How are decisions taken, who gets to decide or influence the process and what is perceived to constitute a legitimate and fair process?

Supplementary Table 1. shows how we translated and operationalized these two broad justice dimensions to the vignettes and offers an overview of all attributes included and their possible variations. Two attributes- *decision-making process* and *distribution of profits* - were specifically designed around the procedural and distributive dimensions. Two additional attributes were included in the vignette to contextualize the justice dimensions and allow us to understand interactions between justice-related and other attributes.

Supplementary Table 1. Key elements of the experimental vignette.

Justice Dimension	Attribute	Level 1	Level 2	Level 3
	Implementer	Company	Regional Government	-
Procedural	*Decision-making process*	Authorities with no consultation	Consultation of affected local population	Consultation of scientific experts
Distributive	*Distribution of profits*	Profits to the implementer	Profits redistributed between implementer and affected local population	The project does not generate any profit

	Efficacy	Small - average annual emissions of 5 000 passenger cars	Moderate - average annual emissions of 10 000 passenger cars	High - average annual emissions of 15 000 passenger cars

Prior to full deployment of the survey, we conducted a small-scale pretest of the experimental section of the questionnaire with 156 UK-based respondents in March 2025. The pretest revealed some unclarity in the CDR methods' descriptions, although the vignette assessment yielded expected and satisfactory results. Based on these results, we refined the questionnaire and then proceeded with preregistering the study.

II. Figure 1 Corresponding Tables

Supplementary Table 2. Percentages of responses for support for novel carbon removal technologies (Direct Air Capture (DACCS); Bioenergy with Carbon Capture and Storage (BECCS); Enhanced Rock Weathering (ERW)) across six countries (1= strongly oppose; 10=strongly support).

Country	CDR	1	2	3	4	5	6	7	8	9	10
United Kingdom	DACCS	10.78	4.46	6.19	8.09	16.74	12.98	14.38	11.95	5.46	8.99
	BECCS	6.61	4.44	6.18	7.45	18.83	13.66	13.49	11.62	6.74	10.98
	ERW	6.68	3.37	5.81	7.15	18.8	13.72	14.32	14.89	6.41	8.85
Italy	DACCS	8.6	2.71	4.03	6.21	12.13	14.25	15.4	16.86	7.5	12.3
	BECCS	5.57	3	4.38	5.86	12.32	13.9	14.93	16.41	7.71	15.91
	ERW	5.98	3.21	3.57	5.65	13.09	16.53	15.27	15.8	6.78	14.12
Norway	DACCS	12.56	6.48	9.98	10.2	17.82	12.1	12.1	7.64	3.44	7.7
	BECCS	10.69	4.95	8.61	9.04	18.15	12.67	13.37	10.69	4.06	7.76
	ERW	13.3	5.05	8.02	8.32	18.61	11.85	12.84	9.64	3.99	8.38
Brazil	DACCS	8.09	2.76	3.16	4.73	9.28	7.29	11.25	12.75	9.12	31.58
	BECCS	6.22	2.56	2.7	4.36	9.02	7.49	10.68	12.71	10.6	33.64
	ERW	7.11	2.26	3.32	4.19	8.94	9	11.69	14.35	10.1	29.04
Saudi Arabia	DACCS	6.8	1.76	3.58	4.11	11.01	7.23	10.88	12.21	10.7	31.74
	BECCS	6.03	2.09	2.85	4.3	10.36	7.91	11.89	12.19	10.5	31.89
	ERW	7.38	1.59	3.38	4.21	12.09	8.44	11.46	13.71	8.71	29.04
Malaysia	DACCS	5.43	2.22	3.54	4.24	14.04	11.36	14.54	15.1	9.9	19.64
	BECCS	5.71	2.52	3.75	5.22	13.59	10.43	14.49	16.45	10.6	17.21
	ERW	5.28	2.46	4.22	5.88	13.99	10.27	14.25	15.28	9.87	18.5

Note: N=10,852 respondents. The total for each row is 100%.

Supplementary Table 3. Means of support for novel carbon removal technologies (Direct Air Capture (DACCS); Bioenergy with Carbon Capture and Storage (BECCS); Enhanced Rock Weathering (ERW)) across six countries (1= strongly oppose; 10= strongly support).

Country	CDR	N	Mean	SD	Median	Min	Max
United Kingdom	DACCS	3005	**5.67**	2.63	6	1	10
	BECCS	2995	**5.98**	2.52	6	1	10
	ERW	2995	**6.01**	2.44	6	1	10

Italy	DACCS	3025	**6.3**	2.59	7	1	10
	BECCS	3035	**6.57**	2.52	7	1	10
	ERW	3025	**6.46**	2.48	7	1	10
Norway	DACCS	3025	**5.12**	2.62	5	1	10
	BECCS	3030	**5.43**	2.57	5	1	10
	ERW	3030	**5.32**	2.67	5	1	10
Brazil	DACCS	3005	**7.11**	2.88	8	1	10
	BECCS	3005	**7.35**	2.76	8	1	10
	ERW	3010	**7.15**	2.76	8	1	10
Saudi Arabia	DACCS	3010	**7.24**	2.77	8	1	10
	BECCS	3020	**7.29**	2.71	8	1	10
	ERW	3015	**7.07**	2.76	8	1	10
Malaysia	DACCS	3020	**6.84**	2.53	7	1	10
	BECCS	3005	**6.75**	2.55	7	1	10
	ERW	3005	**6.74**	2.56	7	1	10

Note: N=10,852 respondents. Figure 1 in the main text includes the means, here highlighted in bold.

Supplementary Table 4. Within-countries two-tailed parametric Welch's t-tests with Bonferroni correction applied for multiple comparisons of means of support for novel carbon removal technologies.

Country	CDR Pair	Mean	Mean	t	df	p	Adjusted p
United Kingdom	DACCS-ERW	5.67	6.01	-5.19	5998	0	0
	DACCS-BECCS	5.67	5.98	-4.58	5998	0	0
	BECCS-ERW	5.98	6.01	-0.55	5988	0.58	**1**
Italy	BECCS-ERW	6.57	6.46	1.82	6058	0.07	**0.21**
	DACCS-BECCS	6.3	6.57	-4.14	6058	0	0
	DACCS-ERW	6.3	6.46	-2.38	6048	0.02	0.05
Norway	BECCS-ERW	5.43	5.32	1.63	6058	0.1	**0.31**
	DACCS-ERW	5.12	5.32	-2.94	6053	0	0.01
	DACCS-BECCS	5.12	5.43	-4.64	6053	0	0
Brazil	DACCS-ERW	7.11	7.15	-0.59	6013	0.56	**1**
	DACCS-BECCS	7.11	7.35	-3.39	6008	0	0
	BECCS-ERW	7.35	7.15	2.86	6013	0	0.01
Saudi Arabia	BECCS-ERW	7.29	7.07	3.16	6033	0	0
	DACCS-BECCS	7.24	7.29	-0.71	6028	0.48	**1**
	DACCS-ERW	7.24	7.07	2.42	6023	0.02	**0.05**
Malaysia	DACCS-BECCS	6.84	6.75	1.42	6023	0.16	**0.47**
	BECCS-ERW	6.75	6.74	0.06	6008	0.95	**1**
	DACCS-ERW	6.84	6.74	1.48	6023	0.14	**0.42**

Note. N=10,852 respondents. df= degrees of freedom; Adj. p= Adjusted p-value with Bonferroni correction. Bold highlights the not statistically significant values.

Supplementary Table 5. Between-countries two-tailed parametric Welch's t-tests with Bonferroni correction applied for multiple comparisons of means of support for novel carbon removal technologies.

Country 1	Country 2	CDR	Mean	Mean	t	df	p	Adj. p
United Kingdom	Italy	DACCS	5.67	6.3	-9.38	6025.14	0	0
United Kingdom	Norway	DACCS	5.67	5.12	8.25	6027.34	0	0
United Kingdom	Brazil	DACCS	5.67	7.11	-20.15	5957.52	0	0
United Kingdom	Saudi Arabia	DACCS	5.67	7.24	-22.57	5998.37	0	0
United Kingdom	Malaysia	DACCS	5.67	6.84	-17.51	6012.9	0	0
Italy	Norway	DACCS	6.3	5.12	17.74	6047.22	0	0
Italy	Brazil	DACCS	6.3	7.11	-11.39	5951.12	0	0
Italy	Saudi Arabia	DACCS	6.3	7.24	-13.63	6002.64	0	0
Italy	Malaysia	DACCS	6.3	6.84	-8.11	6040.77	0	0
Norway	Brazil	DACCS	5.12	7.11	-28.08	5965.43	0	0
Norway	Saudi Arabia	DACCS	5.12	7.24	-30.68	6011.51	0	0
Norway	Malaysia	DACCS	5.12	6.84	-25.98	6037.36	0	0
Brazil	Saudi Arabia	DACCS	7.11	7.24	-1.87	6001.98	0.06	**1**
Brazil	Malaysia	DACCS	7.11	6.84	3.85	5918.97	0	0.01
Saudi Arabia	Malaysia	DACCS	7.24	6.84	5.93	5979.36	0	0
United Kingdom	Italy	ERW	6.01	6.46	-7.02	6017.78	0	0
United Kingdom	Norway	ERW	6.01	5.32	10.59	5985.59	0	0
United Kingdom	Brazil	ERW	6.01	7.15	-16.91	5919.07	0	0
United Kingdom	Saudi Arabia	ERW	6.01	7.07	-15.74	5925.68	0	0
United Kingdom	Malaysia	ERW	6.01	6.74	-11.29	5986.24	0	0
Italy	Norway	ERW	6.46	5.32	17.26	6020.86	0	0
Italy	Brazil	ERW	6.46	7.15	-10.24	5956.73	0	0
Italy	Saudi Arabia	ERW	6.46	7.07	-9.07	5963.27	0	0
Italy	Malaysia	ERW	6.46	6.74	-4.36	6019.18	0	0
Norway	Brazil	ERW	5.32	7.15	-26.24	6028.16	0	0
Norway	Saudi Arabia	ERW	5.32	7.07	-25.11	6033.73	0	0
Norway	Malaysia	ERW	5.32	6.74	-21.18	6025.67	0	0
Brazil	Saudi Arabia	ERW	7.15	7.07	1.11	6022.99	0.27	**1**
Brazil	Malaysia	ERW	7.15	6.74	5.96	5979.21	0	0
Saudi Arabia	Malaysia	ERW	7.07	6.74	4.8	5985.26	0	0
United Kingdom	Italy	BECCS	5.98	6.57	-9.19	6026.53	0	0
United Kingdom	Norway	BECCS	5.98	5.43	8.41	6022.63	0	0
United Kingdom	Brazil	BECCS	5.98	7.35	-20.15	5955.52	0	0
United Kingdom	Saudi Arabia	BECCS	5.98	7.29	-19.46	5988.9	0	0
United Kingdom	Malaysia	BECCS	5.98	6.75	-11.71	5997.83	0	0
Italy	Norway	BECCS	6.57	5.43	17.57	6059.66	0	0
Italy	Brazil	BECCS	6.57	7.35	-11.46	5978.21	0	0
Italy	Saudi Arabia	BECCS	6.57	7.29	-10.67	6015.44	0	0
Italy	Malaysia	BECCS	6.57	6.75	-2.61	6035.37	0.01	**0.41**

Norway	Brazil	BECCS	5.43	7.35	-28.07	5997.72	0	0
Norway	Saudi Arabia	BECCS	5.43	7.29	-27.46	6029.34	0	0
Norway	Malaysia	BECCS	5.43	6.75	-20.01	6032.96	0	0
Brazil	Saudi Arabia	BECCS	7.35	7.29	0.86	6020.31	0.39	**1**
Brazil	Malaysia	BECCS	7.35	6.75	8.89	5970.5	0	0
Saudi Arabia	Malaysia	BECCS	7.29	6.75	8.09	6002.66	0	0

Note. N=10,852 respondents. df= degrees of freedom; Adj. p= Adjusted p-value with Bonferroni correction. Bold highlights the not statistically significant values.

III. Vignette Results across CDR Technologies and Countries on Support and Fairness

Supplementary Figure 1. Average marginal component effects of governance and technical features on public support and perception of fairness for carbon removal projects (1-10 scale: 1= strongly oppose; 10=strongly support) across six countries and carbon removal technologies.

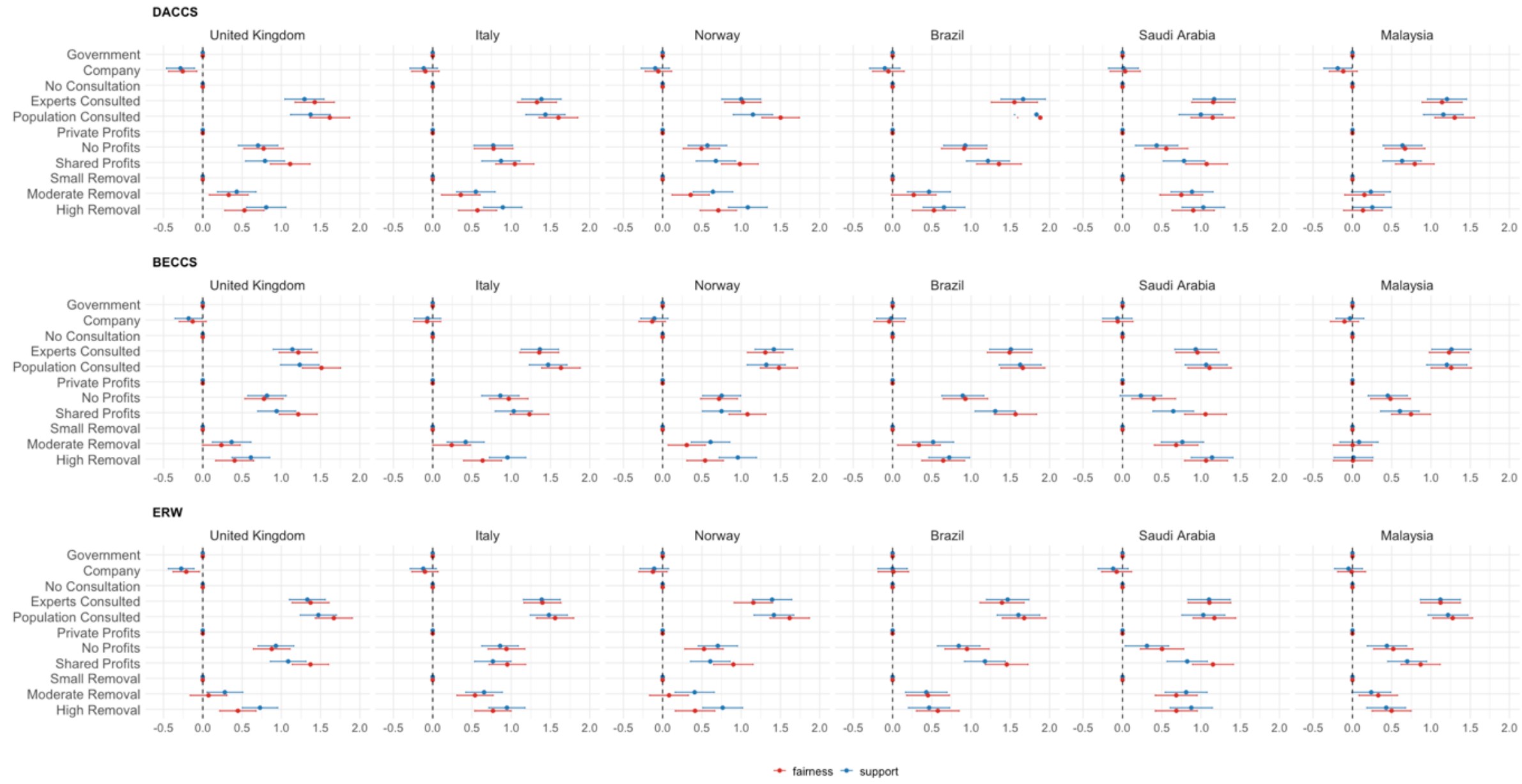

Note: N=10,852 respondents, with a total of 54,260 observations (five per participant). Average Marginal Component Effects (AMCE; unstandardized effect sizes) calculated using ordinary least squares regressions with individual clustered standard errors, one per country, technology and outcome. Y-axis includes the levels of the four socio-technical attributes. X-axis shows AMCEs on the support scale (1 = "strongly oppose" / "very unfair", 10 = "strongly support" / "very fair"). The dashed vertical line highlights the base level 0.0 (i.e. for the base attributes: Government, No Consultation, Private Profits, Small Removal). The mean value of the AMCE is represented by the dots and lines represent the 95% confidence intervals for two-sided t-tests against a zero-null hypothesis. No adjustment for multiple comparisons was applied. Exact P-values and additional regression statistics are reported in Supplementary Table 6.

Supplementary Table 6. Estimated average marginal component effects of governance and technical features on public support for carbon removal projects (1-10 scale: 1= strongly oppose; 10=strongly support) across six countries and carbon removal technologies.

**Outcome= Support**											
**DACCS**											
**Country**	**Attribute**	**Est.**	**s.e.**	**t-stat.**	**p-val.**	**stars**	**conf.low**	**conf.high**	**N of Vig.**	**N of Resp.**	**r2**
United Kingdom	Constant	4,02	0,132	30,409	0	***	3,765	4,275	3005	601	0,093
	Company	-0,284	0,091	-3,101	0,002	**	-0,463	-0,104	3005	601	0,093

	Experts Consulted	1,295	0,112	11,528	0	***	1,076	1,514	3005	601	0,093
	Locals Consulted	1,372	0,113	12,099	0	***	1,153	1,591	3005	601	0,093
	Shared Profits	0,792	0,112	7,095	0	***	0,572	1,011	3005	601	0,093
	No Profits	0,704	0,113	6,203	0	***	0,484	0,923	3005	601	0,093
	Medium Removal	0,433	0,111	3,916	0	***	0,214	0,652	3005	601	0,093
	High Removal	0,808	0,113	7,134	0	***	0,587	1,028	3005	601	0,093
Italy	Constant	4,387	0,133	32,937	0	***	4,14	4,634	3025	605	0,11
	Company	-0,114	0,089	-1,287	0,198		-0,289	0,06	3025	605	0,11
	Experts Consulted	1,385	0,113	12,306	0	***	1,172	1,599	3025	605	0,11
	Locals Consulted	1,435	0,112	12,83	0	***	1,221	1,649	3025	605	0,11
	Shared Profits	0,869	0,109	7,98	0	***	0,657	1,082	3025	605	0,11
	No Profits	0,771	0,11	6,989	0	***	0,557	0,986	3025	605	0,11
	Medium Removal	0,55	0,11	4,991	0	***	0,335	0,765	3025	605	0,11
	High Removal	0,893	0,11	8,135	0	***	0,68	1,106	3025	605	0,11
Norway	Constant	3,456	0,127	27,255	0	***	3,204	3,708	3025	605	0,082
	Company	-0,094	0,091	-1,027	0,305		-0,273	0,085	3025	605	0,082
	Experts Consulted	1,001	0,112	8,978	0	***	0,782	1,221	3025	605	0,082
	Locals Consulted	1,15	0,112	10,247	0	***	0,931	1,37	3025	605	0,082
	Shared Profits	0,678	0,112	6,04	0	***	0,459	0,897	3025	605	0,082
	No Profits	0,571	0,111	5,153	0	***	0,351	0,79	3025	605	0,082
	Medium Removal	0,641	0,113	5,66	0	***	0,422	0,859	3025	605	0,082
	High Removal	1,085	0,111	9,769	0	***	0,865	1,304	3025	605	0,082
Brazil	Constant	4,917	0,153	32,237	0	***	4,647	5,187	3005	601	0,127
	Company	-0,099	0,098	-1,004	0,315		-0,292	0,094	3005	601	0,127
	Experts Consulted	1,663	0,127	13,113	0	***	1,428	1,899	3005	601	0,127
	Locals Consulted	1,833	0,126	14,595	0	***	1,597	2,07	3005	601	0,127
	Shared Profits	1,215	0,123	9,89	0	***	0,978	1,451	3005	601	0,127
	No Profits	0,926	0,124	7,482	0	***	0,69	1,162	3005	601	0,127
	Medium Removal	0,464	0,123	3,77	0	***	0,227	0,701	3005	601	0,127
	High Removal	0,655	0,12	5,467	0	***	0,421	0,889	3005	601	0,127
Saudi Arabia	Constant	5,469	0,148	36,904	0	***	5,196	5,741	3010	602	0,074
	Company	0,012	0,097	0,126	0,9		-0,178	0,203	3010	602	0,074
	Experts Consulted	1,168	0,121	9,645	0	***	0,935	1,401	3010	602	0,074
	Locals Consulted	0,998	0,125	8,016	0	***	0,765	1,231	3010	602	0,074
	Shared Profits	0,784	0,119	6,573	0	***	0,549	1,018	3010	602	0,074
	No Profits	0,435	0,121	3,593	0	***	0,203	0,668	3010	602	0,074
	Medium Removal	0,885	0,12	7,353	0	***	0,652	1,119	3010	602	0,074
	High Removal	1,03	0,122	8,475	0	***	0,796	1,263	3010	602	0,074
Malaysia	Constant	5,557	0,139	40,045	0	***	5,307	5,807	3020	604	0,066
	Company	-0,187	0,089	-2,094	0,036	*	-0,362	-0,012	3020	604	0,066

Country	Attribute	Est.	s.e.	t-stat.	p-val.	stars	conf.low	conf.high	N of Vig.	N of Resp.	r2
	Experts Consulted	1,204	0,113	10,696	0	***	0,989	1,419	3020	604	0,066
	Locals Consulted	1,158	0,113	10,216	0	***	0,944	1,372	3020	604	0,066
	Shared Profits	0,633	0,11	5,76	0	***	0,418	0,848	3020	604	0,066
	No Profits	0,639	0,111	5,743	0	***	0,425	0,852	3020	604	0,066
	Medium Removal	0,234	0,112	2,097	0,036	*	0,015	0,453	3020	604	0,066
	High Removal	0,253	0,11	2,311	0,021	*	0,041	0,466	3020	604	0,066
**BECCS**											
**Country**	**Attribute**	**Est.**	**s.e.**	**t-stat.**	**p-val.**	**stars**	**conf.low**	**conf.high**	**N of Vig.**	**N of Resp.**	**r2**
United Kingdom	Constant	4,354	0,13	33,582	0	***	4,111	4,598	2995	599	0,09
	Company	-0,18	0,088	-2,046	0,041	*	-0,353	-0,008	2995	599	0,09
	Experts Consulted	1,142	0,11	10,359	0	***	0,93	1,353	2995	599	0,09
	Locals Consulted	1,235	0,11	11,271	0	***	1,023	1,448	2995	599	0,09
	Shared Profits	0,941	0,108	8,672	0	***	0,729	1,152	2995	599	0,09
	No Profits	0,818	0,11	7,444	0	***	0,606	1,03	2995	599	0,09
	Medium Removal	0,367	0,11	3,34	0,001	***	0,154	0,58	2995	599	0,09
	High Removal	0,613	0,108	5,666	0	***	0,401	0,824	2995	599	0,09
Italy	Constant	4,586	0,128	35,748	0	***	4,349	4,824	3035	607	0,125
	Company	-0,066	0,086	-0,769	0,442		-0,234	0,102	3035	607	0,125
	Experts Consulted	1,365	0,107	12,707	0	***	1,16	1,571	3035	607	0,125
	Locals Consulted	1,469	0,108	13,656	0	***	1,264	1,675	3035	607	0,125
	Shared Profits	1,032	0,106	9,721	0	***	0,826	1,238	3035	607	0,125
	No Profits	0,861	0,107	8,086	0	***	0,656	1,067	3035	607	0,125
	Medium Removal	0,42	0,106	3,971	0	***	0,216	0,624	3035	607	0,125
	High Removal	0,953	0,104	9,137	0	***	0,746	1,16	3035	607	0,125
Norway	Constant	3,544	0,127	27,933	0	***	3,295	3,793	3030	606	0,104
	Company	-0,105	0,089	-1,19	0,234		-0,279	0,068	3030	606	0,104
	Experts Consulted	1,416	0,108	13,138	0	***	1,203	1,629	3030	606	0,104
	Locals Consulted	1,32	0,109	12,068	0	***	1,108	1,532	3030	606	0,104
	Shared Profits	0,75	0,109	6,849	0	***	0,536	0,963	3030	606	0,104
	No Profits	0,752	0,109	6,9	0	***	0,539	0,965	3030	606	0,104
	Medium Removal	0,611	0,11	5,557	0	***	0,397	0,825	3030	606	0,104
	High Removal	0,958	0,107	8,923	0	***	0,745	1,17	3030	606	0,104
Brazil	Constant	5,173	0,147	35,114	0	***	4,91	5,436	3005	601	0,123
	Company	-0,018	0,094	-0,195	0,845		-0,203	0,167	3005	601	0,123
	Experts Consulted	1,507	0,122	12,365	0	***	1,28	1,734	3005	601	0,123
	Locals Consulted	1,625	0,12	13,512	0	***	1,399	1,851	3005	601	0,123
	Shared Profits	1,308	0,115	11,344	0	***	1,082	1,533	3005	601	0,123
	No Profits	0,894	0,12	7,426	0	***	0,667	1,121	3005	601	0,123
	Medium Removal	0,517	0,117	4,422	0	***	0,29	0,743	3005	601	0,123
	High Removal	0,722	0,117	6,164	0	***	0,494	0,95	3005	601	0,123

Saudi Arabia	Constant	5,73	0,144	39,705	0	***	5,469	5,991	3020	604	0,072
	Company	-0,066	0,095	-0,689	0,491		-0,252	0,121	3020	604	0,072
	Experts Consulted	0,933	0,12	7,757	0	***	0,704	1,161	3020	604	0,072
	Locals Consulted	1,066	0,12	8,873	0	***	0,837	1,294	3020	604	0,072
	Shared Profits	0,647	0,116	5,554	0	***	0,418	0,875	3020	604	0,072
	No Profits	0,234	0,119	1,968	0,049	*	0,006	0,463	3020	604	0,072
	Medium Removal	0,763	0,121	6,308	0	***	0,534	0,992	3020	604	0,072
	High Removal	1,141	0,119	9,549	0	***	0,912	1,37	3020	604	0,072
Malaysia	Constant	5,561	0,132	41,972	0	***	5,313	5,809	3005	601	0,061
	Company	-0,032	0,09	-0,354	0,723		-0,209	0,145	3005	601	0,061
	Experts Consulted	1,262	0,113	11,209	0	***	1,046	1,477	3005	601	0,061
	Locals Consulted	1,203	0,115	10,464	0	***	0,986	1,42	3005	601	0,061
	Shared Profits	0,605	0,11	5,482	0	***	0,388	0,822	3005	601	0,061
	No Profits	0,45	0,112	4,01	0	***	0,234	0,667	3005	601	0,061
	Medium Removal	0,084	0,109	0,769	0,442		-0,131	0,299	3005	601	0,061
	High Removal	0,014	0,111	0,126	0,899		-0,203	0,231	3005	601	0,061

ERW

Country	Attribute	Est.	s.e.	t-stat.	p-val.	stars	conf.low	conf.high	N of Vig.	N of Resp.	r2
United Kingdom	Constant	4,213	0,121	34,957	0	***	3,984	4,443	2995	599	0,131
	Company	-0,275	0,083	-3,305	0,001	***	-0,438	-0,111	2995	599	0,131
	Experts Consulted	1,332	0,103	12,971	0	***	1,132	1,532	2995	599	0,131
	Locals Consulted	1,472	0,104	14,201	0	***	1,273	1,672	2995	599	0,131
	Shared Profits	1,088	0,102	10,661	0	***	0,889	1,287	2995	599	0,131
	No Profits	0,932	0,102	9,113	0	***	0,732	1,131	2995	599	0,131
	Medium Removal	0,282	0,102	2,75	0,006	**	0,083	0,481	2995	599	0,131
	High Removal	0,729	0,101	7,194	0	***	0,53	0,928	2995	599	0,131
Italy	Constant	4,485	0,126	35,669	0	***	4,253	4,717	3025	605	0,126
	Company	-0,12	0,084	-1,422	0,155		-0,285	0,045	3025	605	0,126
	Experts Consulted	1,389	0,106	13,152	0	***	1,186	1,592	3025	605	0,126
	Locals Consulted	1,48	0,106	13,934	0	***	1,277	1,682	3025	605	0,126
	Shared Profits	0,766	0,104	7,367	0	***	0,563	0,97	3025	605	0,126
	No Profits	0,858	0,104	8,216	0	***	0,656	1,06	3025	605	0,126
	Medium Removal	0,654	0,104	6,266	0	***	0,452	0,857	3025	605	0,126
	High Removal	0,945	0,104	9,103	0	***	0,743	1,147	3025	605	0,126
Norway	Constant	3,604	0,127	28,476	0	***	3,348	3,859	3030	606	0,09
	Company	-0,107	0,093	-1,152	0,249		-0,288	0,075	3030	606	0,09
	Experts Consulted	1,394	0,112	12,409	0	***	1,172	1,617	3030	606	0,09
	Locals Consulted	1,417	0,114	12,449	0	***	1,193	1,64	3030	606	0,09
	Shared Profits	0,607	0,113	5,353	0	***	0,385	0,829	3030	606	0,09
	No Profits	0,704	0,112	6,261	0	***	0,482	0,925	3030	606	0,09

	Medium Removal	0,408	0,112	3,643	0	***	0,186	0,63	3030	606	0,09
	High Removal	0,765	0,114	6,702	0	***	0,542	0,988	3030	606	0,09
Brazil	Constant	5,142	0,15	34,369	0	***	4,875	5,409	3010	602	0,105
	Company	0	0,095	-0,002	0,999		-0,187	0,187	3010	602	0,105
	Experts Consulted	1,466	0,122	12,033	0	***	1,237	1,695	3010	602	0,105
	Locals Consulted	1,603	0,122	13,117	0	***	1,374	1,833	3010	602	0,105
	Shared Profits	1,176	0,117	10,061	0	***	0,946	1,406	3010	602	0,105
	No Profits	0,843	0,122	6,925	0	***	0,614	1,072	3010	602	0,105
	Medium Removal	0,43	0,119	3,621	0	***	0,201	0,659	3010	602	0,105
	High Removal	0,465	0,118	3,938	0	***	0,235	0,695	3010	602	0,105
Saudi Arabia	Constant	5,479	0,146	37,545	0	***	5,21	5,749	3015	603	0,068
	Company	-0,119	0,097	-1,224	0,221		-0,31	0,072	3015	603	0,068
	Experts Consulted	1,102	0,122	9,072	0	***	0,868	1,336	3015	603	0,068
	Locals Consulted	1,03	0,122	8,407	0	***	0,796	1,263	3015	603	0,068
	Shared Profits	0,824	0,116	7,102	0	***	0,592	1,056	3015	603	0,068
	No Profits	0,312	0,125	2,502	0,012	*	0,077	0,546	3015	603	0,068
	Medium Removal	0,811	0,12	6,752	0	***	0,578	1,044	3015	603	0,068
	High Removal	0,876	0,121	7,241	0	***	0,643	1,109	3015	603	0,068
Malaysia	Constant	5,379	0,137	39,283	0	***	5,128	5,631	3005	601	0,065
	Company	-0,05	0,09	-0,551	0,582		-0,227	0,128	3005	601	0,065
	Experts Consulted	1,12	0,115	9,752	0	***	0,903	1,338	3005	601	0,065
	Locals Consulted	1,217	0,114	10,65	0	***	1	1,435	3005	601	0,065
	Shared Profits	0,698	0,111	6,275	0	***	0,481	0,914	3005	601	0,065
	No Profits	0,439	0,113	3,901	0	***	0,221	0,658	3005	601	0,065
	Medium Removal	0,24	0,111	2,154	0,031	*	0,025	0,454	3005	601	0,065
	High Removal	0,43	0,111	3,871	0	***	0,21	0,65	3005	601	0,065

Note: Estimates from OLS regressions with clustered standard errors at the individual level. Reference levels: regional government, no consultation, private profits, small removal. Statistical inference is based on two-sided t-tests against a zero-null hypothesis. No adjustment for multiple comparisons was applied. Est. = estimates; s.e. = standard errors; t-stat. = t-statistics; p-val. = p-values; stars = "***" ~ p < 0.001, "**" ~ p < 0.01, "*" ~ p < 0.05; conf.low and conf.high delimit the confidence intervals; N of Vig. = number of vignettes (technology and country-level); N of Resp. = number of respondents (technology and country-level); r2 = r squared.

Supplementary Table 7. Estimated average marginal component effects of governance and technical features on public perception of fairness for carbon removal projects (1-10 scale: 1= very unfair; 10=very fair) across six countries and carbon removal technologies.

**Outcome= Fairness**											
**DACCS**											
**Country**	**Attribute**	**Est.**	**s.e.**	**t-stat.**	**p-val.**	**stars**	**conf.low**	**conf.high**	**N of Vig.**	**N of Resp.**	**r2**
United Kingdom	Constant	3,514	0,132	26,582	0	***	3,257	3,77	3005	601	0,111
	Company	-0,255	0,092	-2,776	0,006	**	-0,435	-0,075	3005	601	0,111
	Experts Consulted	1,425	0,112	12,672	0	***	1,205	1,646	3005	601	0,111
	Locals Consulted	1,618	0,114	14,202	0	***	1,398	1,838	3005	601	0,111

	Shared Profits	1,113	0,113	9,85	0	***	0,893	1,334	3005	601	0,111
	No Profits	0,775	0,113	6,837	0	***	0,554	0,996	3005	601	0,111
	Medium Removal	0,331	0,111	2,979	0,003	**	0,11	0,551	3005	601	0,111
	High Removal	0,531	0,112	4,724	0	***	0,309	0,752	3005	601	0,111
Italy	Constant	4,177	0,133	31,405	0	***	3,929	4,424	3025	605	0,112
	Company	-0,093	0,089	-1,045	0,296		-0,267	0,081	3025	605	0,112
	Experts Consulted	1,328	0,112	11,874	0	***	1,115	1,542	3025	605	0,112
	Locals Consulted	1,601	0,111	14,431	0	***	1,388	1,815	3025	605	0,112
	Shared Profits	1,046	0,109	9,589	0	***	0,833	1,259	3025	605	0,112
	No Profits	0,775	0,111	6,983	0	***	0,561	0,989	3025	605	0,112
	Medium Removal	0,358	0,11	3,254	0,001	**	0,143	0,573	3025	605	0,112
	High Removal	0,571	0,109	5,218	0	***	0,358	0,783	3025	605	0,112
Norway	Constant	3,094	0,12	25,817	0	***	2,857	3,331	3025	605	0,104
	Company	-0,054	0,086	-0,634	0,526		-0,223	0,114	3025	605	0,104
	Experts Consulted	1,021	0,104	9,782	0	***	0,815	1,228	3025	605	0,104
	Locals Consulted	1,503	0,107	14,058	0	***	1,297	1,709	3025	605	0,104
	Shared Profits	0,983	0,105	9,406	0	***	0,777	1,189	3025	605	0,104
	No Profits	0,495	0,105	4,723	0	***	0,288	0,701	3025	605	0,104
	Medium Removal	0,357	0,107	3,347	0,001	***	0,152	0,563	3025	605	0,104
	High Removal	0,709	0,104	6,806	0	***	0,503	0,915	3025	605	0,104
Brazil	Constant	4,664	0,156	29,891	0	***	4,382	4,946	3005	601	0,118
	Company	-0,053	0,103	-0,513	0,608		-0,255	0,149	3005	601	0,118
	Experts Consulted	1,552	0,133	11,706	0	***	1,306	1,798	3005	601	0,118
	Locals Consulted	1,881	0,129	14,565	0	***	1,634	2,129	3005	601	0,118
	Shared Profits	1,355	0,128	10,597	0	***	1,108	1,602	3005	601	0,118
	No Profits	0,911	0,13	7,021	0	***	0,665	1,158	3005	601	0,118
	Medium Removal	0,269	0,128	2,107	0,035	*	0,022	0,517	3005	601	0,118
	High Removal	0,527	0,124	4,256	0	***	0,282	0,771	3005	601	0,118
Saudi Arabia	Constant	5,122	0,149	34,481	0	***	4,846	5,397	3010	602	0,08
	Company	0,034	0,098	0,348	0,728		-0,158	0,227	3010	602	0,08
	Experts Consulted	1,155	0,123	9,37	0	***	0,919	1,391	3010	602	0,08
	Locals Consulted	1,149	0,124	9,251	0	***	0,912	1,385	3010	602	0,08
	Shared Profits	1,071	0,12	8,929	0	***	0,835	1,308	3010	602	0,08
	No Profits	0,557	0,123	4,514	0	***	0,321	0,792	3010	602	0,08
	Medium Removal	0,75	0,122	6,126	0	***	0,514	0,986	3010	602	0,08
	High Removal	0,901	0,121	7,432	0	***	0,665	1,137	3010	602	0,08
Malaysia	Constant	5,047	0,138	36,505	0	***	4,793	5,301	3020	604	0,07
	Company	-0,116	0,091	-1,282	0,2		-0,294	0,062	3020	604	0,07
	Experts Consulted	1,142	0,115	9,958	0	***	0,923	1,361	3020	604	0,07
	Locals Consulted	1,302	0,113	11,49	0	***	1,085	1,519	3020	604	0,07

	Shared Profits	0,794	0,111	7,169	0	***	0,576	1,013	3020	604	0,07
	No Profits	0,671	0,114	5,905	0	***	0,454	0,887	3020	604	0,07
	Medium Removal	0,153	0,113	1,358	0,175		-0,07	0,376	3020	604	0,07
	High Removal	0,134	0,111	1,212	0,226		-0,081	0,35	3020	604	0,07

BECCS

Country	Attribute	Est.	s.e.	t-stat.	p-val.	stars	conf.low	conf.high	N of Vig.	N of Resp.	r2
United Kingdom	Constant	3,833	0,128	29,935	0	***	3,589	4,076	2995	599	0,11
	Company	-0,128	0,088	-1,453	0,146		-0,301	0,045	2995	599	0,11
	Experts Consulted	1,217	0,11	11,031	0	***	1,005	1,428	2995	599	0,11
	Locals Consulted	1,512	0,11	13,762	0	***	1,299	1,725	2995	599	0,11
	Shared Profits	1,216	0,108	11,214	0	***	1,004	1,427	2995	599	0,11
	No Profits	0,78	0,11	7,115	0	***	0,567	0,992	2995	599	0,11
	Medium Removal	0,237	0,108	2,196	0,028	*	0,024	0,451	2995	599	0,11
	High Removal	0,406	0,109	3,714	0	***	0,194	0,618	2995	599	0,11
Italy	Constant	4,276	0,132	32,394	0	***	4,029	4,523	3035	607	0,124
	Company	-0,072	0,089	-0,808	0,419		-0,246	0,102	3035	607	0,124
	Experts Consulted	1,357	0,112	12,139	0	***	1,144	1,57	3035	607	0,124
	Locals Consulted	1,633	0,11	14,811	0	***	1,42	1,847	3035	607	0,124
	Shared Profits	1,232	0,11	11,157	0	***	1,019	1,446	3035	607	0,124
	No Profits	0,968	0,11	8,799	0	***	0,755	1,182	3035	607	0,124
	Medium Removal	0,242	0,108	2,231	0,026	*	0,03	0,454	3035	607	0,124
	High Removal	0,635	0,109	5,823	0	***	0,42	0,85	3035	607	0,124
Norway	Constant	3,295	0,123	26,875	0	***	3,053	3,536	3030	606	0,11
	Company	-0,132	0,086	-1,536	0,125		-0,301	0,037	3030	606	0,11
	Experts Consulted	1,308	0,103	12,689	0	***	1,101	1,515	3030	606	0,11
	Locals Consulted	1,482	0,107	13,911	0	***	1,276	1,688	3030	606	0,11
	Shared Profits	1,08	0,106	10,209	0	***	0,872	1,288	3030	606	0,11
	No Profits	0,719	0,105	6,831	0	***	0,512	0,926	3030	606	0,11
	Medium Removal	0,307	0,107	2,882	0,004	**	0,099	0,515	3030	606	0,11
	High Removal	0,541	0,105	5,178	0	***	0,335	0,748	3030	606	0,11
Brazil	Constant	4,826	0,151	31,969	0	***	4,549	5,103	3005	601	0,123
	Company	-0,043	0,099	-0,429	0,668		-0,237	0,152	3005	601	0,123
	Experts Consulted	1,491	0,128	11,677	0	***	1,252	1,73	3005	601	0,123
	Locals Consulted	1,659	0,126	13,217	0	***	1,422	1,897	3005	601	0,123
	Shared Profits	1,566	0,12	13,004	0	***	1,329	1,803	3005	601	0,123
	No Profits	0,928	0,128	7,269	0	***	0,689	1,167	3005	601	0,123
	Medium Removal	0,336	0,123	2,739	0,006	**	0,097	0,574	3005	601	0,123
	High Removal	0,645	0,123	5,266	0	***	0,405	0,885	3005	601	0,123
Saudi Arabia	Constant	5,32	0,15	35,511	0	***	5,049	5,591	3020	604	0,08
	Company	-0,06	0,099	-0,608	0,543		-0,254	0,134	3020	604	0,08

	Experts Consulted	0,955	0,124	7,708	0	***	0,718	1,192	3020	604	0,08
	Locals Consulted	1,109	0,124	8,928	0	***	0,872	1,346	3020	604	0,08
	Shared Profits	1,058	0,12	8,812	0	***	0,821	1,295	3020	604	0,08
	No Profits	0,398	0,126	3,167	0,002	**	0,16	0,635	3020	604	0,08
	Medium Removal	0,683	0,125	5,479	0	***	0,446	0,921	3020	604	0,08
	High Removal	1,064	0,123	8,635	0	***	0,827	1,302	3020	604	0,08
Malaysia	Constant	5,116	0,131	39,05	0	***	4,864	5,367	3005	601	0,065
	Company	-0,101	0,091	-1,104	0,27		-0,28	0,078	3005	601	0,065
	Experts Consulted	1,231	0,113	10,853	0	***	1,013	1,45	3005	601	0,065
	Locals Consulted	1,259	0,115	10,945	0	***	1,04	1,479	3005	601	0,065
	Shared Profits	0,746	0,111	6,697	0	***	0,527	0,966	3005	601	0,065
	No Profits	0,485	0,114	4,252	0	***	0,266	0,704	3005	601	0,065
	Medium Removal	0,003	0,111	0,025	0,98		-0,215	0,22	3005	601	0,065
	High Removal	0,006	0,111	0,052	0,958		-0,214	0,225	3005	601	0,065

ERW

Country	Attribute	Est.	s.e.	t-stat.	p-val.	stars	conf.low	conf.high	N of Vig.	N of Resp.	r2
United Kingdom	Constant	3,752	0,122	30,789	0	***	3,516	3,989	2995	599	0,14
	Company	-0,211	0,086	-2,461	0,014	*	-0,379	-0,043	2995	599	0,14
	Experts Consulted	1,373	0,106	12,973	0	***	1,167	1,578	2995	599	0,14
	Locals Consulted	1,669	0,106	15,764	0	***	1,464	1,874	2995	599	0,14
	Shared Profits	1,372	0,104	13,161	0	***	1,166	1,577	2995	599	0,14
	No Profits	0,879	0,105	8,346	0	***	0,673	1,084	2995	599	0,14
	Medium Removal	0,074	0,105	0,702	0,483		-0,131	0,279	2995	599	0,14
	High Removal	0,448	0,104	4,303	0	***	0,242	0,653	2995	599	0,14
Italy	Constant	4,161	0,124	33,637	0	***	3,928	4,395	3025	605	0,129
	Company	-0,099	0,085	-1,159	0,246		-0,265	0,068	3025	605	0,129
	Experts Consulted	1,397	0,106	13,14	0	***	1,193	1,601	3025	605	0,129
	Locals Consulted	1,559	0,106	14,717	0	***	1,356	1,763	3025	605	0,129
	Shared Profits	0,95	0,105	9,072	0	***	0,746	1,155	3025	605	0,129
	No Profits	0,94	0,105	8,929	0	***	0,736	1,143	3025	605	0,129
	Medium Removal	0,539	0,104	5,2	0	***	0,335	0,743	3025	605	0,129
	High Removal	0,768	0,104	7,38	0	***	0,564	0,971	3025	605	0,129
Norway	Constant	3,391	0,129	26,372	0	***	3,139	3,644	3030	606	0,091
	Company	-0,123	0,092	-1,346	0,178		-0,303	0,056	3030	606	0,091
	Experts Consulted	1,156	0,11	10,46	0	***	0,936	1,375	3030	606	0,091
	Locals Consulted	1,618	0,113	14,312	0	***	1,397	1,839	3030	606	0,091
	Shared Profits	0,9	0,113	7,953	0	***	0,681	1,12	3030	606	0,091
	No Profits	0,527	0,11	4,779	0	***	0,308	0,746	3030	606	0,091
	Medium Removal	0,082	0,111	0,734	0,463		-0,138	0,301	3030	606	0,091
	High Removal	0,413	0,113	3,641	0	***	0,192	0,633	3030	606	0,091

Brazil	Constant	4,648	0,153	30,431	0	***	4,371	4,925	3010	602	0,113
	Company	0,011	0,099	0,106	0,915		-0,184	0,205	3010	602	0,113
	Experts Consulted	1,394	0,126	11,021	0	***	1,155	1,632	3010	602	0,113
	Locals Consulted	1,676	0,125	13,406	0	***	1,438	1,915	3010	602	0,113
	Shared Profits	1,454	0,122	11,897	0	***	1,215	1,693	3010	602	0,113
	No Profits	0,948	0,127	7,489	0	***	0,71	1,187	3010	602	0,113
	Medium Removal	0,451	0,123	3,675	0	***	0,214	0,689	3010	602	0,113
	High Removal	0,576	0,122	4,706	0	***	0,337	0,815	3010	602	0,113
Saudi Arabia	Constant	5,107	0,145	35,152	0	***	4,838	5,376	3015	603	0,08
	Company	-0,074	0,097	-0,766	0,444		-0,265	0,116	3015	603	0,08
	Experts Consulted	1,107	0,122	9,057	0	***	0,874	1,341	3015	603	0,08
	Locals Consulted	1,17	0,122	9,611	0	***	0,938	1,403	3015	603	0,08
	Shared Profits	1,153	0,117	9,84	0	***	0,921	1,385	3015	603	0,08
	No Profits	0,504	0,125	4,047	0	***	0,27	0,738	3015	603	0,08
	Medium Removal	0,685	0,12	5,715	0	***	0,452	0,918	3015	603	0,08
	High Removal	0,686	0,12	5,71	0	***	0,453	0,918	3015	603	0,08
Malaysia	Constant	4,684	0,134	34,855	0	***	4,433	4,935	3005	601	0,076
	Company	-0,01	0,09	-0,114	0,909		-0,187	0,167	3005	601	0,076
	Experts Consulted	1,123	0,114	9,887	0	***	0,906	1,34	3005	601	0,076
	Locals Consulted	1,277	0,113	11,259	0	***	1,06	1,494	3005	601	0,076
	Shared Profits	0,869	0,111	7,797	0	***	0,653	1,085	3005	601	0,076
	No Profits	0,52	0,112	4,625	0	***	0,302	0,738	3005	601	0,076
	Medium Removal	0,329	0,111	2,982	0,003	**	0,115	0,544	3005	601	0,076
	High Removal	0,499	0,112	4,473	0	***	0,28	0,719	3005	601	0,076

Note: Estimates from OLS regressions with clustered standard errors at the individual level (in parentheses). Reference levels: regional government, no consultation, private profits, small removal. Statistical inference is based on two-sided t-tests against a zero-null hypothesis. No adjustment for multiple comparisons was applied. Est. = estimates; s.e. = standard errors; t-stat. = t-statistics; p-val. = p-values; stars = "***" ~ $p < 0.001$, "**" ~ $p < 0.01$, "*" ~ $p < 0.05$; conf.low and conf.high delimit the confidence intervals; N of Vig. = number of vignettes (technology and country-level); N of Resp. = number of respondents (technology and country-level); r2 = r squared.

IV. Figure 2 Corresponding Table

Supplementary Table 8. Estimated average marginal component effects of governance and technical features on public support for carbon removal projects (1-10 scale: 1= strongly oppose; 10=strongly support) across six countries.

Country	Attributes	Est.	s.e.	t-stat.	p-val.	stars	conf.low	conf.high	N of Vig.	N of Resp.	r2
United Kingdom	Constant	4,197	0,082	50,873	0	***	4,057	4,337	8995	1799	0,102
	Company	-0,247	0,036	-6,928	0	***	-0,347	-0,148	8995	1799	0,102
	Experts Consulted	1,258	0,052	24,042	0	***	1,136	1,379	8995	1799	0,102
	Locals Consulted	1,361	0,054	25,389	0	***	1,239	1,482	8995	1799	0,102
	Shared Profits	0,939	0,05	18,96	0	***	0,817	1,06	8995	1799	0,102

	No Profits	0,815	0,051	15,942	0	***	0,693	0,936	8995	1799	0,102
	Medium Removal	0,36	0,067	5,381	0	***	0,238	0,481	8995	1799	0,102
	High Removal	0,716	0,067	10,745	0	***	0,594	0,837	8995	1799	0,102
Italy	Constant	4,488	0,081	55,557	0	***	4,35	4,626	9085	1817	0,119
	Company	-0,101	0,037	-2,75	0,006	**	-0,198	-0,003	9085	1817	0,119
	Experts Consulted	1,375	0,056	24,649	0	***	1,255	1,495	9085	1817	0,119
	Locals Consulted	1,458	0,055	26,549	0	***	1,339	1,578	9085	1817	0,119
	Shared Profits	0,89	0,051	17,494	0	***	0,77	1,01	9085	1817	0,119
	No Profits	0,835	0,051	16,4	0	***	0,715	0,954	9085	1817	0,119
	Medium Removal	0,541	0,064	8,505	0	***	0,421	0,661	9085	1817	0,119
	High Removal	0,926	0,065	14,201	0	***	0,807	1,046	9085	1817	0,119
Norway	Constant	3,535	0,079	44,881	0	***	3,389	3,68	9085	1817	0,09
	Company	-0,106	0,039	-2,741	0,006	**	-0,209	-0,003	9085	1817	0,09
	Experts Consulted	1,27	0,052	24,59	0	***	1,144	1,396	9085	1817	0,09
	Locals Consulted	1,295	0,052	24,686	0	***	1,169	1,421	9085	1817	0,09
	Shared Profits	0,68	0,049	13,779	0	***	0,554	0,806	9085	1817	0,09
	No Profits	0,68	0,048	14,119	0	***	0,554	0,806	9085	1817	0,09
	Medium Removal	0,556	0,067	8,27	0	***	0,43	0,682	9085	1817	0,09
	High Removal	0,934	0,069	13,529	0	***	0,808	1,06	9085	1817	0,09
Brazil	Constant	5,073	0,098	51,784	0	***	4,919	5,226	9020	1804	0,118
	Company	-0,036	0,042	-0,848	0,397		-0,144	0,073	9020	1804	0,118
	Experts Consulted	1,546	0,066	23,581	0	***	1,413	1,679	9020	1804	0,118
	Locals Consulted	1,688	0,066	25,388	0	***	1,555	1,821	9020	1804	0,118
	Shared Profits	1,235	0,061	20,368	0	***	1,102	1,368	9020	1804	0,118
	No Profits	0,886	0,06	14,815	0	***	0,752	1,019	9020	1804	0,118
	Medium Removal	0,475	0,073	6,51	0	***	0,342	0,608	9020	1804	0,118
	High Removal	0,618	0,073	8,424	0	***	0,485	0,751	9020	1804	0,118
Saudi Arabia	Constant	5,563	0,096	58,047	0	***	5,408	5,717	9045	1809	0,07
	Company	-0,058	0,044	-1,317	0,188		-0,167	0,051	9045	1809	0,07
	Experts Consulted	1,067	0,062	17,275	0	***	0,934	1,201	9045	1809	0,07
	Locals Consulted	1,03	0,062	16,58	0	***	0,897	1,164	9045	1809	0,07
	Shared Profits	0,748	0,058	12,904	0	***	0,614	0,882	9045	1809	0,07
	No Profits	0,327	0,059	5,549	0	***	0,193	0,461	9045	1809	0,07
	Medium Removal	0,818	0,073	11,13	0	***	0,684	0,951	9045	1809	0,07
	High Removal	1,015	0,076	13,343	0	***	0,881	1,149	9045	1809	0,07
Malaysia	Constant	5,504	0,086	64,229	0	***	5,36	5,648	9030	1806	0,062
	Company	-0,09	0,039	-2,329	0,02	*	-0,192	0,011	9030	1806	0,062
	Experts Consulted	1,192	0,059	20,372	0	***	1,068	1,317	9030	1806	0,062
	Locals Consulted	1,195	0,059	20,399	0	***	1,07	1,319	9030	1806	0,062
	Shared Profits	0,641	0,053	12,155	0	***	0,516	0,765	9030	1806	0,062

No Profits	0,507	0,052	9,826	0	***	0,383	0,632	9030	1806	0,062
Medium Removal	0,18	0,065	2,782	0,005	**	0,056	0,305	9030	1806	0,062
High Removal	0,234	0,066	3,562	0	***	0,11	0,359	9030	1806	0,062

Note: Estimates from OLS regressions with clustered standard errors at the individual level. Reference levels: regional government, no consultation, private profits, small removal. Statistical inference is based on two-sided t-tests against a zero-null hypothesis. No adjustment for multiple comparisons was applied. Est. = estimates; s.e. = standard errors; t-stat. = t-statistics; p-val. = p-values; stars = "***" ~ p < 0.001, "**" ~ p < 0.01, "*" ~ p < 0.05; conf.low and conf.high delimit the confidence intervals; N of Vig. = number of vignettes (country-level); N of Resp. = number of respondents (country-level); r2 = r squared.

V. CDR in Backyard

Supplementary Note 3. Operationalization of CDR in Backyard.

To better understand respondents' propensity to support Carbon Dioxide Removal projects, we use an additional survey item measuring support for CDR facilities being implemented within a 10 km radius of respondents' homes (7 miles in the English-language version). This item was asked after the experimental section and included CDR applications as well as other energy technologies such as nuclear, wind, and solar (see Q9 in section 12 of this document). Respondents were only asked about the CDR technology they had read about for the experiment. For the purpose of this analysis, we only consider respondents' answers to the CDR items. For each CDR technology we assessed its two main components separately: main facility and storage for DACCS and BECCS, and rock crushing plant and rock spreading for ERW. For each component, respondents were asked to indicate their level of support on a 6-point scale: 1 = Strongly Oppose, 2 = Oppose, 3 = Slightly Oppose, 4 = Slightly Support, 5 = Support, 6 = Strongly Support. The full survey item is included in the last section of this document along with the rest of the questionnaire. In this analysis we first summed and averaged the responses to the two components of each technology, obtaining an index ranging from 1 to 6 in 0.5 increments. We then classified respondents into three groups based on this index: Opposers (1.0-2.5), Middle (3.0-4.5), Supporters (5.0-6.0). Below we include the percentages of respondents selecting each of the answer options for each item and for the recoded variable used in the analysis.

Supplementary Table 9. Distribution of responses across CDR items, in percentages.

Country	Item	Strongly oppose	Oppose	Slightly oppose	Slightly support	Support	Strongly support	Don't know	Not Assigned
United Kingdom	Underground storage site for CO_2	7.4	7.2	7.4	15.2	14	6.2	9.4	33.3
	Plant to directly capture $CO_2$from the air for DACCS	3.3	2.3	2.9	7.1	8.7	5.1	4.1	66.6
	Bioenergy plant with CO_2capture for BECCS	2.2	2.6	3.1	8.5	9.2	3.4	4.3	66.7
	Stone-crushing facility for enhanced rock weathering	1.9	2.9	3.9	7.8	8.7	2.9	5.2	66.7
	Spreading crushed rock on farmland for enhanced rock weathering	2	2	2.7	8.1	8.9	4.4	5.2	66.7
Italy	Underground storage site for CO_2	7.4	7.1	9.8	14.6	16.2	5.3	6.2	33.3

	Plant to directly capture CO_2 from the air for DACCS	1.9	2.2	3.1	7.9	11	4.3	2.9	66.7
	Bioenergy plant with CO_2 capture for BECCS	1.9	2.5	2.4	6.7	11.8	5.9	2.3	66.6
	Stone-crushing facility for enhanced rock weathering	2.5	3.9	4.9	8.4	7.9	1.6	4.1	66.7
	Spreading crushed rock on farmland for enhanced rock weathering	2.1	2.3	3.2	6.9	10.6	3.2	4.8	66.7
Norway	Underground storage site for CO_2	9.2	6.8	8.5	14.1	13.5	6	8.4	33.4
	Plant to directly capture CO_2 from the air for DACCS	4	3.3	3.8	8	7.2	2.5	4.4	66.7
	Bioenergy plant with CO_2 capture for BECCS	3.4	2.8	3.1	8.6	8.4	2.5	4.6	66.6
	Stone-crushing facility for enhanced rock weathering	4.3	3.7	6.1	6.9	6.3	1.5	4.4	66.6
	Spreading crushed rock on farmland for enhanced rock weathering	3	3.1	2.9	7.9	8.4	3.4	4.6	66.6
Brazil	Underground storage site for CO_2	5.2	5.7	9.3	14.9	16.5	12	3.1	33.4
	Plant to directly capture CO_2 from the air for DACCS	1.3	2.1	2.9	6.7	11	8.1	1.2	66.7
	Bioenergy plant with CO_2 capture for BECCS	1.3	1.5	3.3	7.3	10	9.1	0.8	66.7
	Stone-crushing facility for enhanced rock weathering	2	2.9	5.4	7.7	9.4	4.4	1.6	66.6
	Spreading crushed rock on farmland for enhanced rock weathering	1.4	2.4	3.8	8.8	9.2	5.7	2	66.6
Saudi Arabia	Underground storage site for CO_2	9.6	7.1	9.3	14.4	10.9	12	3.5	33.4
	Plant to directly capture CO_2 from the air for DACCS	2.7	2	3.5	9.1	6.8	8.2	1	66.7
	Bioenergy plant with CO_2 capture for BECCS	2.8	2.2	3.5	8	7.4	7.7	1.7	66.6
	Stone-crushing facility for enhanced rock weathering	2.7	2.7	4	8.7	6.5	6.8	1.9	66.6
	Spreading crushed rock on farmland for enhanced rock weathering	2.8	2.1	3.4	9.6	6.4	6.7	2.3	66.6
Malaysia	Underground storage site for CO_2	5.4	7	10	15.7	19	7.4	2.3	33.3

Plant to directly capture $CO_2$from the air for DACCS	1.9	2	2.9	7.9	11.7	5.6	1.3	66.6
Bioenergy plant with CO_2 capture for BECCS	2	2.3	3.7	8.7	11.6	4.1	0.9	66.7
Stone-crushing facility for enhanced rock weathering	2.4	2.8	4.8	8.8	9.3	3.7	1.4	66.7
Spreading crushed rock on farmland for enhanced rock weathering	2.7	3	5.9	8.6	8.5	3.4	1.3	66.7

Note: N=10,317 respondents, with a total of 51,585 observations. “Not Assigned” refers to the respondents who did not read about the corresponding CDR technology. “Underground storage for CO_2” was shown to respondents who read about DACCS and those who read about BECCS.

Supplementary Table 10. Distribution of the three groups as used in the analysis, in percentages.

	**DACCS**				**BECCS**				**ERW**			
**Country**	**Opp.**	**Mod.**	**Supp.**	**Tot**	**Opp.**	**Mod.**	**Supp.**	**Tot**	**Opp.**	**Mod.**	**Supp.**	**Tot**
United Kingdom	38.3	33.5	28.3	100	35.6	52.5	11.9	100	32.1	32.5	35.5	100
Italy	36.4	30.5	33.1	100	33.5	28.3	38.2	100	37.3	32.4	30.4	100
Norway	42.9	32.9	24.2	100	43.8	25.7	30.4	100	47.1	28.1	24.8	100
Brazil	29.7	28.9	41.4	100	26.8	28	45.2	100	32.3	31.9	35.8	100
Saudi Arabia	29.8	35.5	34.7	100	37.2	25.9	36.9	100	23.8	41.8	34.4	100
Malaysia	24	38.5	37.5	100	23.9	40.5	35.6	100	28.4	33	38.5	100

Note: N=10,313 respondents, with a total of 51,565 observations. Opp. = Opposers, Mod = Moderates, Supp. = Supporters, Tot = row sum. “Don’t know” answers have been excluded from the analysis. Values reflect percentages related to the technologies sub-samples in each country.

Supplementary Figure 2. Average marginal component effects of governance and technical features on public support for carbon removal projects (1-10 scale: 1= strongly oppose; 10=strongly support) across six countries and levels of CDR in Backyard.

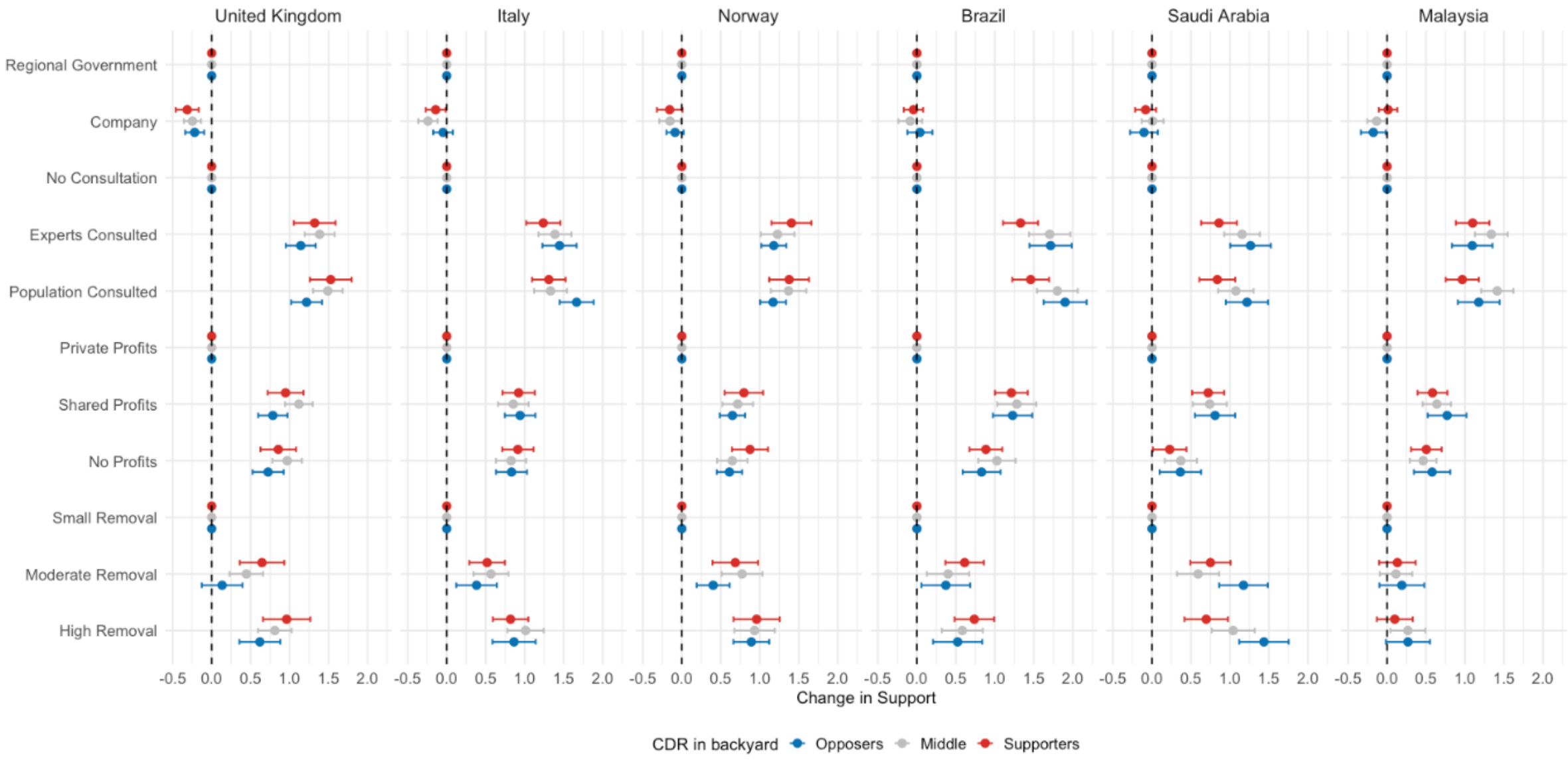

Note: N=10,313 respondents, with a total of 51,565 observations (five per participant). Average Marginal Component Effects (AMCE; unstandardized effect sizes) obtained interacting "CDR in backyard" with each of the socio-technical attributes of the vignette experiments. Calculated using ordinary least squares regressions (one per country) with individual clustered standard errors. Y-axis includes the features of the four socio-technical attributes. X-axis shows AMCEs on the support scale (1 = "strongly oppose", 10 = "strongly support"). The dashed vertical line highlights the base level 0.0 (i.e. for the base attributes: Government, No Consultation, Private Profits, Small Removal). The mean value of the AMCE is represented by the dots and lines represent the 95% confidence intervals for two-sided t-tests against a zero-null hypothesis. No adjustment for multiple comparisons was applied.

Supplementary Table 11. Estimated average marginal component effects of governance and technical features on public support for carbon removal projects (1-10 scale: 1= strongly oppose; 10=strongly support) across six countries and levels of CDR in Backyard.

Country	Attributes	Est.	s.e.	t-stat.	p-val.	stars	conf. low	conf. high	N of Vig.	N of Resp.	r2
United Kingdom	Constant	3,299	0,138	23,89	0	***	3,061	3,536	8180	1636	0,24
	Company	-0,218	0,062	-3,52	0	***	-0,38	-0,05	8180	1636	0,24
	Experts Consulted	1,144	0,086	13,23	0	***	0,943	1,345	8180	1636	0,24
	Locals Consulted	1,218	0,089	13,66	0	***	1,016	1,42	8180	1636	0,24
	Shared Profits	0,785	0,085	9,291	0	***	0,584	0,987	8180	1636	0,24
	No Profits	0,724	0,09	8,054	0	***	0,523	0,925	8180	1636	0,24
	Medium Removal	0,134	0,117	1,144	0,253		-0,07	0,337	8180	1636	0,24
	High Removal	0,618	0,118	5,236	0	***	0,416	0,82	8180	1636	0,24
	Experts Consulted##Moderate	0,242	0,122	1,983	0,047	*	-0,04	0,519	8180	1636	0,24
	Experts Consulted##Supporters	0,178	0,148	1,202	0,229		-0,13	0,491	8180	1636	0,24
	Locals Consulted##Moderate	0,272	0,123	2,208	0,027	*	-0,01	0,549	8180	1636	0,24
	Locals Consulted##Supporters	0,31	0,15	2,069	0,039	*	-0	0,623	8180	1636	0,24
	No Profits##Moderate	0,245	0,124	1,977	0,048	*	-0,03	0,522	8180	1636	0,24
	No Profits##Supporters	0,131	0,137	0,959	0,338		-0,18	0,443	8180	1636	0,24

	Shared Profits##Moderate	0,334	0,116	2,869	0,004	**	0,056	0,611	8180	1636	0,24
	Shared Profits##Supporters	0,163	0,133	1,219	0,223		-0,15	0,474	8180	1636	0,24
	High Removal##Moderate	0,193	0,153	1,263	0,207		-0,08	0,47	8180	1636	0,24
	High Removal##Supporters	0,344	0,181	1,907	0,057		0,031	0,658	8180	1636	0,24
	Medium Removal##Moderate	0,311	0,152	2,041	0,041	*	0,033	0,588	8180	1636	0,24
	Medium Removal##Supporters	0,512	0,174	2,939	0,003	**	0,199	0,824	8180	1636	0,24
	Company##Moderate	-0,028	0,084	-0,34	0,734		-0,26	0,198	8180	1636	0,24
	Company##Supporters	-0,096	0,098	-0,98	0,326		-0,35	0,159	8180	1636	0,24
	Moderate	1,043	0,189	5,511	0	***	0,722	1,365	8180	1636	0,24
	Supporters	1,821	0,23	7,903	0	***	1,459	2,183	8180	1636	0,24
Italy	Constant	3,447	0,133	25,88	0	***	3,222	3,673	8500	1700	0,22
	Company	-0,048	0,064	-0,75	0,456		-0,21	0,112	8500	1700	0,22
	Experts Consulted	1,447	0,099	14,66	0	***	1,251	1,643	8500	1700	0,22
	Locals Consulted	1,667	0,098	16,93	0	***	1,471	1,863	8500	1700	0,22
	Shared Profits	0,941	0,088	10,67	0	***	0,744	1,138	8500	1700	0,22
	No Profits	0,831	0,09	9,281	0	***	0,636	1,027	8500	1700	0,22
	Medium Removal	0,382	0,117	3,263	0,001	**	0,186	0,578	8500	1700	0,22
	High Removal	0,863	0,125	6,918	0	***	0,668	1,058	8500	1700	0,22
	Experts Consulted##Moderate	-0,058	0,137	-0,42	0,672		-0,35	0,231	8500	1700	0,22
	Experts Consulted##Supporters	-0,208	0,14	-1,49	0,135		-0,49	0,073	8500	1700	0,22
	Locals Consulted##Moderate	-0,335	0,137	-2,45	0,014	*	-0,63	-0,05	8500	1700	0,22
	Locals Consulted##Supporters	-0,356	0,138	-2,58	0,01	**	-0,64	-0,08	8500	1700	0,22
	No Profits##Moderate	-0,006	0,125	-0,05	0,962		-0,3	0,283	8500	1700	0,22
	No Profits##Supporters	0,081	0,127	0,638	0,523		-0,2	0,362	8500	1700	0,22
	Shared Profits##Moderate	-0,087	0,125	-0,7	0,484		-0,38	0,202	8500	1700	0,22
	Shared Profits##Supporters	-0,019	0,128	-0,15	0,884		-0,3	0,262	8500	1700	0,22
	High Removal##Moderate	0,149	0,163	0,911	0,362		-0,14	0,437	8500	1700	0,22
	High Removal##Supporters	-0,045	0,161	-0,28	0,782		-0,33	0,236	8500	1700	0,22
	Medium Removal##Moderate	0,185	0,155	1,197	0,232		-0,1	0,474	8500	1700	0,22
	Medium Removal##Supporters	0,136	0,156	0,869	0,385		-0,15	0,416	8500	1700	0,22
	Company##Moderate	-0,193	0,09	-2,15	0,032	*	-0,43	0,043	8500	1700	0,22
	Company##Supporters	-0,094	0,092	-1,03	0,304		-0,32	0,135	8500	1700	0,22
	Moderate	1,314	0,191	6,896	0	***	0,979	1,65	8500	1700	0,22
	Supporters	2,136	0,193	11,08	0	***	1,814	2,458	8500	1700	0,22
Norway	Constant	2,602	0,108	24,14	0	***	2,391	2,814	8330	1666	0,25
	Company	-0,087	0,056	-1,55	0,121		-0,24	0,062	8330	1666	0,25
	Experts Consulted	1,18	0,073	16,27	0	***	0,997	1,362	8330	1666	0,25
	Locals Consulted	1,171	0,075	15,65	0	***	0,989	1,353	8330	1666	0,25
	Shared Profits	0,649	0,073	8,896	0	***	0,467	0,832	8330	1666	0,25
	No Profits	0,611	0,073	8,372	0	***	0,429	0,793	8330	1666	0,25
	Medium Removal	0,402	0,095	4,228	0	***	0,222	0,582	8330	1666	0,25

	High Removal	0,892	0,103	8,64	0	***	0,708	1,076	8330	1666	0,25
	Experts Consulted##Moderate	0,048	0,121	0,399	0,69		-0,24	0,34	8330	1666	0,25
	Experts Consulted##Supporters	0,227	0,136	1,666	0,096		-0,07	0,525	8330	1666	0,25
	Locals Consulted##Moderate	0,198	0,127	1,559	0,119		-0,09	0,489	8330	1666	0,25
	Locals Consulted##Supporters	0,206	0,137	1,499	0,134		-0,09	0,504	8330	1666	0,25
	No Profits##Moderate	0,037	0,114	0,32	0,749		-0,25	0,327	8330	1666	0,25
	No Profits##Supporters	0,264	0,127	2,072	0,038	*	-0,03	0,562	8330	1666	0,25
	Shared Profits##Moderate	0,067	0,115	0,585	0,559		-0,22	0,358	8330	1666	0,25
	Shared Profits##Supporters	0,148	0,133	1,114	0,265		-0,15	0,447	8330	1666	0,25
	High Removal##Moderate	0,042	0,155	0,267	0,789		-0,25	0,332	8330	1666	0,25
	High Removal##Supporters	0,068	0,168	0,402	0,688		-0,23	0,368	8330	1666	0,25
	Medium Removal##Moderate	0,371	0,152	2,445	0,015	*	0,082	0,661	8330	1666	0,25
	Medium Removal##Supporters	0,284	0,163	1,746	0,081		-0,02	0,583	8330	1666	0,25
	Company##Moderate	-0,066	0,09	-0,74	0,462		-0,3	0,172	8330	1666	0,25
	Company##Supporters	-0,07	0,1	-0,7	0,487		-0,31	0,174	8330	1666	0,25
	Moderate	1,386	0,175	7,913	0	***	1,052	1,721	8330	1666	0,25
	Supporters	2,085	0,204	10,24	0	***	1,738	2,431	8330	1666	0,25
Brazil	Constant	4,282	0,179	23,97	0	***	4,007	4,556	8855	1771	0,17
	Company	0,038	0,082	0,468	0,64		-0,16	0,234	8855	1771	0,17
	Experts Consulted	1,716	0,122	14,02	0	***	1,476	1,955	8855	1771	0,17
	Locals Consulted	1,902	0,124	15,31	0	***	1,662	2,142	8855	1771	0,17
	Shared Profits	1,228	0,114	10,82	0	***	0,987	1,469	8855	1771	0,17
	No Profits	0,83	0,109	7,579	0	***	0,59	1,069	8855	1771	0,17
	Medium Removal	0,371	0,141	2,638	0,008	**	0,13	0,612	8855	1771	0,17
	High Removal	0,522	0,142	3,68	0	***	0,283	0,761	8855	1771	0,17
	Experts Consulted##Moderate	-0,011	0,171	-0,07	0,947		-0,35	0,328	8855	1771	0,17
	Experts Consulted##Supporters	-0,386	0,159	-2,43	0,015	*	-0,7	-0,07	8855	1771	0,17
	Locals Consulted##Moderate	-0,099	0,171	-0,58	0,561		-0,44	0,24	8855	1771	0,17
	Locals Consulted##Supporters	-0,442	0,163	-2,7	0,007	**	-0,76	-0,13	8855	1771	0,17
	No Profits##Moderate	0,197	0,154	1,282	0,2		-0,14	0,536	8855	1771	0,17
	No Profits##Supporters	0,056	0,145	0,384	0,701		-0,26	0,371	8855	1771	0,17
	Shared Profits##Moderate	0,054	0,16	0,338	0,736		-0,29	0,394	8855	1771	0,17
	Shared Profits##Supporters	-0,016	0,148	-0,11	0,912		-0,33	0,299	8855	1771	0,17
	High Removal##Moderate	0,06	0,185	0,324	0,746		-0,28	0,4	8855	1771	0,17
	High Removal##Supporters	0,215	0,182	1,178	0,239		-0,1	0,529	8855	1771	0,17
	Medium Removal##Moderate	0,028	0,187	0,152	0,88		-0,31	0,367	8855	1771	0,17
	Medium Removal##Supporters	0,241	0,179	1,346	0,178		-0,08	0,557	8855	1771	0,17
	Company##Moderate	-0,124	0,113	-1,1	0,273		-0,4	0,154	8855	1771	0,17
	Company##Supporters	-0,083	0,103	-0,8	0,423		-0,34	0,175	8855	1771	0,17

	Moderate	0,506	0,246	2,059	0,04	*	0,115	0,898	8855	1771	0,17
	Supporters	1,588	0,239	6,646	0	***	1,226	1,951	8855	1771	0,17
Saudi Arabia	Constant	4,546	0,173	26,33	0	***	4,268	4,824	8830	1766	0,12
	Company	-0,104	0,091	-1,14	0,254		-0,3	0,093	8830	1766	0,12
	Experts Consulted	1,265	0,118	10,76	0	***	1,025	1,505	8830	1766	0,12
	Locals Consulted	1,219	0,122	9,977	0	***	0,978	1,459	8830	1766	0,12
	Shared Profits	0,809	0,116	6,975	0	***	0,57	1,049	8830	1766	0,12
	No Profits	0,363	0,12	3,033	0,002	**	0,123	0,604	8830	1766	0,12
	Medium Removal	1,174	0,141	8,353	0	***	0,934	1,415	8830	1766	0,12
	High Removal	1,436	0,143	10,06	0	***	1,195	1,678	8830	1766	0,12
	Experts Consulted##Moderate	-0,109	0,156	-0,7	0,486		-0,44	0,221	8830	1766	0,12
	Experts Consulted##Supporters	-0,405	0,156	-2,59	0,01	**	-0,73	-0,08	8830	1766	0,12
	Locals Consulted##Moderate	-0,143	0,159	-0,9	0,368		-0,47	0,187	8830	1766	0,12
	Locals Consulted##Supporters	-0,381	0,16	-2,38	0,017	*	-0,71	-0,05	8830	1766	0,12
	No Profits##Moderate	0,006	0,152	0,041	0,967		-0,32	0,336	8830	1766	0,12
	No Profits##Supporters	-0,137	0,154	-0,89	0,374		-0,47	0,191	8830	1766	0,12
	Shared Profits##Moderate	-0,069	0,152	-0,45	0,651		-0,4	0,261	8830	1766	0,12
	Shared Profits##Supporters	-0,089	0,149	-0,6	0,55		-0,42	0,238	8830	1766	0,12
	High Removal##Moderate	-0,395	0,189	-2,08	0,037	*	-0,73	-0,07	8830	1766	0,12
	High Removal##Supporters	-0,741	0,19	-3,89	0	***	-1,07	-0,41	8830	1766	0,12
	Medium Removal##Moderate	-0,584	0,186	-3,14	0,002	**	-0,92	-0,25	8830	1766	0,12
	Medium Removal##Supporters	-0,424	0,182	-2,33	0,02	*	-0,75	-0,1	8830	1766	0,12
	Company##Moderate	0,112	0,116	0,966	0,334		-0,16	0,381	8830	1766	0,12
	Company##Supporters	0,022	0,114	0,19	0,85		-0,25	0,289	8830	1766	0,12
	Moderate	0,816	0,233	3,498	0	***	0,436	1,196	8830	1766	0,12
	Supporters	2,056	0,234	8,771	0	***	1,677	2,436	8830	1766	0,12
Malaysia	Constant	4,434	0,166	26,65	0	***	4,161	4,706	8870	1774	0,17
	Company	-0,177	0,082	-2,16	0,031	*	-0,37	0,016	8870	1774	0,17
	Experts Consulted	1,093	0,117	9,33	0	***	0,857	1,329	8870	1774	0,17
	Locals Consulted	1,176	0,121	9,729	0	***	0,94	1,412	8870	1774	0,17
	Shared Profits	0,77	0,112	6,879	0	***	0,535	1,005	8870	1774	0,17
	No Profits	0,577	0,105	5,493	0	***	0,341	0,813	8870	1774	0,17
	Medium Removal	0,189	0,13	1,458	0,145		-0,05	0,424	8870	1774	0,17
	High Removal	0,267	0,127	2,097	0,036	*	0,032	0,502	8870	1774	0,17
	Experts Consulted##Moderate	0,245	0,151	1,625	0,104		-0,06	0,55	8870	1774	0,17
	Experts Consulted##Supporters	0,005	0,152	0,032	0,974		-0,3	0,311	8870	1774	0,17
	Locals Consulted##Moderate	0,238	0,152	1,559	0,119		-0,07	0,543	8870	1774	0,17
	Locals Consulted##Supporters	-0,211	0,154	-1,37	0,171		-0,52	0,096	8870	1774	0,17
	No Profits##Moderate	-0,115	0,13	-0,88	0,378		-0,42	0,191	8870	1774	0,17
	No Profits##Supporters	-0,074	0,137	-0,54	0,591		-0,38	0,232	8870	1774	0,17

Shared Profits##Moderate	-0,132	0,139	-0,95	0,341		-0,44	0,173	8870	1774	0,17
Shared Profits##Supporters	-0,188	0,141	-1,33	0,184		-0,49	0,118	8870	1774	0,17
High Removal##Moderate	-0,001	0,163	-0	0,997		-0,31	0,304	8870	1774	0,17
High Removal##Supporters	-0,169	0,164	-1,03	0,304		-0,48	0,138	8870	1774	0,17
Medium Removal##Moderate	-0,075	0,16	-0,47	0,638		-0,38	0,23	8870	1774	0,17
Medium Removal##Supporters	-0,057	0,167	-0,34	0,734		-0,36	0,249	8870	1774	0,17
Company##Moderate	0,042	0,102	0,415	0,678		-0,21	0,292	8870	1774	0,17
Company##Supporters	0,189	0,102	1,862	0,063		-0,06	0,439	8870	1774	0,17
Moderate	0,815	0,21	3,88	0	***	0,463	1,167	8870	1774	0,17
Supporters	2,179	0,221	9,871	0	***	1,824	2,533	8870	1774	0,17

Note: N=10,313 respondents, with a total of 51,565 observations (five per participant). Estimates from OLS regressions with clustered standard errors at the individual level (in parentheses). Reference levels: regional government, no consultation, private profits, small removal. Statistical inference is based on two-sided t-tests against a zero-null hypothesis. No adjustment for multiple comparisons was applied. Est. = estimates; s.e. = standard errors; t-stat. = t-statistics; p-val. = p-values; stars = "***" ~ p < 0.001, "**" ~ p < 0.01, "*" ~ p < 0.05; conf.low and conf.high delimit the confidence intervals; N of Vig. = number of vignettes (country-level); N of Resp. = number of respondents (country-level); r2 = r squared.

VI. Figure 3 Corresponding Table

Supplementary Table 12. Estimated average marginal component effects with interaction effects between technical attributes of carbon removal projects (1-10 scale: 1= strongly oppose; 10=strongly support) and procedural fairness or distributive fairness across six countries.

Country	Attributes	Est.	s.e.	t-stat.	p-val.	stars	conf.low	conf.high	N of Vig.	N of Resp.	r2
United Kingdom	Constant	4,291	0,12	35,773	0	***	4,078	4,504	8995	1799	0,103
	Company	-0,248	0,036	-6,934	0	***	-0,35	-0,15	8995	1799	0,103
	Experts Consulted	1,152	0,108	10,626	0	***	0,934	1,37	8995	1799	0,103
	Locals Consulted	1,355	0,109	12,443	0	***	1,137	1,573	8995	1799	0,103
	Shared Profits	0,872	0,102	8,581	0	***	0,653	1,09	8995	1799	0,103
	No Profits	0,711	0,107	6,66	0	***	0,492	0,929	8995	1799	0,103
	Medium Removal	0,246	0,157	1,569	0,117		-0,05	0,538	8995	1799	0,103
	High Removal	0,588	0,147	4,003	0	***	0,315	0,86	8995	1799	0,103
	Medium Removal ## Locals Consulted	-0,047	0,159	-0,293	0,769		-0,36	0,262	8995	1799	0,103
	High Removal ## Locals Consulted	0,076	0,161	0,475	0,635		-0,23	0,384	8995	1799	0,103
	Medium Removal ## Experts Consulted	0,082	0,16	0,515	0,607		-0,23	0,391	8995	1799	0,103
	High Removal ## Experts Consulted	0,229	0,161	1,421	0,155		-0,08	0,537	8995	1799	0,103
	Medium Removal ## No Profits	0,167	0,153	1,091	0,275		-0,14	0,476	8995	1799	0,103
	High Removal ## No Profits	0,093	0,152	0,608	0,543		-0,22	0,401	8995	1799	0,103
	Medium Removal ## Shared Profits	0,141	0,151	0,935	0,35		-0,17	0,449	8995	1799	0,103
	High Removal ## Shared Profits	-0,011	0,15	-0,073	0,942		-0,32	0,298	8995	1799	0,103
Italy	Constant	4,331	0,12	35,993	0	***	4,122	4,54	9085	1817	0,121
	Company	-0,101	0,037	-2,748	0,006	**	-0,2	-0	9085	1817	0,121
	Experts Consulted	1,499	0,111	13,482	0	***	1,286	1,712	9085	1817	0,121
	Locals Consulted	1,697	0,112	15,172	0	***	1,484	1,911	9085	1817	0,121

	Shared Profits	0,918	0,105	8,759	0	***	0,704	1,131	9085	1817	0,121
	No Profits	0,915	0,111	8,261	0	***	0,702	1,128	9085	1817	0,121
	Medium Removal	0,838	0,158	5,3	0	***	0,552	1,124	9085	1817	0,121
	High Removal	1,093	0,15	7,269	0	***	0,826	1,36	9085	1817	0,121
	Medium Removal ## Locals Consulted	-0,291	0,163	-1,783	0,075		-0,59	0,011	9085	1817	0,121
	High Removal ## Locals Consulted	-0,472	0,158	-2,989	0,003	**	-0,78	-0,17	9085	1817	0,121
	Medium Removal ## Experts Consulted	-0,303	0,164	-1,85	0,064		-0,6	-0	9085	1817	0,121
	High Removal ## Experts Consulted	-0,084	0,159	-0,53	0,596		-0,39	0,217	9085	1817	0,121
	Medium Removal ## No Profits	-0,135	0,154	-0,876	0,381		-0,44	0,167	9085	1817	0,121
	High Removal ## No Profits	0,021	0,155	0,134	0,894		-0,28	0,323	9085	1817	0,121
	Medium Removal ## Shared Profits	-0,164	0,153	-1,07	0,285		-0,47	0,138	9085	1817	0,121
	High Removal ## Shared Profits	0,035	0,148	0,239	0,811		-0,27	0,338	9085	1817	0,121
Norway	Constant	3,61	0,112	32,228	0	***	3,389	3,832	9085	1817	0,091
	Company	-0,107	0,039	-2,78	0,005	**	-0,21	-0	9085	1817	0,091
	Experts Consulted	1,202	0,105	11,409	0	***	0,976	1,427	9085	1817	0,091
	Locals Consulted	1,191	0,107	11,104	0	***	0,965	1,417	9085	1817	0,091
	Shared Profits	0,685	0,103	6,644	0	***	0,461	0,91	9085	1817	0,091
	No Profits	0,622	0,102	6,108	0	***	0,395	0,849	9085	1817	0,091
	Medium Removal	0,573	0,154	3,721	0	***	0,269	0,877	9085	1817	0,091
	High Removal	0,728	0,141	5,174	0	***	0,446	1,01	9085	1817	0,091
	Medium Removal ## Locals Consulted	0,102	0,167	0,609	0,543		-0,22	0,421	9085	1817	0,091
	High Removal ## Locals Consulted	0,16	0,165	0,967	0,333		-0,16	0,48	9085	1817	0,091
	Medium Removal ## Experts Consulted	-0,135	0,167	-0,809	0,418		-0,45	0,183	9085	1817	0,091
	High Removal ## Experts Consulted	0,332	0,159	2,085	0,037	*	0,013	0,651	9085	1817	0,091
	Medium Removal ## No Profits	0,015	0,156	0,096	0,924		-0,3	0,334	9085	1817	0,091
	High Removal ## No Profits	0,165	0,155	1,062	0,288		-0,16	0,485	9085	1817	0,091
	Medium Removal ## Shared Profits	-0,033	0,16	-0,208	0,836		-0,35	0,285	9085	1817	0,091
	High Removal ## Shared Profits	-0,041	0,156	-0,266	0,79		-0,36	0,278	9085	1817	0,091
Brazil	Constant	5,145	0,141	36,563	0	***	4,912	5,378	9020	1804	0,118
	Company	-0,035	0,042	-0,828	0,408		-0,14	0,074	9020	1804	0,118
	Experts Consulted	1,442	0,124	11,594	0	***	1,204	1,68	9020	1804	0,118
	Locals Consulted	1,786	0,126	14,182	0	***	1,547	2,025	9020	1804	0,118
	Shared Profits	1,097	0,122	8,972	0	***	0,859	1,336	9020	1804	0,118
	No Profits	0,809	0,123	6,597	0	***	0,572	1,045	9020	1804	0,118
	Medium Removal	0,353	0,187	1,885	0,059		0,031	0,675	9020	1804	0,118
	High Removal	0,546	0,174	3,139	0,002	**	0,25	0,843	9020	1804	0,118
	Medium Removal ## Locals Consulted	-0,158	0,179	-0,881	0,378		-0,5	0,18	9020	1804	0,118
	High Removal ## Locals Consulted	-0,082	0,178	-0,459	0,646		-0,42	0,256	9020	1804	0,118
	Medium Removal ## Experts Consulted	0,138	0,181	0,766	0,444		-0,2	0,475	9020	1804	0,118
	High Removal ## Experts Consulted	0,17	0,176	0,965	0,334		-0,17	0,506	9020	1804	0,118
	Medium Removal ## No Profits	0,153	0,181	0,845	0,398		-0,18	0,489	9020	1804	0,118

	High Removal ## No Profits	0,028	0,173	0,161	0,872		-0,31	0,364	9020	1804	0,118
	Medium Removal ## Shared Profits	0,233	0,173	1,351	0,177		-0,11	0,571	9020	1804	0,118
	High Removal ## Shared Profits	0,099	0,171	0,583	0,56		-0,24	0,435	9020	1804	0,118
Saudi Arabia	Constant	5,488	0,142	38,664	0	***	5,255	5,72	9045	1809	0,071
	Company	-0,058	0,044	-1,313	0,189		-0,17	0,051	9045	1809	0,071
	Experts Consulted	1,053	0,128	8,244	0	***	0,815	1,291	9045	1809	0,071
	Locals Consulted	1,016	0,13	7,814	0	***	0,776	1,255	9045	1809	0,071
	Shared Profits	0,972	0,122	7,995	0	***	0,734	1,21	9045	1809	0,071
	No Profits	0,356	0,125	2,843	0,004	**	0,118	0,594	9045	1809	0,071
	Medium Removal	0,842	0,182	4,619	0	***	0,521	1,162	9045	1809	0,071
	High Removal	1,216	0,171	7,111	0	***	0,918	1,514	9045	1809	0,071
	Medium Removal ## Locals Consulted	0,035	0,183	0,191	0,849		-0,3	0,373	9045	1809	0,071
	High Removal ## Locals Consulted	-0,001	0,183	-0,003	0,998		-0,34	0,338	9045	1809	0,071
	Medium Removal ## Experts Consulted	0,029	0,178	0,161	0,872		-0,31	0,365	9045	1809	0,071
	High Removal ## Experts Consulted	0,016	0,176	0,089	0,929		-0,32	0,352	9045	1809	0,071
	Medium Removal ## No Profits	0,132	0,175	0,755	0,45		-0,21	0,47	9045	1809	0,071
	High Removal ## No Profits	-0,215	0,18	-1,195	0,232		-0,55	0,122	9045	1809	0,071
	Medium Removal ## Shared Profits	-0,264	0,169	-1,556	0,12		-0,6	0,073	9045	1809	0,071
	High Removal ## Shared Profits	-0,403	0,169	-2,38	0,017	*	-0,74	-0,07	9045	1809	0,071
Malaysia	Constant	5,505	0,128	42,871	0	***	5,286	5,725	9030	1806	0,064
	Company	-0,09	0,039	-2,314	0,021	*	-0,19	0,012	9030	1806	0,064
	Experts Consulted	1,28	0,118	10,857	0	***	1,057	1,503	9030	1806	0,064
	Locals Consulted	1,332	0,114	11,708	0	***	1,108	1,556	9030	1806	0,064
	Shared Profits	0,569	0,108	5,285	0	***	0,346	0,792	9030	1806	0,064
	No Profits	0,35	0,111	3,157	0,002	**	0,126	0,573	9030	1806	0,064
	Medium Removal	0,294	0,173	1,701	0,089		-0,01	0,596	9030	1806	0,064
	High Removal	0,187	0,158	1,182	0,237		-0,09	0,466	9030	1806	0,064
	Medium Removal ## Locals Consulted	-0,317	0,165	-1,92	0,055		-0,63	-0	9030	1806	0,064
	High Removal ## Locals Consulted	-0,149	0,167	-0,889	0,374		-0,47	0,167	9030	1806	0,064
	Medium Removal ## Experts Consulted	-0,338	0,168	-2,01	0,044	*	-0,65	-0,02	9030	1806	0,064
	High Removal ## Experts Consulted	-0,05	0,169	-0,299	0,765		-0,37	0,265	9030	1806	0,064
	Medium Removal ## No Profits	0,323	0,161	2,01	0,044	*	0,007	0,639	9030	1806	0,064
	High Removal ## No Profits	0,19	0,162	1,172	0,241		-0,13	0,506	9030	1806	0,064
	Medium Removal ## Shared Profits	-0,008	0,16	-0,051	0,96		-0,32	0,308	9030	1806	0,064
	High Removal ## Shared Profits	0,149	0,158	0,945	0,345		-0,17	0,465	9030	1806	0,064

Note: Estimates from OLS regressions with clustered standard errors at the individual level (in parentheses). Reference levels: regional government, no consultation, private profits, small removal. Statistical inference is based on two-sided t-tests against a zero-null hypothesis. No adjustment for multiple comparisons was applied. Est. = estimates; s.e. = standard errors; t-stat. = t-statistics; p-val. = p-values; stars = "***" ~ p < 0.001, "**" ~ p < 0.01, "*" ~ p < 0.05; conf.low and conf.high delimit the confidence intervals; N of Vig. = number of vignettes (country-level); N of Resp. = number of respondents (country-level); r2 = r squared.

VII. Figure 4 Additional Items

The degrees of engagement included in this item were inspired by Arnstein's (1969) ladder of participation in community planning. Differentiating participation or engagement according to different degrees or intensities of involvement has become a widely adopted typology in participation scholarship and practice (see e.g., Fritz et al. 2024; Smith et al. 2023). In addition to different degrees of involvement and transfer of decision power, the item also differentiated different decision-steps and timings of involvement.

Supplementary Figure 3. Public preferences for fairly engaging local communities in governance of carbon removal projects, across countries (percentages, countries ordered from highest to lowest on "Local population votes").

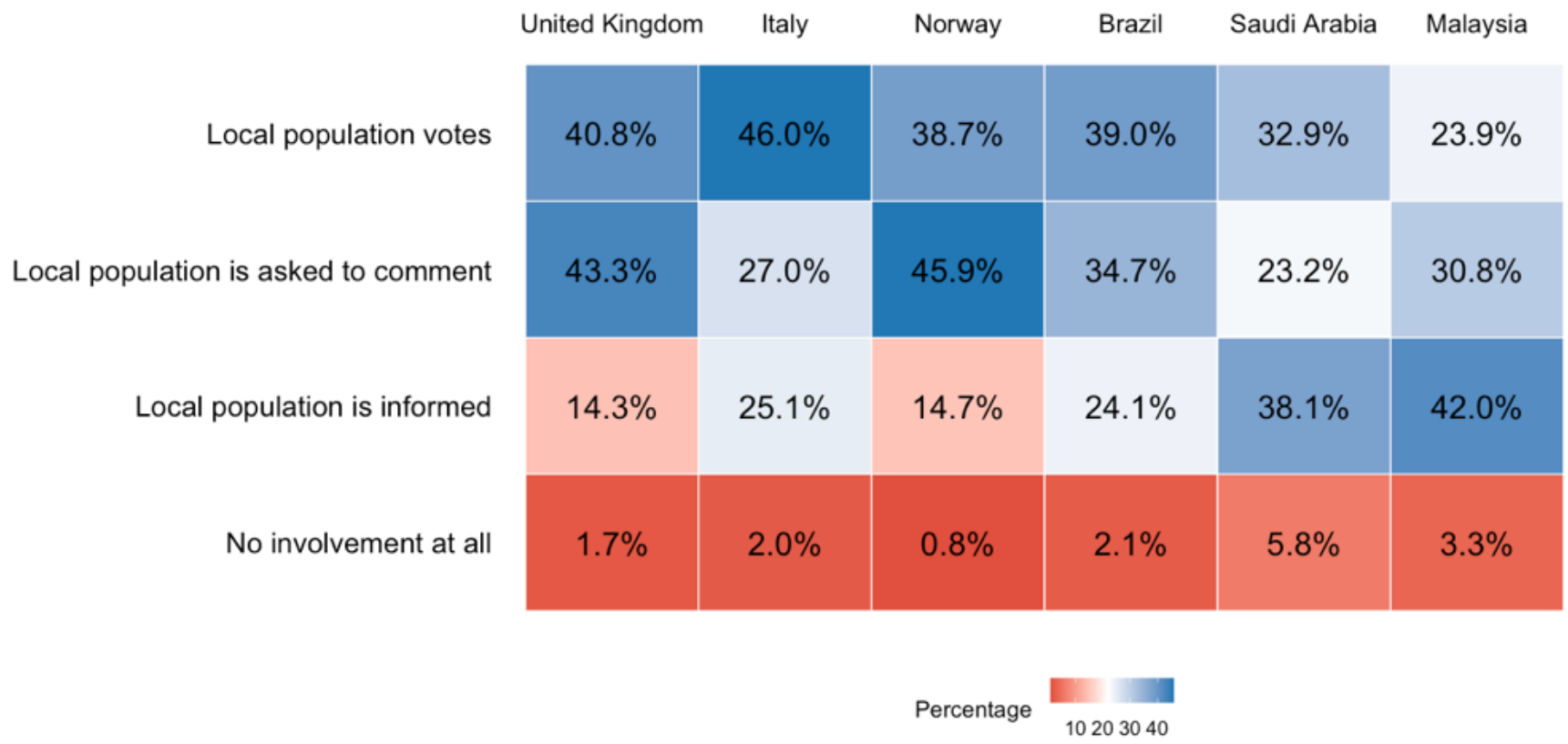

Note: N=10,592 respondents. After completing all the vignette evaluations, respondents were asked "When starting a new [DACCS/BECCS/ERW] project, there are various decisions to take and different ways to involve the local population. When it comes to the planning of such a project, what level of involvement of the local population do you think is fair for each of the following decisions?" This question was asked for three decisions: (i) whether the project is permitted, (ii) the location where to implement the project, (iii) how to distribute profits. Only decision (ii) shown in the figure; the other two are included in main text Fig. 4, Supplementary Figure 3. "Don't know" responses (n=260 respondents) have not been included in the analysis. Each cell shows the percentage of respondents, in each country, that selected this type of involvement as most fair.

Supplementary Figure 4. Public preferences for fairly engaging local communities in governance of carbon removal projects, across countries (percentages, countries ordered from highest to lowest on "Local population votes").

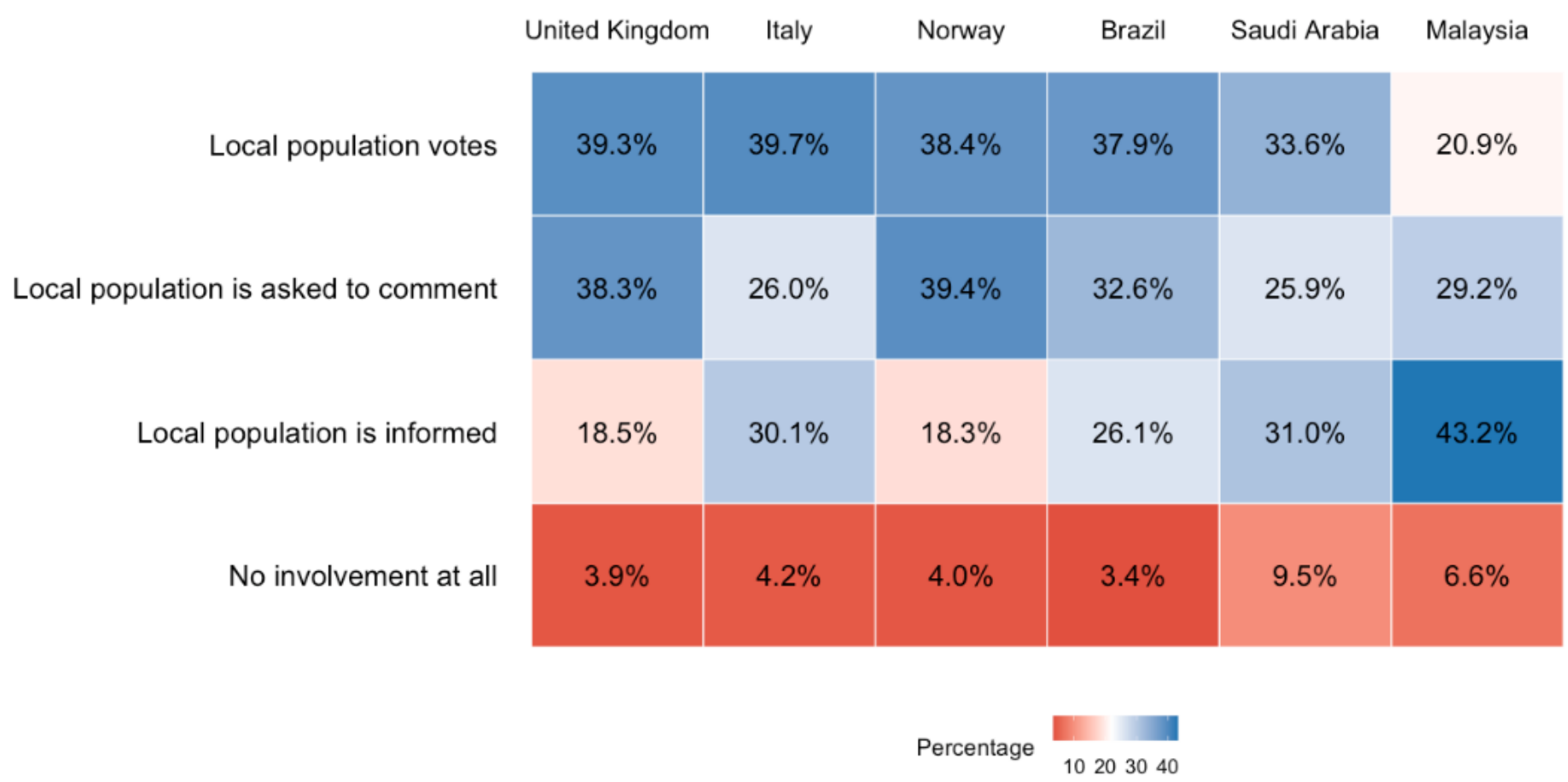

Note: N=10,407 respondents. After completing all the vignette evaluations, respondents were asked "When starting a new [DACCS/BECCS/ERW] project, there are various decisions to take and different ways to involve the local population. When it comes to the planning of such a project, what level of involvement of the local

population do you think is fair for each of the following decisions?” This question was asked for three decisions: (i) whether the project is permitted, (ii) the location where to implement the project, (iii) how to distribute profits. Only decision (ii) shown in the figure; the other two are included in main text Fig. 4, Supplementary Figure 4. “Don’t know” responses (n=445 respondents) have not been included in the analysis. Each cell shows the percentage of respondents, in each country, that selected this type of involvement as most fair.

VIII. Individual Characteristics

Supplementary Table 13. Intra Class Correlation.

	ICC
**United Kingdom**	0,478
**Italy**	0,455
**Norway**	0,523
**Brazil**	0,369
**Saudi Arabia**	0,384
**Malaysia**	0,441

Supplementary Note 4. Operationalization.

Here we describe the operationalization of the covariates employed in the analysis. The full wording of each item is available in section 12 of this document.

Age. Q1. Employed as continuous in its recoded version provided by the survey provider including 6 intervals: 1=18-24 (reference), 2=25-34, 3=35-44, 4=45-54, 5=55-64, 6=65-75.

Gender. Q2. Employed as dummy where 1=Female (reference) and 2=Male. 3. Non-binary, other and refer not to say have been excluded from the analysis for they include too few cases.

Education. Q3. Employed as dummy in its recoded version provided by the survey provider where 1=Low (reference) and 2=High. Originally measured with country specific categories, visible in the last section of this document.

Income. Q20. Employed as dummy in its recoded version provided by the survey provider where 1=Low (reference) and 2=High. Originally measured with country specific categories, visible in the last section of this document.

Ideology. Q17. Employed as continuous where 0=Furthest to the left (reference) and 10=Furthest to the right. Since this measure of political ideology is constructed around western political systems and might not translate as well in the non-western countries included in our analysis, we complement it with a measure of economic liberalism.

Economic Liberalism. Q18a and Q18b. Measured though respondents’ agreement with six statements about the role that the “government” should have in the economic management of a country (e.g. “Businesses and farms should operate without governmental subsidies”) and on the life of individuals. For each statement respondents were asked to indicate their level of agreement on a 4 point scale where 1=strongly disagree and 4=strongly agree. The items have been summed and averaged to construct a continuous index where low scores signify less agreement with economic liberalist ideas (reference) and high scores signify more agreement. Following a chronbach’s alpha analysis with *psych::alpha()*, statement 2 in Q18b, the only item reverse coded, has been excluded (making the raw alpha increase from .65 to .72).

CDR Familiarity. Q9. Respondents were asked to indicate their level of familiarity with the CDR technology they were assigned to for the experiment on a 4 point scale where 1= yes, I

had heard a lot about this, 2=yes, I had heard a little about this, 3= no, I had not heard about this, 4=no, I had not heard about this, but I have heard of other carbon removal methods before. For this analysis we recoded this variable so that 0=familiarity(1+2), 1=familiarity with other CDR (4), 2=complete lack of familiarity with CDR (3). Here employed as continuous.

CDR Risk perception. Q10. Employed as dummy where 0=benefits (slightly+strongly) overweigh risks (reference) and 1=risks (slightly+strongly) overweigh benefits. This was obtaining by recoding the original 4 point variable.

Climate Change Seriousness. Q12. Employed as continuous where 0=not at all serious (reference) and 10=very serious.

Climate Change Apathy. Q13. Measured through respondents' agreement with four statements about the possibility or ability of humanity, individuals or institutions to take action against climate change (e.g. "It is not in humans' power to do anything about climate change"). For each statement respondents were asked to indicate their level of agreement on a 4 point scale where 1=strongly disagree and 4=strongly agree. The items have been summed and averaged to construct a continuous index where low scores signify less apathy (reference) and high scores signify more apathy.

Trust in National Government. Q14. Respondents were asked to indicate their level of trust on their national government to "act in the best interest of nature and society" on a 4 point scale: 1=do not trust at all (reference), 2=do not trust very much, 3=trust somewhat, 4=trust completely. Here employed as continuous.

Trust in Scientific Experts. Q14. Respondents were asked to indicate their level of trust on scientific experts to "act in the best interest of nature and society" on a 4 point scale: 1=do not trust at all (reference), 2=do not trust very much, 3=trust somewhat, 4=trust completely. Here employed as continuous.

Technology Optimism. Q15. Measured through respondents' agreement with two statements about the ability of science and technology to resolve climate change (e.g. "Science and technology will eventually solve our problems with climate change"). For each statement respondents were asked to indicate their level of agreement on a 4 point scale where 1=strongly disagree and 4=strongly agree. The items have been summed and averaged to construct a continuous index where low scores signify less technological optimism (reference) and high scores signify more technological optimism.

Supplementary Table 14. Descriptive statistics.

	United Kingdom			Italy			Norway			All	
	n	mean	s.d.	n	mean	s.d.	n	mean	s.d.	min	max
Age: Older	8995	3.82	1.59	9085	3.79	1.53	9085	3.66	1.61	1	6
Gender: Male	8940	1.49	0.50	9070	1.49	0.50	9020	1.53	0.50	1	2
Education: Higher	8930	1.63	0.48	9070	1.48	0.50	9005	1.63	0.48	1	2
Income: Higher	7720	1.59	0.49	7585	1.52	0.50	7650	1.77	0.42	1	2
Ideology: More Right	7755	5.07	2.17	7850	4.79	2.71	8290	5.22	2.45	0	10
Economic Liberalism: More	8830	2.91	0.55	8980	2.77	0.45	9015	2.62	0.62	1	4
CDR Lack of Familiarity	8995	1.37	0.80	9085	1.57	0.74	9085	1.23	0.88	0	2
CDR Risks Outweigh Benefits	6645	0.29	0.46	6790	0.28	0.45	6460	0.29	0.45	0	1
Climate Change: Serious	8910	7.22	2.48	9010	8.17	1.97	8960	6.52	2.54	0	10
Climate Change Apathy: More	8865	1.98	0.81	8965	1.79	0.68	8975	1.88	0.77	1	4

Trust in National Government	8625	1.28	0.84	8830	1.28	0.83	8565	1.47	0.82	0	3
Trust in Companies	8580	1.18	0.82	8845	1.15	0.83	8845	1.18	0.79	0	3
Trust in Scientific Experts	8665	2.15	0.71	8890	2.19	0.78	8820	2.19	0.79	0	3
Technology Optimism: More	8625	2.06	0.78	8795	2.09	0.68	8750	1.93	0.67	1	4
	**Brazil**			**Saudi Arabia**			**Malaysia**			**All**	
	**n**	**mean**	**s.d.**	**n**	**mean**	**s.d.**	**n**	**mean**	**s.d.**	**min**	**max**
Age: Older	9020	2.98	1.49	9045	2.63	1.32	9030	2.80	1.48	1	6
Gender: Male	9010	1.50	0.50	9010	1.52	0.50	9015	1.46	0.50	1	2
Education: Higher	9000	1.65	0.48	9000	1.60	0.49	8970	1.67	0.47	1	2
Income: Higher	8895	1.62	0.49	8605	1.50	0.50	8810	1.47	0.50	1	2
Ideology: More Right	8590	5.55	3.23	8005	6.50	2.27	8480	5.98	1.93	0	10
Economic Liberalism: More	8980	2.86	0.55	8910	2.17	0.73	8985	2.64	0.52	1	4
CDR Lack of Familiarity	9020	1.34	0.88	9045	1.15	0.93	9030	1.12	0.94	0	2
CDR Risks Outweigh Benefits	8520	0.28	0.45	7860	0.24	0.43	8365	0.35	0.48	0	1
Climate Change Serious	8975	8.79	1.65	8865	7.20	2.50	8965	7.88	1.78	0	10
Climate Change Apathy: More	9005	1.65	0.75	8885	2.06	0.86	9000	2.12	0.70	1	4
Trust in National Government	8975	1.53	0.88	8770	2.50	0.72	8870	2.01	0.78	0	3
Trust in Companies	8955	1.60	0.84	8770	2.00	0.87	8855	1.76	0.79	0	3
Trust in Scientific Experts	9000	2.48	0.66	8775	2.50	0.70	8900	2.38	0.70	0	3
Technology Optimism: More	8975	2.11	0.81	8860	2.52	0.84	8975	2.45	0.68	1	4

Note: n = number of respondents; s.d. = standard deviation.

Supplementary Figure 5. Correlation Matrix.

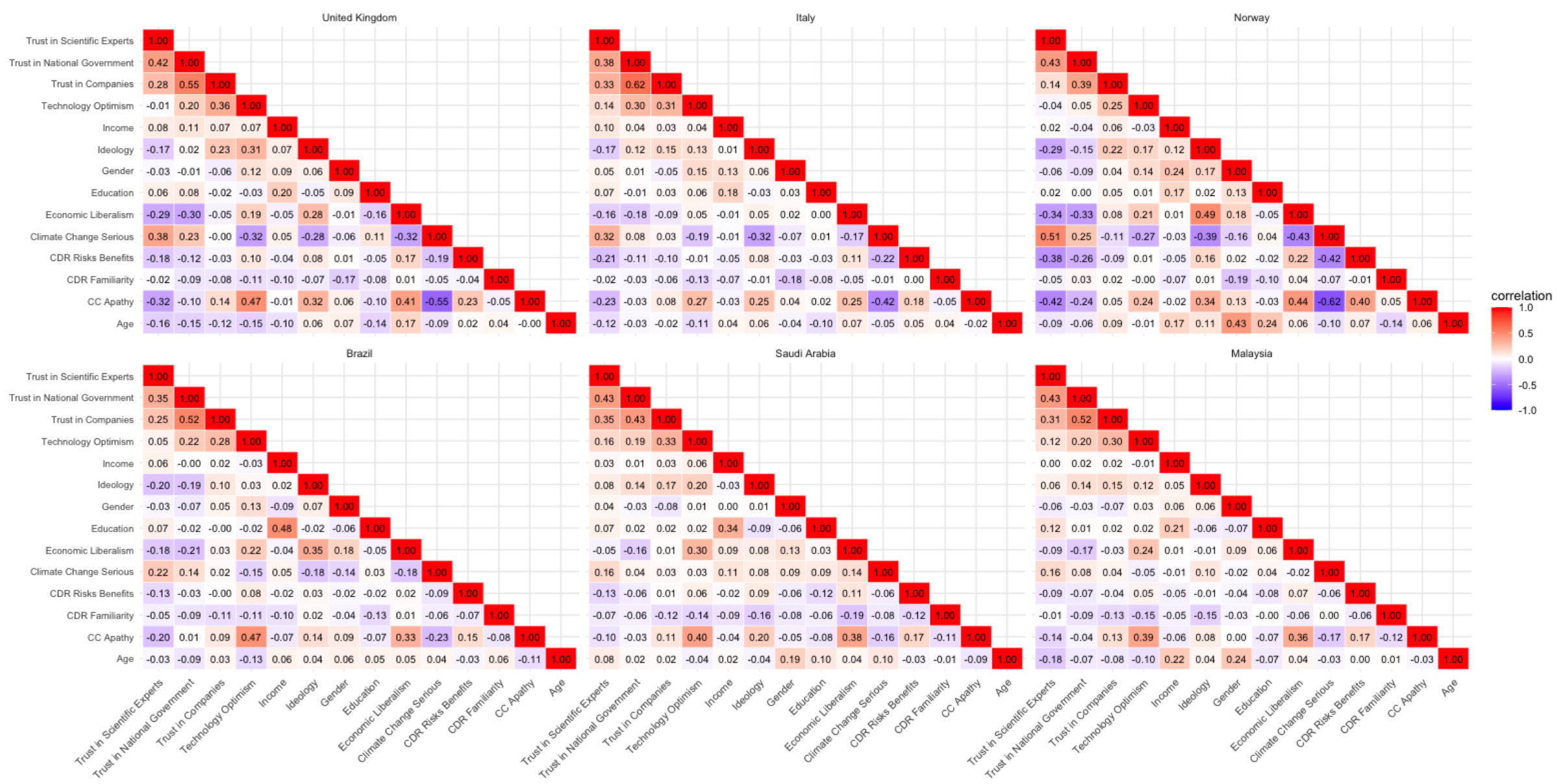

Note: Given the large number of covariates, we check for possible multicollinearity. As a first step we inspect pairwise correlations in each country, shown above as heatmap. The figure includes each correlation coefficient as a color-coded cell, with blue (or violet) representing negative correlation, red representing positive correlation, and white absence of correlation. The heatmap shows that all correlation coefficients are only moderate as none

is above .60, except for trust in companies and trust in the national government in Italy (.62) and perceived seriousness of climate change and climate change apathy in Norway (-.62).

Supplementary Table 15. Variance Inflation Factor (VIF).

	United Kingdom	Italy	Norway	Brazil	Saudi Arabia	Malaysia
Age	1.20	1.09	1.27	1.07	1.09	1.19
Gender	1.05	1.06	1.34	1.09	1.10	1.10
Education	1.11	1.07	1.06	1.31	1.17	1.10
Income	1.08	1.05	1.13	1.32	1.14	1.12
Ideology	1.29	1.16	1.49	1.25	1.13	1.08
Economic Liberalism	1.45	1.14	1.72	1.36	1.41	1.24
CDR Familiarity	1.08	1.08	1.05	1.07	1.09	1.06
CDR Risks -Benefits	1.08	1.08	1.32	1.04	1.07	1.05
Climate Change Serious	1.60	1.43	2.10	1.16	1.14	1.08
Climate Change Apathy	2.06	1.49	1.85	1.52	1.54	1.45
Trust in National Government	1.74	1.74	1.49	1.68	1.50	1.54
Trust in Companies	1.72	1.73	1.39	1.57	1.43	1.45
Trust in Scientific Experts	1.37	1.38	1.68	1.27	1.37	1.32
Technology Optimism	1.73	1.41	1.23	1.51	1.48	1.38

Note: As a second step for checking for possible multicollinearity, for each covariate we calculate the Variance Inflation Factor (VIF) through the *vif()* function of the *car* package in *R* on OLS regressions in each country. The results include values between 1.05 and 2.06 denoting only moderate multicollinearity and severe enough to require intervention.

Supplementary Table 16. Estimated average marginal component effects of governance and technical features and selected individual-level characteristics on public support for carbon removal projects (1-10 scale: 1= strongly oppose; 10=strongly support) across six countries.

Country	Attributes	Est.	s.e.	t-stat.	p-val.	stars	conf.low	conf.high	N of Vig.	N of Resp.	r2
United Kingdom	Constant	0,757	0,324	2,337	0,019	*	0,12	1,394	5115	1023	0,3
	Company	-0,289	0,061	-4,747	0	***	-0,41	-0,17	5115	1023	0,3
	Experts Consulted	1,294	0,076	16,943	0	***	1,148	1,44	5115	1023	0,3
	Locals Consulted	1,409	0,077	18,344	0	***	1,262	1,555	5115	1023	0,3
	Shared Profits	0,951	0,075	12,673	0	***	0,804	1,097	5115	1023	0,3
	No Profits	0,862	0,076	11,346	0	***	0,715	1,009	5115	1023	0,3
	Medium Removal	0,395	0,075	5,26	0	***	0,249	0,542	5115	1023	0,3
	High Removal	0,862	0,075	11,439	0	***	0,715	1,009	5115	1023	0,3
	Age: Older	-0,157	0,022	-7,138	0	***	-0,2	-0,12	5115	1023	0,3
	Gender: Male	0,3	0,064	4,722	0	***	0,174	0,425	5115	1023	0,3
	Education: Higher	-0,071	0,069	-1,039	0,299		-0,2	0,062	5115	1023	0,3
	Income: Higher	0,171	0,066	2,594	0,01	**	0,043	0,3	5115	1023	0,3
	Ideology: More Right	0,022	0,017	1,297	0,195		-0,01	0,053	5115	1023	0,3
	Economic Liberalism: More	0,087	0,07	1,23	0,219		-0,04	0,215	5115	1023	0,3
	CDR Lack of Familiarity	-0,058	0,037	-1,58	0,114		-0,13	0,015	5115	1023	0,3
	CDR Risks Outweigh the Benefits	-1,239	0,072	-17,1	0	***	-1,38	-1,1	5115	1023	0,3

	Climate Change Serious	0,258	0,017	14,974	0	***	0,227	0,29	5115	1023	0,3
	CC Apathy: More	0,093	0,055	1,693	0,091		-0,01	0,195	5115	1023	0,3
	Trust in National Government	0,247	0,048	5,183	0	***	0,154	0,341	5115	1023	0,3
	Trust in Companies	0,207	0,049	4,225	0	***	0,114	0,3	5115	1023	0,3
	Trust in Scientific Experts	0,283	0,054	5,191	0	***	0,18	0,385	5115	1023	0,3
	Technology Optimism: More	0,329	0,052	6,298	0	***	0,229	0,429	5115	1023	0,3
Italy	Constant	2,39	0,375	6,379	0	***	1,681	3,099	5155	1031	0,25
	Company	-0,095	0,062	-1,535	0,125		-0,22	0,026	5155	1031	0,25
	Experts Consulted	1,25	0,078	15,975	0	***	1,102	1,398	5155	1031	0,25
	Locals Consulted	1,417	0,078	18,131	0	***	1,269	1,565	5155	1031	0,25
	Shared Profits	0,893	0,077	11,64	0	***	0,746	1,041	5155	1031	0,25
	No Profits	0,828	0,076	10,882	0	***	0,68	0,976	5155	1031	0,25
	Medium Removal	0,543	0,077	7,01	0	***	0,394	0,692	5155	1031	0,25
	High Removal	0,909	0,076	11,906	0	***	0,761	1,056	5155	1031	0,25
	Age: Older	-0,191	0,02	-9,465	0	***	-0,23	-0,15	5155	1031	0,25
	Gender: Male	0,258	0,065	3,982	0	***	0,132	0,384	5155	1031	0,25
	Education: Higher	0,106	0,063	1,668	0,095		-0,02	0,23	5155	1031	0,25
	Income: Higher	0,269	0,063	4,232	0	***	0,144	0,393	5155	1031	0,25
	Ideology: More Right	0,03	0,013	2,375	0,018	*	0,006	0,055	5155	1031	0,25
	Economic Liberalism: More	0,076	0,079	0,962	0,336		-0,07	0,221	5155	1031	0,25
	CDR Lack of Familiarity	0,041	0,04	1,032	0,302		-0,04	0,118	5155	1031	0,25
	CDR Risks Outweigh the Benefits	-1,283	0,079	-16,2	0	***	-1,43	-1,14	5155	1031	0,25
	Climate Change Serious	0,218	0,021	10,155	0	***	0,179	0,257	5155	1031	0,25
	CC Apathy: More	-0,201	0,058	-3,452	0,001	***	-0,31	-0,09	5155	1031	0,25
	Trust in National Government	0,09	0,049	1,844	0,065		-0,01	0,184	5155	1031	0,25
	Trust in Companies	0,149	0,047	3,152	0,002	**	0,056	0,242	5155	1031	0,25
	Trust in Scientific Experts	0,069	0,049	1,395	0,163		-0,02	0,162	5155	1031	0,25
	Technology Optimism: More	0,363	0,052	6,937	0	***	0,26	0,467	5155	1031	0,25
Norway	Constant	1,256	0,322	3,904	0	***	0,633	1,879	4995	999	0,39
	Company	-0,097	0,06	-1,615	0,106		-0,22	0,021	4995	999	0,39
	Experts Consulted	1,192	0,075	15,841	0	***	1,047	1,337	4995	999	0,39
	Locals Consulted	1,194	0,075	15,882	0	***	1,049	1,339	4995	999	0,39
	Shared Profits	0,724	0,075	9,587	0	***	0,578	0,869	4995	999	0,39
	No Profits	0,688	0,074	9,362	0	***	0,544	0,833	4995	999	0,39
	Medium Removal	0,622	0,075	8,337	0	***	0,478	0,767	4995	999	0,39
	High Removal	0,96	0,074	12,928	0	***	0,815	1,106	4995	999	0,39
	Age: Older	-0,079	0,022	-3,625	0	***	-0,12	-0,04	4995	999	0,39
	Gender: Male	0,153	0,071	2,163	0,031	*	0,012	0,294	4995	999	0,39
	Education: Higher	-0,056	0,067	-0,835	0,404		-0,19	0,074	4995	999	0,39
	Income: Higher	-0,269	0,077	-3,506	0	***	-0,42	-0,12	4995	999	0,39
	Ideology: More Right	0,016	0,015	1,096	0,273		-0,01	0,045	4995	999	0,39
	Economic Liberalism: More	0,26	0,067	3,889	0	***	0,132	0,388	4995	999	0,39

	CDR Lack of Familiarity	0,038	0,034	1,122	0,262		-0,03	0,105	4995	999	0,39
	CDR Risks Outweigh the Benefits	-2,046	0,081	-25,34	0	***	-2,2	-1,89	4995	999	0,39
	Climate Change Serious	0,279	0,018	15,658	0	***	0,245	0,313	4995	999	0,39
	CC Apathy: More	-0,209	0,055	-3,812	0	***	-0,31	-0,11	4995	999	0,39
	Trust in National Government	0,269	0,049	5,541	0	***	0,18	0,358	4995	999	0,39
	Trust in Companies	0,054	0,047	1,153	0,249		-0,04	0,143	4995	999	0,39
	Trust in Scientific Experts	0,098	0,048	2,067	0,039	*	0,002	0,194	4995	999	0,39
	Technology Optimism: More	0,239	0,049	4,877	0	***	0,142	0,337	4995	999	0,39
Brazil	Constant	1,34	0,474	2,83	0,005	**	0,755	1,926	7995	1599	0,19
	Company	-0,026	0,043	-0,593	0,553		-0,14	0,085	7995	1599	0,19
	Experts Consulted	1,537	0,069	22,416	0	***	1,402	1,672	7995	1599	0,19
	Locals Consulted	1,715	0,07	24,553	0	***	1,58	1,85	7995	1599	0,19
	Shared Profits	1,211	0,064	19,049	0	***	1,076	1,345	7995	1599	0,19
	No Profits	0,883	0,062	14,203	0	***	0,748	1,018	7995	1599	0,19
	Medium Removal	0,476	0,074	6,423	0	***	0,341	0,611	7995	1599	0,19
	High Removal	0,626	0,075	8,36	0	***	0,491	0,761	7995	1599	0,19
	Age: Older	-0,12	0,033	-3,702	0	***	-0,16	-0,08	7995	1599	0,19
	Gender: Male	0,203	0,095	2,14	0,032	*	0,088	0,319	7995	1599	0,19
	Education: Higher	0,184	0,113	1,62	0,105		0,05	0,318	7995	1599	0,19
	Income: Higher	0,142	0,11	1,287	0,198		0,011	0,273	7995	1599	0,19
	Ideology: More Right	-0,002	0,016	-0,109	0,913		-0,02	0,017	7995	1599	0,19
	Economic Liberalism: More	0,109	0,1	1,083	0,279		-0,01	0,226	7995	1599	0,19
	CDR Lack of Familiarity	-0,151	0,051	-2,966	0,003	**	-0,22	-0,09	7995	1599	0,19
	CDR Risks Outweigh the Benefits	-0,594	0,114	-5,226	0	***	-0,72	-0,47	7995	1599	0,19
	Climate Change Serious	0,287	0,033	8,762	0	***	0,25	0,324	7995	1599	0,19
	CC Apathy: More	0,026	0,07	0,378	0,706		-0,06	0,116	7995	1599	0,19
	Trust in National Government	0,295	0,067	4,422	0	***	0,213	0,376	7995	1599	0,19
	Trust in Companies	0,096	0,069	1,399	0,162		0,015	0,178	7995	1599	0,19
	Trust in Scientific Experts	0,094	0,081	1,163	0,245		-0	0,189	7995	1599	0,19
	Technology Optimism: More	0,227	0,066	3,434	0,001	***	0,143	0,311	7995	1599	0,19
Saudi Arabia	Constant	2,41	0,388	6,207	0	***	1,945	2,875	6665	1333	0,15
	Company	-0,083	0,05	-1,658	0,097		-0,2	0,036	6665	1333	0,15
	Experts Consulted	1,064	0,07	15,165	0	***	0,918	1,21	6665	1333	0,15
	Locals Consulted	1,046	0,07	14,983	0	***	0,9	1,193	6665	1333	0,15
	Shared Profits	0,766	0,066	11,621	0	***	0,62	0,912	6665	1333	0,15
	No Profits	0,271	0,068	4,008	0	***	0,125	0,418	6665	1333	0,15
	Medium Removal	0,732	0,079	9,283	0	***	0,586	0,878	6665	1333	0,15
	High Removal	0,957	0,086	11,196	0	***	0,811	1,104	6665	1333	0,15
	Age: Older	-0,041	0,038	-1,066	0,286		-0,09	0,006	6665	1333	0,15
	Gender: Male	0,073	0,101	0,724	0,469		-0,05	0,199	6665	1333	0,15
	Education: Higher	-0,045	0,114	-0,394	0,694		-0,18	0,091	6665	1333	0,15

	Income: Higher	0,022	0,106	0,209	0,835		-0,11	0,15	6665	1333	0,15
	Ideology: More Right	0,103	0,026	4,012	0	***	0,075	0,131	6665	1333	0,15
	Economic Liberalism: More	0,111	0,081	1,371	0,171		0,013	0,208	6665	1333	0,15
	CDR Lack of Familiarity	-0,206	0,053	-3,929	0	***	-0,27	-0,14	6665	1333	0,15
	CDR Risks Outweigh the Benefits	-0,744	0,129	-5,778	0	***	-0,89	-0,6	6665	1333	0,15
	Climate Change Serious	0,152	0,024	6,197	0	***	0,125	0,178	6665	1333	0,15
	CC Apathy: More	0,079	0,068	1,161	0,246		-0,01	0,164	6665	1333	0,15
	Trust in National Government	0,138	0,081	1,706	0,088		0,036	0,241	6665	1333	0,15
	Trust in Companies	0,211	0,066	3,203	0,001	**	0,129	0,294	6665	1333	0,15
	Trust in Scientific Experts	0,139	0,085	1,646	0,1		0,038	0,241	6665	1333	0,15
	Technology Optimism: More	0,199	0,072	2,787	0,005	**	0,114	0,285	6665	1333	0,15
Malaysia	Constant	2,094	0,427	4,899	0	***	1,583	2,605	7580	1516	0,16
	Company	-0,109	0,042	-2,602	0,009	**	-0,21	-0	7580	1516	0,16
	Experts Consulted	1,223	0,063	19,543	0	***	1,094	1,352	7580	1516	0,16
	Locals Consulted	1,23	0,063	19,421	0	***	1,101	1,359	7580	1516	0,16
	Shared Profits	0,627	0,056	11,154	0	***	0,497	0,756	7580	1516	0,16
	No Profits	0,47	0,056	8,371	0	***	0,34	0,599	7580	1516	0,16
	Medium Removal	0,087	0,067	1,288	0,198		-0,04	0,216	7580	1516	0,16
	High Removal	0,169	0,069	2,453	0,014	*	0,04	0,298	7580	1516	0,16
	Age: Older	-0,037	0,034	-1,101	0,271		-0,08	0,003	7580	1516	0,16
	Gender: Male	0,509	0,093	5,452	0	***	0,398	0,62	7580	1516	0,16
	Education: Higher	0,239	0,105	2,266	0,023	*	0,119	0,358	7580	1516	0,16
	Income: Higher	0,17	0,094	1,819	0,069		0,058	0,282	7580	1516	0,16
	Ideology: More Right	0,095	0,026	3,647	0	***	0,067	0,123	7580	1516	0,16
	Economic Liberalism: More	-0,128	0,099	-1,293	0,196		-0,24	-0,02	7580	1516	0,16
	CDR Lack of Familiarity	-0,061	0,048	-1,267	0,205		-0,12	-0	7580	1516	0,16
	CDR Risks Outweigh the Benefits	-0,825	0,101	-8,153	0	***	-0,94	-0,71	7580	1516	0,16
	Climate Change Serious	0,204	0,028	7,201	0	***	0,172	0,235	7580	1516	0,16
	CC Apathy: More	0,057	0,078	0,737	0,461		-0,03	0,147	7580	1516	0,16
	Trust in National Government	0,03	0,073	0,415	0,678		-0,06	0,116	7580	1516	0,16
	Trust in Companies	0,263	0,067	3,908	0	***	0,182	0,343	7580	1516	0,16
	Trust in Scientific Experts	0,154	0,073	2,11	0,035	*	0,066	0,242	7580	1516	0,16
	Technology Optimism: More	0,261	0,081	3,221	0,001	**	0,17	0,352	7580	1516	0,16

Note: N=7,501 respondents, due to the exclusion from the analysis of observations with missing values in at least one of the individual characteristics. Estimates from OLS regressions with clustered standard errors at the individual level (in parentheses). Reference levels: regional government, no consultation, private profits, small removal, age: youngest age group, gender: female, education: lower, income: lower, ideology: more left, economic liberalism: less, familiarity with CDR, CDR benefits overweigh risks, climate change not serious at all, climate change apathy less, trust in national government: no trust at all, trust in companies: no trust at all, trust in scientific experts: no trust at all; technology optimism: least. Statistical inference is based on two-sided t-tests against a zero-null hypothesis. No adjustment for multiple comparisons was applied. Est. = estimates; s.e. = standard errors; t-stat. = t-statistics; p-val. = p-values; stars = "***" ~ $p < 0.001$, "**" ~ $p < 0.01$, "*" ~ $p < 0.05$; conf.low and conf.high delimit the confidence intervals; N of Vig. = number of vignettes (country-level); N of Resp. = number of respondents (country-level); r2 = r squared.

IX. Quotas Information by Country (percentages) – Whole Sample

Supplementary Table 17. Descriptive statistics of quota variables by country, in percentages.

Variable	Value	United Kingdom	Italy	Norway	Brazil	Saudi Arabia	Malaysia
Gender	Female	51	51	47	50	48	53
	Male	48	49	52	49	52	46
	Non-binary	1	0	0	0	0	0
	Other	0	0	0	0	0	0
	Prefer not to say	0	0	0	0	0	0
Age	18-24	11	10	13	18	24	23
	25-34	11	12	14	26	27	26
	35-44	18	19	16	21	21	21
	45-54	22	24	23	17	17	12
	55-64	20	19	18	11	10	15
	65-75	18	16	16	7	1	4
Education	Lower	36	52	37	35	40	33
	Higher	63	48	62	65	60	67
	No Answer	1	0	1	0	0	1
Not criteria for quotas							
Area of living	A big city	19	24	24	73	84	33
	Suburbs or outskirts of a big city	24	7	16	5	7	31
	Small / Medium Town	41	49	34	20	6	23
	A country village	13	17	14	1	2	11
	A farm or home in the countryside	2	2	11	0	0	2
	Other	0	1	1	0	0	0
	Prefer not to say	0	0	0	0	0	0
Income	Lower	35	40	19	38	47	52
	Higher	51	43	65	61	48	46
	No Answer	14	17	16	1	5	2

Note: N=10,852 respondents. Percentages of respondents in each category for each country. For the country-specific formulations of education and income, refer to the questionnaire at the end of this document.

X. Quotas Information by Country (percentages) – CDR Technologies Sub-Samples

Supplementary Table 18. Descriptive statistics of quota variables by country within CDR sub-samples, in percentages.

DACCS

Variable	Value	United Kingdom	Italy	Norway	Brazil	Saudi Arabia	Malaysia
Gender	Female	51	51	47	50	48	53
	Male	48	49	52	50	52	47
	Non-binary	1	0	1	0	0	
	Other	0	0	0	0	0	0
	Prefer not to say	0	0	0	0	0	0

Age	18-24	12	10	13	18	24	23
	25-34	11	12	14	26	27	26
	35-44	17	19	16	21	21	21
	45-54	22	24	23	17	17	12
	55-64	20	19	19	11	10	14
	65-75	18	16	16	7	0	5
Education	Lower	36	52	37	35	40	33
	Higher	63	48	62	65	59	66
	No Answer	1	0	1	0	1	1
Not criteria for quotas							
Area of living	A big city	20	23	23	74	82	32
	Suburbs or outskirts of a big city	25	6	15	4	8	30
	Small / Medium Town	40	50	35	21	6	27
	A country village	12	19	15	1	3	9
	A farm or home in the countryside	1	2	10	0	0	2
	Other	0	1	1	0	0	0
	Prefer not to say	0	0	0	0	0	0
Income	Lower	37	38	21	34	49	55
	Higher	48	46	64	64	48	43
	No Answer	15	16	16	1	3	2
**BECCS**							
**Variable**	**Value**	**United Kingdom**	**Italy**	**Norway**	**Brazil**	**Saudi Arabia**	**Malaysia**
Gender	Female	51	51	47	50	47	54
	Male	48	49	52	50	52	46
	Non-binary	0	0	0	0	0	
	Other	0	0	0	0	0	0
	Prefer not to say	0	0	0	0	0	0
Age	18-24	11	10	13	18	25	23
	25-34	11	12	14	26	28	26
	35-44	18	19	16	21	21	20
	45-54	22	24	23	17	16	11
	55-64	20	20	18	11	9	16
	65-75	18	16	16	7	1	3
Education	Lower	36	52	37	35	40	32
	Higher	63	48	62	65	60	67
	No Answer	1	0	1		0	1
Not criteria for quotas							
Area of living	A big city	18	26	23	72	85	34
	Suburbs or outskirts of a big city	22	6	16	5	7	33
	Small / Medium Town	43	49	36	21	7	20
	A country village	14	16	13	1	1	12

	A farm or home in the countryside	3	1	10	0	0	1
	Other	0	1	1	0	0	0
	Prefer not to say	0	0	0	0	0	0
Income	Lower	35	41	18	40	47	49
	Higher	52	43	65	59	49	49
	No Answer	13	16	17	1	4	1

**ERW**							
**Variable**	**Value**	**United Kingdom**	**Italy**	**Norway**	**Brazil**	**Saudi Arabia**	**Malaysia**
Gender	Female	51	51	47	51	48	54
	Male	48	48	52	49	52	46
	Non-binary	1	0	0	0	0	0
	Other	0	0	0	0	0	
	Prefer not to say	0	0	0	0	0	0
Age	18-24	11	10	13	18	24	23
	25-34	11	12	14	26	27	26
	35-44	18	19	16	21	21	21
	45-54	22	24	23	17	17	12
	55-64	20	19	18	11	10	15
	65-75	18	16	16	7	1	4
Education	Lower	36	52	37	35	39	33
	Higher	63	48	63	65	60	67
	No Answer	1		0	0	0	1
Not criteria for quotas							
Area of living	A big city	18	24	26	73	85	33
	Suburbs or outskirts of a big city	25	8	16	7	7	30
	Small / Medium Town	42	49	29	19	6	23
	A country village	13	17	15	0	2	11
	A farm or home in the countryside	2	1	13	0	0	2
	Other	1	0	1	0	0	0
	Prefer not to say	0	0	0	0	0	0
Income	Lower	34	42	19	39	46	52
	Higher	52	41	67	60	47	45
	No Answer	14	17	14	1	7	3

Note: N=10,852 respondents. Percentages of respondents in each category for each CDR technology subsample in country. For the country-specific formulations of Education and Income, refer to the questionnaire at the end of this document.

XI. Robustness check excluding respondents selecting the same value across vignettes

Supplementary Table 19. Count of respondents selecting the same value across all vignette and corresponding values, in percentages.

**Value**	**United Kingdom**	**Italy**	**Norway**	**Brazil**	**Saudi Arabia**	**Malaysia**
1	58	35	107	8	15	14

2	1	2	6	1	0	1
3	1	1	4	0	3	0
4	1	4	7	0	2	2
5	64	24	49	16	20	38
6	9	21	7	2	3	8
7	7	4	7	6	0	18
8	13	19	6	11	7	31
9	6	10	1	12	8	23
10	38	72	38	196	197	105

N=1,369 respondents.

Supplementary Table 20. Estimated average marginal component effects of governance and technical features on public support for carbon removal projects (1-10 scale: 1= strongly oppose; 10=strongly support) across six countries, after excluding respondents who selected the same value across all vignettes.

Country	Attributes	Est.	s.e.	t-stat.	p-val.	stars	conf.low	conf.high	N of Vig.	N of Resp.	r2
United Kingdom	Constant	4,033	0,083	48,553	0	***	3,895	4,172	8005	1601	0,145
	Company	-0,28	0,038	-7,394	0	***	-0,38	-0,18	8005	1601	0,145
	Experts Consulted	1,388	0,056	24,861	0	***	1,269	1,508	8005	1601	0,145
	Locals Consulted	1,522	0,057	26,782	0	***	1,402	1,641	8005	1601	0,145
	Shared Profits	1,05	0,052	20,162	0	***	0,931	1,17	8005	1601	0,145
	No Profits	0,926	0,054	17,135	0	***	0,806	1,046	8005	1601	0,145
	Medium Removal	0,493	0,065	7,618	0	***	0,374	0,613	8005	1601	0,145
	High Removal	0,864	0,065	13,269	0	***	0,744	0,984	8005	1601	0,145
Italy	Constant	4,274	0,08	53,2	0	***	4,138	4,409	8125	1625	0,158
	Company	-0,125	0,039	-3,226	0,001	**	-0,22	-0,03	8125	1625	0,158
	Experts Consulted	1,518	0,059	25,58	0	***	1,4	1,636	8125	1625	0,158
	Locals Consulted	1,608	0,058	27,753	0	***	1,491	1,726	8125	1625	0,158
	Shared Profits	0,972	0,054	17,946	0	***	0,854	1,09	8125	1625	0,158
	No Profits	0,909	0,054	16,851	0	***	0,791	1,027	8125	1625	0,158
	Medium Removal	0,631	0,063	9,985	0	***	0,513	0,749	8125	1625	0,158
	High Removal	0,973	0,065	14,954	0	***	0,855	1,091	8125	1625	0,158
Norway	Constant	3,529	0,079	44,424	0	***	3,388	3,671	7925	1585	0,13
	Company	-0,13	0,042	-3,125	0,002	**	-0,23	-0,03	7925	1585	0,13
	Experts Consulted	1,439	0,055	26,253	0	***	1,317	1,562	7925	1585	0,13
	Locals Consulted	1,474	0,056	26,107	0	***	1,351	1,597	7925	1585	0,13
	Shared Profits	0,744	0,053	13,907	0	***	0,621	0,867	7925	1585	0,13
	No Profits	0,748	0,052	14,467	0	***	0,625	0,871	7925	1585	0,13
	Medium Removal	0,61	0,068	8,942	0	***	0,487	0,733	7925	1585	0,13
	High Removal	1,008	0,069	14,68	0	***	0,886	1,131	7925	1585	0,13
Brazil	Constant	4,46	0,099	45,154	0	***	4,299	4,621	7760	1552	0,156
	Company	-0,033	0,047	-0,698	0,485		-0,15	0,081	7760	1552	0,156
	Experts Consulted	1,747	0,073	24,068	0	***	1,608	1,887	7760	1552	0,156

	Locals Consulted	1,931	0,073	26,429	0	***	1,792	2,07	7760	1552	0,156
	Shared Profits	1,434	0,067	21,444	0	***	1,294	1,573	7760	1552	0,156
	No Profits	1,056	0,066	16,101	0	***	0,917	1,196	7760	1552	0,156
	Medium Removal	0,529	0,077	6,904	0	***	0,39	0,669	7760	1552	0,156
	High Removal	0,66	0,077	8,575	0	***	0,521	0,8	7760	1552	0,156
Saudi Arabia	Constant	5,056	0,099	51,189	0	***	4,895	5,216	7770	1554	0,097
	Company	-0,048	0,049	-0,969	0,333		-0,16	0,066	7770	1554	0,097
	Experts Consulted	1,224	0,068	17,874	0	***	1,085	1,363	7770	1554	0,097
	Locals Consulted	1,189	0,069	17,13	0	***	1,05	1,329	7770	1554	0,097
	Shared Profits	0,876	0,065	13,562	0	***	0,737	1,015	7770	1554	0,097
	No Profits	0,391	0,066	5,922	0	***	0,251	0,53	7770	1554	0,097
	Medium Removal	0,881	0,077	11,401	0	***	0,742	1,021	7770	1554	0,097
	High Removal	1,158	0,08	14,399	0	***	1,019	1,297	7770	1554	0,097
Malaysia	Constant	5,147	0,089	57,852	0	***	4,997	5,297	7830	1566	0,085
	Company	-0,1	0,043	-2,313	0,021	*	-0,21	0,006	7830	1566	0,085
	Experts Consulted	1,353	0,065	20,947	0	***	1,223	1,482	7830	1566	0,085
	Locals Consulted	1,356	0,065	20,95	0	***	1,227	1,486	7830	1566	0,085
	Shared Profits	0,777	0,058	13,379	0	***	0,647	0,906	7830	1566	0,085
	No Profits	0,596	0,057	10,402	0	***	0,466	0,726	7830	1566	0,085
	Medium Removal	0,182	0,068	2,679	0,007	**	0,053	0,312	7830	1566	0,085
	High Removal	0,259	0,069	3,743	0	***	0,129	0,389	7830	1566	0,085

Note: N=9,483 respondents, due to the exclusion from the analysis of respondents who selected the same value across all vignettes. Estimates from OLS regressions with clustered standard errors at the individual level (in parentheses). Reference levels: regional government, no consultation, private profits, small removal. Statistical inference is based on two-sided t-tests against a zero-null hypothesis. No adjustment for multiple comparisons was applied. Est. = estimates; s.e. = standard errors; t-stat. = t-statistics; p-val. = p-values; stars = "***" ~ $p < 0.001$, "**" ~ $p < 0.01$, "*" ~ $p < 0.05$; conf.low and conf.high delimit the confidence intervals; N of Vig. = number of vignettes (country-level); N of Resp. = number of respondents (country-level); r2 = r squared. For full sample results see Supplementary Table 8.

XII. Questions used in the analysis: formulation

Supplementary Questionnaire 1.

In this last section, we give the complete question wording (English language) of the parts of the survey used in the analysis in the order they were shown to the respondents. In addition, we include attention checks, the opening and closure of the survey (i.e. consent and debriefing).

Consent

Thank you for your interest in participating in this study.

Research Description: The purpose of the study is to investigate public attitudes towards climate change and technologies that could help to address it. The study is coordinated by Aarhus University (Denmark) and funded by the European Union as part of the project UPTAKE (project no. 101081521). Recruitment is carried out by Norstat Denmark.

Your contribution: In this questionnaire, we will ask you some standard background questions to get to know you better. Furthermore, we will provide you with descriptions of some climate innovations and will ask for your opinion on these. Your opinion is very valuable, even if you

are not very familiar with the topics we ask about. There are no correct or incorrect answers, only your personal opinion matters.

Filling in the questionnaire will take about 13 minutes.

Withdrawal of consent: Your participation in the survey is greatly appreciated, but you can withdraw your consent at any time by closing the window and exiting the survey. All incomplete surveys will be considered to have withdrawn consent and will be automatically deleted. After completion and submission of the answers, consent can no longer be withdrawn.

Processing of your data

The data you provide will be used exclusively for scientific research and will be handled in accordance with the European General Data Protection Regulation (Article 6(1)(e)) and the Danish Data Protection Act (section 10(1)). After data collection is complete, all data will be first pseudonymized by Norstat Denmark and after quality checks (approximately two months from now), data will be fully anonymized. This final anonymous dataset will be used only for scientific research and made permanently publicly available for transparency. In this dataset, you or your responses will **not** be personally identifiable. The results of this study will be published in a public report and one or more academic articles.

The Data Controller for this project is Aarhus University (Nordre Ringgade 1, 8000 Aarhus C, Denmark, CVR: 31119103).

Contact Information: If you wish to contact the researchers behind the study, you can do so by emailing the researcher responsible for this survey Livia Fritz at livia.fritz@btech.au.dk, or the Data Protection Officer at Aarhus University at dpo@au.dk.

Concerns and complaints: If you wish to complain about the processing of your personal data, you can do so by contacting the supervisory authority: The Danish Data Protection Agency dt@datatilsynet.dk (Carl Jacobsens Vej 35, 2500 Valby, Denmark).

If you consent to the above, select "Consent" to start the survey.

- Consent
- Do Not Consent (--> redirect to end screen: Thank you for your time! Your answers will **not** be registered. You can now close this window.)

Age

Q1. What is your age? [numeric open field]

Gender

Q2. What is your gender?

1. Female
2. Male
3. Non-binary
4. Other
5. Prefer not to say

Education

Q3. What is the highest level of education you have completed?

United Kingdom	Italy	Norway	Brazil	Saudi Arabia	Malaysia
Below GCSE	Less than secondary school	Primary or lower secondary school	Primary or lower secondary education	Primary or lower secondary education	Primary or lower secondary education

GSCE	Upper secondary school	Upper secondary school	High school	Secondary school	Upper Secondary school
Further education (not university degree)	Post-secondary vocational training	Vocational College Degree	Post-high school vocational training	Vocational / Technical Certificate	Vocational College Degree
Undergraduate Degree	Bachelors degree	Other vocational training	Associate degree *	Diploma / Associate diploma	Other vocational training
Postgraduate Masters	Postgraduate Diploma	Bachelor degree	Bachelor degree	Bachelor's degree	Bachelor's degree
Postgraduate PhD	Master's degree	Masters degree	Masters or professional degree	Master's Degree	Master's degree
	Doctorate degree (PhD)	Doctorate degree (PhD)	Doctorate degree (PhD)	Doctorate degree (PhD)	Doctorate degree (PhD)

Note: Gray shading corresponds to recoding into "lower education".

Region of residence

Q4. Which region do you live in?
Country-specific regions for quotas.

Introduction to the vignettes – for all

Using coal, oil, and gas for energy or transportation, cutting down forests, and raising animals emits **greenhouse gases like carbon dioxide (CO_2)** into the air. These gases **trap heat, making the Earth warmer**.

This disrupts nature, leading to **more extreme weather** like heat waves, wildfires, and floods. Scientists warn that if we don't reduce greenhouse gas emissions, these events will become more frequent and severe.

Cutting emissions is important. In addition, there is the idea to **take out some of the CO_2** that we have already emitted into the air and **store it for thousands of years**.

We will now show you one method to remove and store CO_2. Please read the next section attentively so you can answer the questions that follow.

Creation of sub-samples and information provision

- Randomly assign participants into Group 1, 2 and 3 on least fill criteria for age, gender and region.
- Show for 20 seconds before allowing to continue

Group 1: Direct Air Capture with Carbon Storage DACCS.

CO_2 can be filtered from the air.
Large fans pull air into filters that separate the CO_2 from the air using chemical reactions.

These filters are **expensive** to build and maintain. Furthermore, filtering the CO_2 **uses a lot of energy.** This energy must come from renewable sources, to avoid additional CO_2 emissions.
The **captured CO_2** can be **transported** for example via pipelines to **deep underground storage sites** like old oil and gas wells or newly established storage sites. It is projected to **remain there for thousands of years**.
CO_2 transport and storage are designed to be safe, but in **rare cases leaks** could pose risks to nearby people, animals, and plants.
Regular monitoring and safety measures are necessary to prevent this.
This method to remove CO_2 from the air is called **Direct Air Carbon Capture and Storage (DACCS)**.

[For an illustration shown to participants alongside the text see Losi et al. 2026]

Group 2: Bioenergy with Carbon Capture and Storage – BECCS.

Plants absorb CO_2 from the air as they grow. When they are burnt at **power plants** to produce electricity and heat for local communities (so-called 'bioenergy'), the CO_2 is released back into the air.
To prevent this, the **CO_2 can be filtered from the exhaust streams of the power plants** using chemical reactions.
Growing the plants for bioenergy production **requires** a lot of **land and water**. This can compete with food production or nature conservation.
The **captured CO_2** can be **transported** for example via pipelines to **deep underground storage sites** like old oil and gas wells or newly established storage sites. It is projected to **remain there for thousands of years**.
CO_2 transport and storage are designed to be safe, but in **rare cases leaks** could pose risks to nearby people, animals, and plants.
Regular monitoring and safety measures are necessary to prevent this.
This method to remove CO_2 from the air is called **Bioenergy with Carbon Capture and Storage (BECCS).**

[For an illustration shown to participants alongside the text see Losi et al. 2026]

Group 3: Enhanced Rock Weathering – ERW.

When **rocks, like basalt**, are exposed to water (like rain), they chemically react with it in a process called weathering. This **process removes CO_2 from the air** and locks it away in soils or dissolved in water. Over time, it can be transported via rivers to the oceans, where it is projected to **remain stored for thousands of years.**
This process can be sped up by **finely crushing the rocks**, before **spreading** them on farmland.
Besides capturing CO_2, the crushed rocks can improve soil health and **boost crop yields**, potentially reducing the need for chemical fertilizers.
However, **large amounts of rocks are needed**, requiring new quarries. Furthermore, a **lot of energy** is needed for crushing the rocks. This energy must come from renewable sources, to avoid additional CO_2 emissions.
Some rocks contain toxic metals and should not be used. The fine rock dust can harm workers on the field.
Regular monitoring and safety measures are necessary to prevent this.
This method to remove CO_2 from the air is called **Enhanced Rock Weathering (ERW).**

[For an illustration shown to participants alongside the text see Losi et al. 2026]

Information on profit management – for all

Projects that remove CO_2 from the air can be administered in different ways, either to generate profits or as a public service. In both cases, the costs to run the projects are the same.

In one case, companies, governments and individuals who would like to compensate for their emissions **pay for <technology abbreviation> to remove** some CO_2 from the air**.** These payments cover the costs of the projects and **generate profits**.

Alternatively, such projects can be run in a **not-for-profit** manner and remove CO_2 from the air as a public service **without generating extra profits**.

You will now read about 5 different local <DACCS/BECCS/ERW full name and abbreviation> projects and we'll ask you what you think about them.

Experiment

Imagine that…

- ➢ a **company** starts a <DACCS/BECCS/ERW only abbreviation> project in <country / the UK>.
- ➢ a **regional government** starts a <DACCS/BECCS/ERW only abbreviation> project in <country>.

- This project is permitted by the authorities **without asking the opinion of** the affected local population **or the advice** of experts
- This project is permitted by the authorities **after listening to** the opinions of the **affected local population**
- This project is permitted by the authorities **after listening to** the advice of the **scientific experts**

- ▪ The **profits will go to the [company / regional government**]
- ▪ The **profits will be shared** between the [company / regional government] and the affected local population, who will receive direct payments**.**
- ▪ The project is run in a not-for-profit manner, so it **does not generate profits**.

- This project can remove a **small amount of CO_2** comparable to the average annual emissions of 5 000 mid-range passenger cars.
- This project can remove a **moderate amount of CO_2** comparable to the average annual emissions of 10 000 mid-range passenger cars.
- This project can remove a **large amount of CO_2** comparable to the average annual emissions of 15 000 mid-range passenger cars.

Vignettes assessment – after each vignette

Q5. How much would you oppose or support this project?

1 means that you strongly oppose the project and 10 means that you strongly support the project.

Strongly oppose									Strongly support
1	2	3	4	5	6	7	8	9	10

Q6. How unfair or fair do you think this project is to the local population affected by it?
1 means that you consider the project very unfair and 10 means that you consider the project very fair.

Very unfair									Very fair
1	2	3	4	5	6	7	8	9	10

Attention check

Q7. Every day I swim across the Atlantic Ocean to get to work.
1. Strongly agree
2. Agree
3. Disagree *CORRECT*
4. Strongly Disagree *CORRECT*

Engagement

Q8. When starting a new <DACCS/BECCS/ERW only abbreviation> project, there are various decisions to take and different ways to involve the local population. When it comes to the planning of such a project, what level of involvement of the local population do you think is fair for each of the following decisions?

	No involvement at all	Local population is informed	Local population is asked to comment	Local population votes	Don't know
1 Decisions on whether the project is permitted	1	2	3	4	88
2 Decisions on the location where to implement the project	1	2	3	4	88
3 Decisions on how to distribute profits	1	2	3	4	88

Familiarity with specific CDR technology

Q9. Had you ever heard of <DACCS/BECCS/ERW only abbreviation> before this survey?

1. Yes, I had heard a lot about this.
2. Yes, I had heard a little about this.
3. No, I had not heard about this.
4. No, I had not heard about this, but I have heard of other carbon removal methods before.

Perceptions of risks of specific CDR method

Q10. Please consider the risks and benefits of <DACCS/BECCS/ERW only abbreviation>. Do you think that the risks outweigh the benefits or that the benefits outweigh the risks?

1. risks strongly outweigh the benefits
2. risks slightly outweigh the benefits
3. benefits slightly outweigh the risks
4. benefits strongly outweigh the risks
88. Don't know

General technology support

Q11. IF DACCS or BECCS: In general, how strongly would you support or oppose the construction of the following facilities within 7 miles [if country NOT UK use 10 km] from your home?

Q11. IF ERW: In general, how strongly would you support or oppose the construction of the following facilities or the use of the following methods within 7 miles [if country NOT UK use 10 km] from your home?

If you live in a larger city, please think of the surrounding area.

	Strongly oppose	Oppose	Slightly oppose	Slightly support	Support	Strongly support	Don't know
1 Wind turbines to produce energy	1	2	3	4	5	6	88
2 Solar panels to produce energy	1	2	3	4	5	6	88
3 Nuclear power plant to produce energy	1	2	3	4	5	6	88
4 Underground storage site for nuclear waste	1	2	3	4	5	6	88
5 [IF DACCS or BECCS] Underground storage site for CO_2	1	2	3	4	5	6	88
6 [IF DACCS] Plant to directly capture CO_2 from the air for DACCS	1	2	3	4	5	6	88
7 [IF BECCS] Bioenergy plant with CO_2 capture for BECCS	1	2	3	4	5	6	88
8 [IF ERW] Stone-crushing facility for enhanced rock weathering	1	2	3	4	5	6	88
9 [IF ERW] Spreading crushed rock on farmland for enhanced rock weathering	1	2	3	4	5	6	88

Climate change as serious problem

Q12. On a scale from 0 to 10, how serious of a problem do you think climate change is at the moment?
0 means that you consider climate change to not be a serious problem at all, and 10 means that you consider climate change to be a very serious problem.

Not at all serious										Very serious	Don't know
0	1	2	3	4	5	6	7	8	9	10	88

Climate change apathy

Q13. People have different opinions about taking action regarding climate change. How much do you agree or disagree with the following statements?

	Strongly disagree	Somewhat disagree	Somewhat agree	Strongly agree	Don't know
1 There is no point in me doing anything about climate change because nobody else is	1	2	3	4	88
2 It is already too late to do anything about climate change	1	2	3	4	88
3 Climate change is so complicated, that there is very little institutions can do about it	1	2	3	4	88
4 It is not in humans' power to do anything about climate change	1	2	3	4	88

Trust in vignette actors

Q14. How much do you trust the following actors to act in the best interest of nature and society?

	Do not trust at all	Do not trust very much	Trust somewhat	Trust completely	Don't know
1 Companies and industry	1	2	3	4	88
2 Scientific experts	1	2	3	4	88
3 National and regional governments	1	2	3	4	88
4 Local communities	1	2	3	4	88

Science Optimism

Q15. The following are statements that people have made about technology and climate change. For each statement, please indicate how much you agree or disagree.

	Strongly disagree	Somewhat disagree	Somewhat agree	Strongly agree	Don't know
1 Science and technology will eventually solve our problems with climate change	1	2	3	4	88
2 Thanks to technological progress, we do not need to change our behaviour at all	1	2	3	4	88

Attention check

Q16. Which one of the items listed below is a colour?

1. Sandwich
2. Table
3. Red *CORRECT*
4. Tree

Screen out criteria:
If answers correct proceed to next question
If answers wrong, show again with this additional text on top:
You gave the wrong answer to this question. Please, try answering this question again.

If answers correct proceed to next question
If answers wrong a second time, show A2 again with this additional text on top:
You gave the wrong answer to this question. Please, try reading the question carefully and answer this question again.

If answers correct proceed to next question
If answers wrong a third time, screen out.

Left-right scale

Q17. In politics people sometimes talk of 'left' and 'right'. Where would you place yourself on this scale, where 0 means furthest to the left and 10 means furthest to the right?

Furthest to the left										Furthest to the right	Don't know / Prefer not to say
0	1	2	3	4	5	6	7	8	9	10	88

Economic Liberalism I

Q18a. How strongly do you agree or disagree with each of these statements?

	Strongly disagree	Somewhat disagree	Somewhat agree	Strongly agree	Don't know
1 Government is usually wasteful and inefficient	1	2	3	4	88
2 Businesses and farms should operate without governmental subsidies	1	2	3	4	88
3 Government regulation of business usually does more harm than good.	1	2	3	4	88
4 People are better off with free trade than with tariffs	1	2	3	4	88

Economic Liberalism II

Q18b. People in our society often disagree about how much to let individuals make decisions for themselves. How strongly do you agree or disagree with each of these statements?

	Strongly disagree	Somewhat disagree	Somewhat agree	Strongly agree	Don't know
1 The government interferes far too much in our everyday lives	1	2	3	4	88
2 The government should do more to advance society's goals, even if that means limiting the freedom and choices of individuals	1	2	3	4	88
3 It's not the government's business to try to protect people from themselves	1	2	3	4	88

Area of living

Q19. How would you describe the area you live in?

1. A big city

2. Suburbs or outskirts of a big city
3. Small / Medium Town
4. A country village
5. A farm or home in the countryside
6. Other
99. Prefer not to say

Income

Q20. Country specific gross income (after-tax) ranges.

United Kingdom	Italy	Norway	Brazil	Saudi Arabia	Malaysia
£1,499 or less	€1,499 or less	24.999 NOK or less	R$1,499 or less	SR 3,999 or less	RM 1,999 or less
£1,500 - £1,999	€1,500 - €1,999	25.000 NOK - 39.999 NOK	R$1,500 - R$2,999	SR 4,000 - SR 7,999	RM 2,000 - RM 3,999
£2,000 - £2,499	€2,000 - €2,499	40.000 NOK - 69.999 NOK	R$3,000 - R$4,999	SR 8,000 - SR 11,999	RM 4,000 - RM 5,999
£2,500 - £2,999	€2,500 - €2,999	70.000 NOK - 99.999 NOK	R$5,000 - R$9,999	SR 12,000 - SR 18,999	RM 6,000 - RM 7,999
£3,000 - £3,999	€3,000 - €3,999	100.000 NOK - 124.999 NOK	R$10,000 - R$19,999	SR 20,000 - SR 49,999	RM 8,000 - RM 10,999
£4,000 - £4,999	€4,000 - €4,999	125.000 NOK or more	R$20,000 - R$29,999	SR 50,000 or more	RM 11,000 - RM 19,999
£5,000 or more	€5,000 or more	-	R$30,000 or more	-	RM 20,000 or more

Note: All countries included the options "prefer not to say" and "don't know". Gray shading corresponds to recoding into "lower income".

Final debriefing and end

Thank you very much for your participation!

This study is an important part of the [*ANONYMIZED*] project [*ANONYMIZED*]which focuses on [*ANONYMIZED*]. With this survey we aimed to investigate public perceptions of Carbon Dioxide Removal technologies and how these vary in different situations.

To investigate this, after a description of a method to remove CO_2 from the air we have shown you different projects with varying degrees of distribution of responsibilities, governance and benefits. We are interested to know which of these characteristics are more or less relevant when individuals are asked to evaluate Carbon Dioxide Removal technologies.

To complete the survey and submit your answers you can select "Submit". After completion and submission of the answers, consent can no longer be withdrawn. If you wish to withdraw your consent now, you can do so by closing this page and leaving the website.

To follow the developments of the [*ANONYMIZED*] project and the results of this study, you may follow us here [*ANONYMIZED*].

To submit your answers, please select "Submit". If you want to withdraw consent and not submit your answers, simply close this window.

- Submit (--> end screen / Thank you for your time! Your answers have been registered. You can now close this window)

XIII. References

Arnstein, S. R. A ladder of citizen participation. J. Am. Inst. Plann. 35, 216–224 (1969)

Carbon Gap Policy Tracker. Carbon Removal Policy in Italy. https://tracker.carbongap.org/regional-analysis/national/italy/

Cox, Emily, Robin Lim, Elspeth Spence, Melissa Payne, David Beerling, and Nick Pidgeon. "Question-Led Innovation: Public priorities for enhanced weathering research in Malaysia." Environmental Science & Policy 163 (2025): 103977.

Fritz, L., Baum, C. M., Low, S. & Sovacool, B. K. Public engagement for inclusive and sustainable governance of climate interventions. Nat. Commun. 15, 4168 (2024).

Global CCS Institute. Global Status of CCS 2025. https://www.globalccsinstitute.com/wp-content/uploads/2025/10/Global-Status-of-CCS-2025-report-9-October.pdf

Gidden, M.J., Joshi, S., Armitage, J.J., Christ, A.B., Boettcher, M., Brutschin, E., Köberle, A.C., Riahi, K., Schellnhuber, H.J., Schleussner, C.F. and Rogelj, J., 2025. A prudent planetary limit for geologic carbon storage. Nature, 645(8079), pp.124-132. https://doi.org/10.1038/s41586-025-09423-y

Lamb, W.F., Reynolds, C.C., Geden, O., Smith, S.M., Vaughan, N.E., Nemet, G., Lebling, K., Edwards, M., Minx, J.C., Gidden, M.J. Carbon dioxide removal in the G20 pledges: limited and lacking credibility. A State of Carbon Dioxide Removal Insight Report. https://www.stateofcdr.org, doi: https://doi.org/10.17605/OSF.IO/X8U9M (2025).

Losi L, Fritz L, Merk C, Boldrini M, Bosetti V. UPTAKE Survey Experiment. Published online 2026. https://doi.org/10.17605/OSF.IO/2XGC6

Limenet. Website (accessed 06.03.2026) https://limenet.tech/en/technology/

Newell, P., Srivastava, S., Naess, L. O., Torres Contreras, G. A., & Price, R. (2021). Toward transformative climate justice: An emerging research agenda. Wiley Interdisciplinary Reviews: Climate Change, 12(6), e733.

Petronas. Media release (accessed 06.03.2026). https://www.petronas.com/media/media-releases/petronas-ccs-ventures-sdn-bhd-receives-malaysias-first-offshore-assessment

Smith, A., Contreras, G. A. T., Brisbois, M. C., Lacey-Barnacle, M., & Sovacool, B. K. (2023). Inclusive innovation in just transitions: the case of smart local energy systems in the UK. Environmental Innovation and Societal Transitions, 47, 100719.

Smith, S. M., Geden, O., Gidden, M. J., Lamb, W. F., Nemet, G. F., Minx, J. C., Buck, H., Burke, J., Cox, E., Edwards, M. R., Fuss, S., Johnstone, I., Müller-Hansen, F., Pongratz, J., Probst, B. S., Roe, S., Schenuit, F., Schulte, I., Vaughan, N. E. (eds.) The State of Carbon Dioxide Removal 2024 - 2nd Edition. DOI 10.17605/OSF.IO/F85QJ (2024)

Sovacool, B. K., Martiskainen, M., Hook, A., & Baker, L. (2019). Decarbonization and its discontents: a critical energy justice perspective on four low-carbon transitions. Climatic Change, 155(4), 581-619.

UNFCCC (2025a). Malaysia's Third Iteration of the Nationally Determined Contribution (NDC 3.0). https://unfccc.int/sites/default/files/2025-10/Malaysia%20NDC%203.0%20to%20UNFCCC%202025%20final.pdf

UNFCCC (2025b). Norway's Nationally Determined Contribution for 2035. https://unfccc.int/sites/default/files/2025-06/Norways%20NDC%20for%202035.pdf
