# Supplementary material for: Public support for novel carbon removal hinges on procedural and distributive fairness
Source: Nat Clim Chang. 2026 Sep 24;16(10):1152–61. doi: 10.1038/s41558-026-02741-7 (PMC13638447; doi:10.1038/s41558-026-02741-7)
Supplement: Supplementary file 2 [file 41558_2026_2741_MOESM2_ESM.pdf]

nature portfolio

Corresponding author(s): Fritz Livia

Last updated by author(s): 16/07/2026

Reporting Summary

Nature Portfolio wishes to improve the reproducibility of the work that we publish. This form provides structure for consistency and transparency in reporting. For further information on Nature Portfolio policies, see our Editorial Policies and the Editorial Policy Checklist.

Statistics

For all statistical analyses, confirm that the following items are present in the figure legend, table legend, main text, or Methods section.

n/a	Confirmed	
☐	☒	The exact sample size (*n*) for each experimental group/condition, given as a discrete number and unit of measurement
☐	☒	A statement on whether measurements were taken from distinct samples or whether the same sample was measured repeatedly
☐	☒	The statistical test(s) used AND whether they are one- or two-sided<br>*Only common tests should be described solely by name; describe more complex techniques in the Methods section.*
☐	☒	A description of all covariates tested
☐	☒	A description of any assumptions or corrections, such as tests of normality and adjustment for multiple comparisons
☐	☒	A full description of the statistical parameters including central tendency (e.g. means) or other basic estimates (e.g. regression coefficient) AND variation (e.g. standard deviation) or associated estimates of uncertainty (e.g. confidence intervals)
☐	☒	For null hypothesis testing, the test statistic (e.g. *F*, *t*, *r*) with confidence intervals, effect sizes, degrees of freedom and *P* value noted<br>*Give P values as exact values whenever suitable.*
☒	☐	For Bayesian analysis, information on the choice of priors and Markov chain Monte Carlo settings
☐	☒	For hierarchical and complex designs, identification of the appropriate level for tests and full reporting of outcomes
☒	☐	Estimates of effect sizes (e.g. Cohen's *d*, Pearson's *r*), indicating how they were calculated

Our web collection on statistics for biologists contains articles on many of the points above.

Software and code

Policy information about availability of computer code

Data collection	The questionnaire was coded and administered by the survey company "Norstat".
Data analysis	Analysis was performed in R and R Studio versions 4.4.2 (2024-10-31)

For manuscripts utilizing custom algorithms or software that are central to the research but not yet described in published literature, software must be made available to editors and reviewers. We strongly encourage code deposition in a community repository (e.g. GitHub). See the Nature Portfolio guidelines for submitting code & software for further information.

Data

Policy information about availability of data

All manuscripts must include a data availability statement. This statement should provide the following information, where applicable:

- Accession codes, unique identifiers, or web links for publicly available datasets
- A description of any restrictions on data availability
- For clinical datasets or third party data, please ensure that the statement adheres to our policy

The dataset employed for this study is publicly available on the Open Science Framework web platform at the following link: https://osf.io/2xgc6/overview?view_only=a8234ab90d404115b04bce9bbbf19879. The code developed for this study is publicly available on the Open Science Framework web platform at the same link.

Research involving human participants, their data, or biological material

Policy information about studies with human participants or human data. See also policy information about sex, gender (identity/presentation), and sexual orientation and race, ethnicity and racism.

Reporting on sex and gender	The dataset includes information on the respondents' gender. In each country, the final sample of participants was defined based on selected demographic quotas, one of these being gender. This information was self-ascribed by the respondents who, to the question "what is your gender?", had the possibility of choosing among: female, male, non-binary, other, prefer not to say. Gender is used as covariate in some of the analysis included in the study as dummy variable (1= Male, 0=Female, Other, Prefer not to say were coded as missing together with Non-binary for it included too few respondents).
Reporting on race, ethnicity, or other socially relevant groupings	No information was collected regarding participants race, ethnicity or other socially relevant groupings. Nevertheless, the dataset includes information on respondents' household income level. Income was self-ascribed by the respondents who, to the question "what is your household monthly gross income (before-tax)" could select a country-specific category. This question included the opt-out options "don't know" and "prefer not to say". Information regarding respondents' self-reported educational level is included in the dataset and described in the next paragraph.
Population characteristics	Beyond gender, the final sample of participants was defined based on country specific quotas on age (between 18 and 75) and geographic region, with broad quotas for education (i.e. above and below median levels for each country). Geographic region of residence is an operating variable from the survey company which administered the survey and for anonymization reasons is not included in the final dataset. Age was asked as a numeric entry to the question "what is your age". Education was self-ascribed by the respondents who, to the question "what is the highest level of education you have completed" could select a country-specific category. This question did not include an opt-out option. These demographic characteristics, except geographic region, were included as socio-demographic covariates in the data analysis in the following manner: age, as a continuous variable in age brackets; education as dummy variable. The final sample includes 10852 respondents, around 1800 in each country.<br>The topic of this study relates to climate change, which was stated in the welcome page of the survey. This is likely to have determined self-selection among the respondents, i.e. individuals who feel most critical towards this topic may have not participated in our study. Moreover, in most countries, except Italy, the majority of the respondents reported a level of education that we consider as "high". In addition, although online panels allow rapid data collection and targeted quota matching, they introduce certain limitations: respondents must have an internet connection and a suitable digital device, thus, they are typically more engaged online and more accustomed to survey-taking. These characteristics might imply that they differ from the general population in unobserved ways. To mitigate these drawbacks, the researchers and the survey provider agreed on strict quality-control procedures such as three attention checks, response-time monitoring, and removal of low-quality responses. These measures were aimed at enhancing data reliability, yet it remains possible that the panel-based recruitment influenced data quality by favoring individuals more familiar with survey environments, which limits the generalizability of our claims.
Recruitment	The survey was implemented by Norstat, a professional data collection company. The company recruited participants based on predefined quotas, took care of coding, translating, distributing and administering the survey. Norstat scripted the survey according to the indication provided by the research team together with the master questionnaire in English language and administered translations into the respective national languages (Italian, Norwegian, Portuguese, Arabic, Malay). Survey programming and translations were checked internally by members of the team. Data collection took place between June 23rd and July 21st, 2025. The final dataset was received by the researchers on July 22nd, 2025. Informed consent was requested from all participants before beginning the study. This included a description of the purposes of the survey, how their data would be handled - anonymous; in accordance with the EU General Data Protection Regulation - and about their right to withdraw their participation at any time. Upon successful survey completion, participants received their monetary compensation directly from the professional survey firm, Norstat.
Ethics oversight	The study has been approved by the Aarhus University's Research Ethics Committee for the faculty of Business and Social Sciences (Institutional Review Board) in accordance with the guidelines of the university's Research Ethics Committee (approval number: BSS-2024-121-S2). All survey respondents provided their full and informed consent.

Note that full information on the approval of the study protocol must also be provided in the manuscript.

Field-specific reporting

Please select the one below that is the best fit for your research. If you are not sure, read the appropriate sections before making your selection.

☐ Life sciences ☒ Behavioural & social sciences ☐ Ecological, evolutionary & environmental sciences

For a reference copy of the document with all sections, see nature.com/documents/nr-reporting-summary-flat.pdf

Behavioural & social sciences study design

All studies must disclose on these points even when the disclosure is negative.

Study description	Quantitative study aimed at gathering public perceptions of selected Carbon Dioxide Removal (CDR) methods (Direct Air Capture with Carbon Storage - DACCS, Bioenergy Carbon Capture and Storage - BECCS, Enhanced Rock Weathering - ERW) with representative samples (gender, age, geographical area of residence, education) in six countries (UK, Italy, Norway, Brazil, Malaysia,

Saudi Arabia).

Research sample	The total sample consisted of 10852 participants with at least 1800 in each of the 6 countries surveyed. Surveys were nationally representative of country populations regarding age (between 18 and 75) gender and geographic region, with broad quota targets set for education (i.e. above and below median levels for each country). The final distributions of "gender" are as follows: UK 51% Female, 48% Male, 1% Non-binary; IT 51% Female, 49% Male; NO 47% Female, 52% Male; BR 50% Female, 49% Male; SA 48% Female, 52% Male; MY 53% Female, 46% Male. The final distributions of age are as follow: UK 40% 18-44, 60% 45-75; IT 41% 18-44, 59% 45-75; NO 43% 18-44, 57% 45-75; BR 65% 18-44, 35% 45-75; SA 72% 18-44, 28% 45-75; MY 70% 18-44, 30% 45-75. The final distributions of "education" are as follows: UK 36% Lower 63% Higher 1% No answer; IT 52% Lower 48% Higher; NO 37% Lower 62% Higher; 1% No answer; BR 35% Lower 65% Higher SA 40% Lower 60% Higher MY 33% Lower 67% Higher; 1% No answer.<br><br>The size of the sample (and the technology and country sub-samples) is aligned with previous literature and was decided to guarantee sufficient respondents to all experimental conditions - i.e. every experimental scenario was assessed around 250 times in each country. Budget and time considerations contributed as a limitation for the overall sample size. In some countries it will be more difficult to sample those above 65, which justifies the need for looser quotas here. For similar reasons, the quotas on education are not imposed on specific levels but rather broadly in relation to above and below the median in a given country. Nevertheless, the authors are confident that for the size of the experiment the size of final samples is sufficiently powered for the analysis envisioned.
Sampling strategy	Data collection was administered online by Norstat using quota sampling to ensure national representativeness of country populations (age, gender, geographical area of residence, education) in six countries ((UK, Italy, Norway, Brazil, Malaysia, Saudi Arabia).<br>Countries and CDR technologies were selected according to a variety of criteria. Technological diversity: each country pair revolves around potential for at least one novel CDR method: for DACCS Norway and KSA; for BECCS UK and Brazil; for ERW Italy and Malaysia. Geographic diversity: a mix of European and non-European countries with a total of no more than six countries. Feasibility and potential: each country has carbon removal potential or is currently a frontrunner. Governance: countries represent a diversity of political systems, including different types of democracies and authoritarian or illiberal regimes and varieties of economic systems. Political economy: a mix of fossil fuel regimes and producers and lower-carbon countries. Novelty: countries that are not already overstudied in the literature (e.g. United States or Germany). Data efficacy: countries where representative data can be collected within our budget and reliable by a professional survey firm.
Data collection	Data collection was administed online by a professional survey firm, Norstat, who scripted and administered the survey, provided the sample of participants, and remunerated those who took part. The final survey was administered as Computer Assisted Web Interviews (CAWI) through a software of property of the survey firm in which participants completed the whole survey independently without the assistance of an interviewer (i.e. we assume only the respondents were present during data collection, and the experiment).
Timing	Data collection took place between June 23rd and July 21st, 2025. The final dataset was received on July 22nd, 2025.
Data exclusions	The questionnaire included two open-ended questions. Data from these was not employed in this study, but the answers to these questions were employed to spot duplicates in the dataset. By inspecting for duplicated answers on these questions in each countries, we identified N=7 duplicates. We excluded these respondents from the final analysis. Moreover, respondents who failed the attention check, sent incomplete responses, sped through the questionnaire (<30% of median), withdrew or refused consent have been excluded from the final sample.
Non-participation	As reported from the survey company Norstat. The total sample of online panelists that started the survey is 19439. Of these, 19% (3592) were terminated for failing at least one attention check (termination rate) and 21% (4097) dropped out from the survey (dropout rate). Thus, 60% (11750) completed the survey (raw completion rate), but 8% (891) of them were screened out at quality checks (screened out rate), so 56% of the original sample were included in the final sample of 10859 respondents (qualified completion rate).
Randomization	Respondents were assigned randomly to 5 of the full sample of 36 vignettes without replacement, although with some back-end constraints making sure each attribute dimension was shown at least once, except for removal capacity on which there was no constraint. Such programming of the allocation of vignettes was aimed at rendering the distinction between the vignettes easier for respondents while guaranteeing random allocation of respondents to vignettes.

Reporting for specific materials, systems and methods

We require information from authors about some types of materials, experimental systems and methods used in many studies. Here, indicate whether each material, system or method listed is relevant to your study. If you are not sure if a list item applies to your research, read the appropriate section before selecting a response.

Materials & experimental systems

n/a	Involved in the study
☒	☐ Antibodies
☒	☐ Eukaryotic cell lines
☒	☐ Palaeontology and archaeology
☒	☐ Animals and other organisms
☒	☐ Clinical data
☒	☐ Dual use research of concern
☒	☐ Plants

Methods

n/a	Involved in the study
☒	☐ ChIP-seq
☒	☐ Flow cytometry
☒	☐ MRI-based neuroimaging

Plants

Seed stocks	*Report on the source of all seed stocks or other plant material used. If applicable, state the seed stock centre and catalogue number. If plant specimens were collected from the field, describe the collection location, date and sampling procedures.*
Novel plant genotypes	*Describe the methods by which all novel plant genotypes were produced. This includes those generated by transgenic approaches, gene editing, chemical/radiation-based mutagenesis and hybridization. For transgenic lines, describe the transformation method, the number of independent lines analyzed and the generation upon which experiments were performed. For gene-edited lines, describe the editor used, the endogenous sequence targeted for editing, the targeting guide RNA sequence (if applicable) and how the editor was applied.*
Authentication	*Describe any authentication procedures for each seed stock used or novel genotype generated. Describe any experiments used to assess the effect of a mutation and, where applicable, how potential secondary effects (e.g. second site T-DNA insertions, mosiacism, off-target gene editing) were examined.*
