# Supplementary material for: Public support for novel carbon removal hinges on procedural and distributive fairness
Source: Nat Clim Chang. 2026 Sep 24;16(10):1152–61. doi: 10.1038/s41558-026-02741-7 (PMC13638447; doi:10.1038/s41558-026-02741-7)
Supplement: Supplementary file 3 [file 41558_2026_2741_MOESM3_ESM.pdf]

nature portfolio

Peer Review File

Public support for novel carbon removal hinges on procedural and distributive fairness

Corresponding Author: Professor Livia Fritz

This file contains all reviewer reports in order by version, followed by all author rebuttals in order by version.

Version 0:

Reviewer comments:

Reviewer #1

(Comments for the Author)
The manuscript “Public support for novel carbon removal hinges on procedural and distributive fairness” presents the analysis and results of nationally representative surveys conducted in six countries across the Global South and Global North, arguing that procedural and distributive fairness are key for CDR scale-up. Given the current limited understanding of the enablers and barriers to large-scale CDR deployment, the study has the potential to make a valuable contribution to the literature – especially given its comparative approach. However, I identify four broader issues that should be addressed before a publication.

First, the manuscript is based on the assumption that large-scale deployment of CDR is contingent upon public support. One can argue that historical experience with the scaling of other technologies suggests that public support is not necessarily a prerequisite for deployment. Reflecting on whether and if so how it is is particularly relevant in a study that compares different political systems, governance structures, and policy regimes and regulatory styles. At a minimum, this premise should be made more explicit and critically reflected based on existing literature upon in the manuscript; otherwise, the analysis risks appearing to exhibit a form of confirmation bias (especially if the social desirability bias of respondents is taken into account). Moreover, given the currently limited empirical evidence on public support for actual CDR projects in local communities, and considering that the renewable energy examples cited (lines 30–54) differ substantially from CDR in socio-technical and political terms, the manuscript should more clearly articulate and strengthen its reasoning regarding the relationship between public support and CDR deployment.

Second, the questionnaire does not address the different functions that CDR can fulfil within mitigation strategies. While it is understandable that survey respondents cannot be expected to engage with highly complex technical distinctions, it is unfortunate that the different use cases for CDR—namely offsetting fossil emissions, counterbalancing residual emissions, and reversing temperature overshoot—are not differentiated (at least based on my reading of the materials). These distinctions are particularly important because CDR is fundamentally different from most other mitigation options in terms of its multiple policy and strategic roles. If the survey design did not allow for this differentiation, the manuscript should explicitly acknowledge that the specific use case of CDR was not addressed. This omission is potentially consequential, as existing public acceptance literature suggests that perceptions and support may vary significantly depending on the perceived purpose of CDR deployment.

Third, the manuscript does not provide sufficient contextualization of the national policy environments in which the surveys were conducted. Existing literature on CDR governance demonstrates that countries are adopting highly differentiated approaches to integrating CDR into climate policy. Embedding the analysis within a short synthesis of key developments in national CDR policy frameworks would therefore significantly strengthen the interpretation of the results. An important dimension in this regard is politicisation (its absence briefly noted in line 279). The example of Brazil, for instance, illustrates how biomass—central to BECCS—has become politically polarised. Such nationally specific dynamics may meaningfully shape public perceptions and responses in the future. At a minimum, the current state of national policy debates and processes should be addressed in supplementary material and briefly reflected upon in the main manuscript.

Finally, and linked to the previous point, the relatively moderate differences observed across countries and socio-demographic groups are in themselves an interesting finding, particularly given the substantial divergence in national CDR policy strategies. At present, the manuscript does not sufficiently enable interpretation of the results within the wider literature

on similar studies on public support for technological deployment and large-scale socio-technical transitions. The manuscript would benefit from situating these results in a broader comparative context: should larger differences have been expected, and how do these findings compare with similar cross-national studies on other emerging technologies or infrastructure transitions?

(Remarks on code availability)

(Remarks on code availability)

Reviewer #2

(Comments for the Author)
I have read the manuscript "Public support for novel carbon removal hinges on procedural and distribute fairness," which examines public support for carbon removal technologies. The study uses survey experiments conducted on representative samples in six countries. The main finding is that information treatments signaling procedural fairness and distributive fairness are more important for public support than information about the effectiveness or capacity of these technologies.
Overall, I consider this to be a study with many strengths. To my knowledge, it is one of the first large-scale studies of public acceptance of carbon dioxide removal technologies. It is conducted across a relatively large number of countries. The paper is also clear about the difficulties of comparing means or proportions across countries, given potential translation effects and similar issues, and instead focuses primarily on the survey experiments. These experiments are, overall, well designed, and the data and results are presented in a pedagogical and robust manner.
Publishing in Nature Climate Change should be demanding. However, I cannot identify any obvious flaws or errors in the manuscript. I therefore recommend that the editors invite the authors to submit a revised version. Below are issues that I believe the authors should address:
1. Stronger motivation for country selection is needed. At present, the study includes six countries from "the Global South and the Global North"without a clear justification for why these particular cases were chosen. Some of the countries are also quite distinctive (Saudi Arabia, for example), and it is not obvious whether it is even reasonable to ask citizens about their opinions in such a political context. What, exactly, is the comparative ambition of this analysis? Is the aim to test whether the effects are robust across different contexts?
2. A related question concerns how reasonable it is to ask citizens in these countries about these technologies in the first place. Do respondents even know what these technologies are? Are these salient issues, technologies that people are familiar with? The authors themselves suggest that familiarity is probably limited. How much explanation is therefore required? And perhaps most importantly, given the previous question, to what extent does this vary across contexts? Could these be issues that are on the political agenda in Norway but not at all in Brazil? In Brazil, for instance, much carbon dioxide uptake occurs through large forest areas, whereas this type of "natural" carbon removal is very limited in Saudi Arabia. The reader needs more contextual information on how these issues vary across countries. Are there concrete plans for such technologies in, for example, Norway but not in Malaysia?
3. Along similar lines, the information vignettes should be discussed in greater detail across different contexts. Are local referendums of this kind even plausible or meaningful in a Saudi Arabian context?
4. Looking at Figure 1, the distributions in Brazil and Saudi Arabia appear to be highly skewed. This deserves further discussion. More generally, how do the authors assess data quality across the different countries?
5. I also think the authors need to explain earlier in the manuscript what the costs of these technologies are. Why should there be opposition? Is it related to financing, land-use policy, or something else?
6. Relatedly, the reader would benefit from more information about how the different carbon removal technologies differ from one another, and what kinds of specific costs might be associated with each technology.
7. Arnstein's ladder of participation is introduced somewhat abruptly. Its inclusion would benefit from clearer and stronger justification.
8. Finally, the reader would also like to know more about what types of research and bodies of knowledge this study speaks to. Is it primarily relevant for climate transition research more broadly, for research on public acceptance of new industries and technologies, or for studies of public acceptance in general? The manuscript would benefit from a clearer articulation of its broader scholarly contribution.

(Remarks on code availability)

(Remarks on code availability)

Reviewer #3

(Comments for the Author)
1. The manuscript is generally sound, with no major methodological concerns. However, a stronger conceptual justification for the choice of Brazil, Malaysia, Saudi Arabia, Italy, Norway, and the UK is required. Explicitly articulating the comparative rationale, whether based on similarities, contrasts, or strategic relevance to carbon removal and fairness considerations, would enhance the robustness and credibility of the analysis.

2. The manuscript could be improved by providing a more comprehensive explanation of the respondent selection procedure in all countries that are included in the study. Particularly, the manner in which respondents were selected within each country is unclear (e.g., whether sampling was conducted nationwide or specific regions or states, as in Malaysia). I suggest that the authors provide a rationale for the selection of respondents across the selected regions in each country and elucidate the sampling procedure."

(Remarks on code availability)

(Remarks on code availability)

Reviewer #4

(Comments for the Author)
This is a relevant and clearly written contribution to still underrepresented social science perspectives on the CDR and its scaling, demonstrating that societal support for novel CDR methods strongly depends on procedural and distributive fairness. Particularly relevant findings include that socio-technical features are more relevant in determining support than features of individual CDR technologies, and that political orientation exerts only very limited influence on support.
However, while the study is of interest and grounded in a novel and extensive set of data from countries in the Global North and South, major revisions are required to better articulate the specific contribution and the novelty of this article, as well as its limitations.
Most importantly, the study would benefit from being more precise about the research gap it seeks to address, and the related novelty of its contribution. L. 55-63 correctly argue that qualitative and deliberative research highlights context and governance concerns, but suggests that governance-related factors have not been taken into account in prior quantitative research. However, for instance, Spence et al. (2021) demonstrate that communication and framing critically affects public support for ERW, and Malakar et al. (2025) show that trust in institutions significantly influences support for CDR. The contribution of the study could be clarified by engaging more systematically with these findings of previous research and outlining how it goes beyond them, instead of framing the article’s novelty primarily in terms of the number of technologies and countries considered.
Moreover, the key finding – i.e. that support hinges on procedural and distributive fairness – is important but hardly surprising. Enhancing participation and sharing profits arguably increases public support for almost any novel technology or infrastructure project, and this has been demonstrated specifically for renewable energy deployment by various studies. Therefore, more could be done to justify why it is not enough to extrapolate factors determining social support from literatures on support for renewables or other novel technologies, i.e. what makes CDR distinct (e.g., mitigation deterrence risks, framing as geo-engineering etc.) and how this inhibits the possibility to draw direct parallels from renewable or low-carbon technologies, warranting additional large quantitative studies like this.
The study should also be more explicit about how and why different dimensions of justice were conceptualized and operationalized. The first para of the results sections mentions a justice framework in which the study is grounded, but this should be unpacked, also justifying why the presentation of results then hinges on the notion of fairness (rather than justice). As the study raises key issues about who decides and benefits, and who may potentially bear harms, the notion of justice may be more appropriate than fairness as the main conceptual angle.
Regarding methodology, country selection criteria are explained in the SI but this should be justified more clearly in the method section and briefly also at the beginning of the result section of the paper. The way participants were recruited for the study and how this may have impacted data quality is currently not explained sufficiently and should be made transparent.
Last but not least, the discussion and conclusion could be significantly strengthened by shifting the focus from summarizing the main findings to discussing their implications and limitations. Regarding limitations, the findings are strongly constrained by the fact that most CDR methods remain unfamiliar to the public and largely unpoliticised, especially compared to renewables. While the authors acknowledge unfamiliarity and low level of politicization, the implications for the findings should be discussed more systematically. What lessons from the politicization of renewables or low-carbon technologies may be relevant for CDR if it will eventually be deployed at larger scale, and why? Or is CDR unlikely to become politicized along the same or similar cleavages? (and if so, why?)
Moreover, what are the implications of your findings for overall expectations regarding the scalability of CDR technologies? Paying attention to distributive and procedural justice when deploying CDR may enhance the capacity to scale CDR (as you argue based on survey data), but participation in actual project development may also result in more opposition once risk perception becomes grounded in knowledge of implications on the ground. For instance, the BECCS vignette states – given research exploring land use implications in a rather understated way – that “[g]rowing the plants for bioenergy production requires a lot of land and water. This can compete with food production or nature conservation.” – but what if such abstract risks turn into real land conflicts affecting rural populations, e.g., in Brazil? Meanwhile, sharing profits or deploying CDR publicly may undermine capacity to scale CDR for other reasons, i.e., because deploying CDR is not perceived as sufficiently profitable by private capital or because of fiscal constraints. These limitations, implications and tensions need to be discussed more thoroughly (while shortening the recapitulation of main results in the discussion section) to substantiate the overall argument.

Minor issues:
- l. 72 and following: Denoting countries as WEIRD and non-WEIRD appears odd. If this is a reference to Joseph Henrich, it should be made explicit and the relevance for the study justified. As it stands, the abbreviation seems to run counter to the study’s aim to demonstrate how support varies within countries due to socio-demographic characteristics (i.e., people in

“WEIRD” countries not being highly educated or rich). Moreover, the implicit assumption of a coupling between the West and democracy (as if a tendency toward authoritarianism were an inherent feature of the Global South) appears inappropriate in light of recent authoritarian developments in the North.
- l. 294-295 (“Removing carbon dioxide from the atmosphere is emerging as a central pillar of global climate policy, alongside rapid emissions reductions.”): Can be misread to imply that rapid emissions reductions are already underway – consider revising.
- l. 317-319 (“Responding to these widespread preferences for public engagement raises questions about how to do so meaningfully and whether developer-led engagement processes are best suited for ensuring just and responsible deployment”): This reads as convoluted and overly cautious — consider writing in a more direct, plain-spoken manner.
- l. 322 (“exact modes and timings of engagement are not at the forefront of their concerns.”): How is this claim grounded in the data?
- l. 328: What is a “progressive approach to profit management”? Also a rather convoluted and seemingly avoiding a more direct positioning which would be more appropriate.
- l. 386 (“implementation of of CDR”): remove duplicate words

Spence, E., Cox, E. & Pidgeon, N. Exploring cross-national public support for the use of enhanced weathering as a land-based carbon dioxide removal strategy. Climatic Change 165, 23 (2021). https://doi.org/10.1007/s10584-021-03050-y
Malakar, Y. et al. Beyond environmental identity: Testing public support for novel carbon dioxide removal in Australia using structural modelling. Journal of Environmental Management 394, 127633 (2025). https://doi.org/10.1016/j.jenvman.2025.127633

(Remarks on code availability)

(Remarks on code availability)

Version 1:

Reviewer comments:

Reviewer #1

(Comments for the Author)
As noted previously, my reading of the existing research on patterns in CDR policy and governance suggests that national policy trajectories - not only CDR-specific, but the broader policy environment relevant for CDR - do matter for how CDR projects emerge and are discussed. Early debates around novel CDR approaches already demonstrated this. For instance, in Germany, local and subsequently national opposition to CCS projects significantly shaped the discussion around CDR. These cases illustrate how public perceptions of specific project configurations can be influenced not only by their local characteristics, but also by prior policy trajectories, political framing, and national-level controversies.

Against this background, I do not agree with the statement in the Supplementary Information that “we thus do not expect the national policy context to influence the perceptions of the different local project configurations in our vignette experiment.” This assumption appears too strong.At the same time, I do not think that this study needs to incorporate a systematic assessment of policy and governance developments related to novel CDR. However, it would strengthen the manuscript to engage more explicitly with the existing literature, particularly (comparative) case studies that analyse patterns in CDR policy and governance.

(Remarks on code availability)

(Remarks on code availability)

Reviewer #2

(Comments for the Author)
Second review
I appreciate the authors' efforts to revise the manuscript and to engage with earlier comments. The paper has improved in several respects. However, I still have some important concerns that I believe need to be addressed.
First, I am not convinced that the highly skewed results for Brazil and Saudi Arabia should be relegated to the supplementary material. These findings raise substantive issues that are central to the interpretation of the study. At least two plausible explanations should be considered more explicitly in the main text: (1) that the skewness reflects data quality concerns, such as inattentive respondents or respondents providing socially desirable answers rather than their genuine views; or (2) that the results reflect meaningful contextual differences between these countries and the others in the sample. In either case, these patterns are sufficiently striking that most readers will notice them, and they therefore warrant fuller discussion in the main manuscript rather than in an appendix.
Second, while I appreciate that the authors now engage more with differences in regime type, I find the terminology somewhat imprecise. Referring to Saudi Arabia as a "more centralized governance" system risks obscuring important distinctions. In the comparative politics literature, Saudi Arabia is more accurately described as a "closed autocracy" or "absolute monarchy".
Third, I remain unconvinced by the argument that if respondents in Saudi Arabia lacked well-formed opinions about public consultations, we would expect null or only very modest effects in the relevant experimental dimension. This inference seems too strong. The presence of variation in responses does not, in itself, demonstrate that respondents interpret the survey items in the intended way. There remains a substantial risk that key concepts, such as public consultation, are not understood equivalently across contexts. This concern is particularly salient in cross-national research that includes both democratic and authoritarian settings. I therefore encourage the authors to acknowledge this limitation more explicitly and to discuss its implications for the interpretation of the findings.

(Remarks on code availability)

(Remarks on code availability)

Reviewer #4

(Comments for the Author)
Thank you for responding to our comments and concerns in a diligent and very constructive way. Through the revisions we think the manuscript has much improved, especially with regard to clarifying its novelty and strengthening the discussion of implications and limitations. The paper now makes an interesting contribution to the literature on governance architecture and public support for the deployment of CDR. In our view it is ready for publication.

(Remarks on code availability)

(Remarks on code availability)

Version 2:

Reviewer comments:

Reviewer #2

(Comments for the Author)
The authors have responded to my comments and made changes accordingly. I think this is ready for publication.

(Remarks on code availability)

(Remarks on code availability)

Open Access This Peer Review File is licensed under a Creative Commons Attribution 4.0 International License, which permits use, sharing, adaptation, distribution and reproduction in any medium or format, as long as you give appropriate credit to the original author(s) and the source, provide a link to the Creative Commons license, and indicate if changes were made.
In cases where reviewers are anonymous, credit should be given to 'Anonymous Referee' and the source.
The images or other third party material in this Peer Review File are included in the article's Creative Commons license, unless indicated otherwise in a credit line to the material. If material is not included in the article's Creative Commons license and your intended use is not permitted by statutory regulation or exceeds the permitted use, you will need to obtain permission directly from the copyright holder.
To view a copy of this license, visit https://creativecommons.org/licenses/by/4.0/

Reviewer 1

The manuscript "Public support for novel carbon removal hinges on procedural and distributive fairness" presents the analysis and results of nationally representative surveys conducted in six countries across the Global South and Global North, arguing that procedural and distributive fairness are key for CDR scale-up. Given the current limited understanding of the enablers and barriers to large-scale CDR deployment, the study has the potential to make a valuable contribution to the literature – especially given its comparative approach. However, I identify four broader issues that should be addressed before a publication.	We appreciate such a constructive set of comments, which have undoubtedly helped us improve the manuscript further.
First, the manuscript is based on the assumption that large-scale deployment of CDR is contingent upon public support. One can argue that historical experience with the scaling of other technologies suggests that public support is not necessarily a prerequisite for deployment. Reflecting on whether and if so how it is is particularly relevant in a study that compares different political systems, governance structures, and policy regimes and regulatory styles. At a minimum, this premise should be made more explicit and critically reflected based on existing literature upon in the manuscript; otherwise, the analysis risks appearing to exhibit a form of confirmation bias (especially if the social desirability bias of respondents is taken into account). Moreover, given the currently limited empirical evidence on public support for actual CDR projects in local communities, and considering that the renewable energy examples cited (lines 30–54) differ substantially from CDR in socio-technical and political terms, the manuscript should more clearly articulate and strengthen its reasoning regarding the relationship between public support and CDR deployment.	Very good point. We have now added new text to the manuscript to explicitly address it, which notes that:<br><br>"Although public support is not always a prerequisite for deployment (e.g., the U.S. CO_2 pipeline network built from the 1970s onward to serve enhanced oil recovery[16] or subsea telecommunications cables[17]), public opposition can significantly slow or even halt infrastructure deployment[18–20], and (perceived) injustices can create public backlash to climate policies[21–23]. For example, the successful deployment of low-carbon electricity technologies depends on securing acceptance across three interconnected domains: socio-political acceptance among regulators and policymakers, market acceptance among financiers and firms, and community acceptance among local host populations[24]. Failure to secure such acceptance can trigger organized opposition, manifesting in rallies, protests, or litigation, and ultimately leading to project delays, scope revisions, cancellation, or compensation claims[25]. Beyond lessons drawn from other technologies, early signals of opposition to CDR projects, such as marine carbon removal experiments in the UK and Canada[26,27] or the Coda Terminal for

	geological storage in Iceland[28], underscore the importance of engaging with public concerns and expectations "upstream"[29].
Second, the questionnaire does not address the different functions that CDR can fulfil within mitigation strategies. While it is understandable that survey respondents cannot be expected to engage with highly complex technical distinctions, it is unfortunate that the different use cases for CDR—namely offsetting fossil emissions, counterbalancing residual emissions, and reversing temperature overshoot—are not differentiated (at least based on my reading of the materials). These distinctions are particularly important because CDR is fundamentally different from most other mitigation options in terms of its multiple policy and strategic roles. If the survey design did not allow for this differentiation, the manuscript should explicitly acknowledge that the specific use case of CDR was not addressed. This omission is potentially consequential, as existing public acceptance literature suggests that perceptions and support may vary significantly depending on the perceived purpose of CDR deployment.	We agree that the different "use cases" or functions of CDR in the wider portfolio of climate action are important additional dimensions. As you rightly point out, our experimental design did not account for different use cases as adding such dimensions would have gone beyond the scope of our study's particular interest in fairness of (local) implementation set-ups. From a methodological point of view it would have, furthermore, led to an overly complex design.<br><br>We now acknowledge this in the methods section and invite future research in this direction - while we are aware of some qualitative research touching on this dimension and surveys around the effects of framing or the source of emissions, to the best of our knowledge to date there is hardly any convincing survey-based evidence on how public perceptions of CDR technologies are shaped by different use cases. If the Reviewer is aware of such studies, we would definitely appreciate their being shared with us. In any case, relating to the current state of the literature (and the broad unfamiliarity with CDR among the general public, which the Reviewer acknowledges), our paper makes multiple novel contributions, notably, its use of an experimental approach to demonstrate the importance of distinct fairness considerations for CDR projects, in a cross-country and multi-technology setting. We contend these contributions enable the current research to solidly stand on its own while indeed building towards the kind of research focus which the Reviewer helpfully recommends.
Third, the manuscript does not provide sufficient contextualization of the national policy environments in which the surveys were conducted. Existing literature on CDR governance demonstrates that countries are adopting highly differentiated approaches to integrating CDR into climate policy.	Thank you for sharing these reflections which we have taken up in the revised manuscript. Allow us to disentangle the different aspects nested in this third comment.

Embedding the analysis within a short synthesis of key developments in national CDR policy frameworks would therefore significantly strengthen the interpretation of the results. An important dimension in this regard is politicisation (its absence briefly noted in line 279). The example of Brazil, for instance, illustrates how biomass—central to BECCS—has become politically polarised. Such nationally specific dynamics may meaningfully shape public perceptions and responses in the future. At a minimum, the current state of national policy debates and processes should be addressed in supplementary material and briefly reflected upon in the main manuscript.	1) We would like to emphasise that our experiment focuses on the implementation of local CDR projects. Different potential national/regional policy contexts and mechanisms such as reverse auctioning, inclusion into an ETS or other still allow for local configurations as included in our experiment.<br><br>2)We have added a light description of the current CDR policy context in the six countries to the methods section and now provide more background on CDR developments in the six countries in the SI. Extensively discussing national CDR policy context in greater detail goes in our view beyond the scope of this paper, and does not strengthen its contribution. We think so particularly (i) because low levels of public familiarity suggest that CDR has not yet significantly permeated public debates beyond expert circles, (ii) and because real-world implementation is very limited to date in all of the six countries, as are CDR specific targets, policy instruments and mechanisms.<br><br>3) We agree that potential national controversies around specific methods as well as for example media coverage may in the future affect public perception. Currently, studies of (English-language) media coverage show only limited engagement with CDR and do not point to strong polarization (State of CDR report). Given the low stated familiarity in all countries included here, we are confident that such dynamics do not yet unfold in the context of our study.
Finally, and linked to the previous point, the relatively moderate differences observed across countries and socio-demographic groups are in themselves an interesting finding, particularly given the substantial divergence in national CDR policy strategies. At present, the manuscript does not sufficiently enable interpretation of the results within the wider literature on similar studies on public support for technological deployment and large-scale socio-technical	As pointed out above, the national CDR policies are differently advanced but none of the countries has a functioning CDR socio-technical ecosystem, yet. Our addition in the methods section and SI now foreground this point.<br>Generally, regarding expected differences, other multi-country studies on attitudes towards climate action showed that differences between countries tend to be smaller than within-country differences

transitions. The manuscript would benefit from situating these results in a broader comparative context: should larger differences have been expected, and how do these findings compare with similar cross-national studies on other emerging technologies or infrastructure transitions?	(Andre et al. 2024). In the discussion section we now situate our results in the context of deployment dynamics from other technologies, notably in the field of energy.<br><br>Furthermore, regarding expected differences, we note that very little survey research has so far been undertaken into the relevance of socio-technical systems of CDR technologies. To the best of our knowledge, the most notable examples are Cox et al. (2024) and Scott-Buechler et al. (2024), both of which we reference in the text. While both make several notable contributions, we note that their respective designs focused only on one country, the United Kingdom and United States, respectively, while looking at either one or two technologies; these studies also either did not explicitly look at justice or fairness considerations (Cox et al. 2024) or made limited use of such data in the paper (Scott-Buechler et al. 2024). There is, thus, to date a limited empirical basis for comparison with previous studies on CDR specifically.<br><br>Our design deliberately focuses on marginal effects of socio-political factors instead of the explanation of absolute differences. This way, we can abstract from very specific country conditions that would require more in-depth case studies beyond the scope of this study. This also renders our results more robust to absolute changes in perceptions that could happen as the national contexts develop. But at the same time, we qualify our results from different political regimes by showing differences in the conceptions of public participation in Fig. 4.<br><br>References<br>Andre, P., Boneva, T., Chopra, F., & Falk, A. (2024). Globally representative evidence on the actual and perceived support for climate action. *Nature Climate Change, 14*(3), 253-259.

Reviewer 2

I have read the manuscript "Public support for novel carbon removal hinges on procedural and distribute fairness," which examines public support for carbon removal technologies. The study uses survey experiments conducted on representative samples in six countries. The main finding is that information treatments signaling procedural fairness and distributive fairness are more important for public support than information about the effectiveness or capacity of these technologies.<br>Overall, I consider this to be a study with many strengths. To my knowledge, it is one of the first large-scale studies of public acceptance of carbon dioxide removal technologies. It is conducted across a relatively large number of countries. The paper is also clear about the difficulties of comparing means or proportions across countries, given potential translation effects and similar issues, and instead focuses primarily on the survey experiments. These experiments are, overall, well designed, and the data and results are presented in a pedagogical and robust manner.<br>Publishing in Nature Climate Change should be demanding. However, I cannot identify any obvious flaws or errors in the manuscript. I therefore recommend that the editors invite the authors to submit a revised version. Below are issues that I believe the authors should address:	We greatly appreciate this reviewer's recognition of the study's strengths, as well as their noteworthy suggestions for improvement. We have thoroughly revised the manuscript accordingly.
1. Stronger motivation for country selection is needed. At present, the study includes six countries from "the Global South and the Global North" without a clear justification for why these particular cases were chosen. Some of the countries are also quite distinctive (Saudi Arabia, for example), and it is not obvious whether it is even reasonable to ask citizens about their opinions in such a political context. What, exactly, is the comparative ambition of this analysis? Is the aim to test whether the effects are robust across different contexts?	Thank you for raising this important point which we have implemented in the revised version. In the introduction and the methods section, we now describe the rationale guiding our country selection, as follows (version in intro, but see methods too):<br>"To address these gaps, we examine public support for different implementation pathways of novel CDR methods across six countries that vary markedly in political systems, political economies, and geographic contexts: Brazil, Malaysia, Saudi Arabia, Italy, Norway, and the United Kingdom. These countries span liberal democracies and more centralized

	governance systems, include both major fossil fuel producers and diversified economies, and represent Global North and Global South contexts where CDR governance remains nascent. By encompassing this institutional and socio-political diversity, we are able to assess whether fairness-related determinants of support are context-specific or robust across heterogeneous settings. This comparative design strengthens the external validity of our findings and allows us to identify patterns that may generalize beyond single-country case studies."<br><br>We now also refer the reader to the Supplementary Information for more details regarding. the selection criteria.<br><br>As we elaborate in reply to your observation below (comment 3), our ambition with the vignettes was to elicit what matters to people, what they envision to be desirable scenarios, rather than about assessing the current status. If respondents in Saudi Arabia did not have a formed opinion about public consultations (described as: "with / without asking the opinion of"), we would expect the corresponding dimensions of the experiment to either not show any significant result or very modest ones, which is not the case. As our now more clearly explained selection criteria show, we aim at providing insights on the role of fairness perceptions for CDR implementation in countries which have not been overstudied (e.g. USA) and which have distinctly different features, to observe the robustness of such public perceptions across contexts. Our endeavour presents us with challenges, but we believe rewards us with as many if not more insightful results.
2. A related question concerns how reasonable it is to ask citizens in these countries about these technologies in the first place. Do respondents even know what these technologies are? Are these salient issues, technologies that people are familiar with? The authors themselves suggest that familiarity is probably limited. How much	Thanks for raising these important points. Yes, as we also highlight in our article, low familiarity is a consistent concern in the literature. At the same time, there are also decades of experience and gathering of best practices regarding how to examine and gain insight into public perceptions of novel technologies, such as genetic modification,

explanation is therefore required? And perhaps most importantly, given the previous question, to what extent does this vary across contexts? Could these be issues that are on the political agenda in Norway but not at all in Brazil? In Brazil, for instance, much carbon dioxide uptake occurs through large forest areas, whereas this type of "natural" carbon removal is very limited in Saudi Arabia. The reader needs more contextual information on how these issues vary across countries. Are there concrete plans for such technologies in, for example, Norway but not in Malaysia?	nanotechnology but climate intervention as well (e.g. Pidgeon et al. 2012; Cox et al. 2020; Feindt and Poortvliet 2020). Notably, in such contexts, it is important to provide some background information to the participants - here we paid attention to the use of simple, accessible language along with a balanced presentation of potential risks and benefits while avoiding terminology that has been shown to have strong framing effects, e.g. referring to "naturalness" (or not) of certain options. We also employed pre-testing to ensure that information texts were sufficiently clear, and provided opt-out response options so that respondents would not feel compelled to answer about subjects where they had not yet developed an opinion or evaluation.<br><br>We would also observe, as a more general point, that people still make decisions in day-to-day life, or form (provisional) evaluations, about subjects with which they are unfamiliar. That is to say, lack of familiarity with a subject is far from a cause not to engage with publics on an important topic in an early and ongoing manner.<br><br>Finally, regarding the important question about how much familiarity varies across contexts, as shown in SI8.3, we note that familiarity is broadly low across all the countries with means ranging from 1.12 (sd=0.94) and 1.57 (sd=0.74) on a scale on which 0=Familiarity, 1=Familiarity with another CDR, 2=Complete lack of familiarity. To avoid a strongly unequal basis of comparison, we did, for instance, deliberately not include afforestation or reforestation as one of the carbon removal methods, but rather chose three novel methods for which similar levels of unfamiliarity could be assumed based on previous literature. The fact that this was ultimately confirmed by our data also provides further support on this point - and reasons for less concern here.<br><br>In the discussion we now reflect on the implications of low familiarity and

	contextualize the findings looking at polarization of other climate mitigation measures.<br><br>References:<br>Pidgeon, N., Corner, A., Parkhill, K., Spence, A., Butler, C., & Poortinga, W. (2012). Exploring early public responses to geoengineering. *Philosophical Transactions of the Royal Society A: Mathematical, Physical and Engineering Sciences*, *370*(1974), 4176-4196.<br>Cox, E., Spence, E., & Pidgeon, N. (2020). Public perceptions of carbon dioxide removal in the United States and the United Kingdom. *Nature Climate Change*, *10*(8), 744-749.<br>Feindt, P. H., & Poortvliet, P. M. (2020). Consumer reactions to unfamiliar technologies: mental and social formation of perceptions and attitudes toward nano and GM products. *Journal of Risk Research*, *23*(4), 475-489.
3. Along similar lines, the information vignettes should be discussed in greater detail across different contexts. Are local referendums of this kind even plausible or meaningful in a Saudi Arabian context?	Thanks for raising this valid point. When designing the vignette experiment we paid due attention to using concepts and wording that would work across contexts.<br>For example, we did deliberately not speak of a referendum (or even consultation), but rather described opening the decision process in the following way: "This project is permitted by the authorities with/without asking the opinion of the affected local population".<br>In general, the ambition of the vignettes is to elicit what matters to people, what they envision to be desirable scenarios, rather than about assessing the current status. If respondents in Saudi Arabia did not have a formed opinion about public consultations, we would expect the corresponding dimensions of the experiment to either not show any significant result or a very modest one. Even if it is indeed true that the corresponding AMCEs of the "procedural fairness" items are the smallest in this country, they do not appear to be statistically different from those of Malaysia or even Norway. Hence, we contend that our results are not only valid, but also

	insightful in light of exactly this apparent tension.<br><br>We have added a sentence on this in the corresponding paragraph of the results, which reads:<br>"Despite currently limited opportunities for public consultations in the Saudi Arabian context, the effect of procedural fairness on support is substantial and in absolute terms not far from those observed in Malaysia or Norway".
4. Looking at Figure 1, the distributions in Brazil and Saudi Arabia appear to be highly skewed. This deserves further discussion. More generally, how do the authors assess data quality across the different countries?	Another important observation, which we have indeed carefully considered.<br>In the "Data Analysis" section of the Methods, we had noted that the skewed results of Figure 1 are to be interpreted with caution as they "may reflect unobserved factors or cultural differences in survey response styles, rather than fundamental differences in support". Indeed, as other studies observe (Smith et al., 2016; Beuthner et al., 2018), since survey instruments were developed by and for the Western public, non-Western respondents often show different response patterns, i.e. acquiescence bias. We are aware of this issue and to tackle it, we have proceeded in three ways:<br>a) In the main analysis: we interpret differences and similarities in the average marginal effects of the vignette attribute.<br>b) In the SI11: we provide a robustness check in which we perform the same analysis, but excluding respondents who selected the same value across vignettes. This is surprisingly not as much the case for Saudi Arabia and Brazil, which suggests that even though these respondents tend to show higher support levels, they do discern between vignettes.<br>c) In the questionnaire: we included open-ended answers in which respondents were invited and required to motivate their choices in the vignette exercise. Among these, the responses considered nonsensical, bot-like or poor were

	excluded from the final sample, and the corresponding respondents with them.<br><br>References<br>Smith, P. B. et al. Individual and culture-level components of survey response styles: A multi-level analysis using cultural models of selfhood. Int. J. Psychol. 51, 453–463 (2016).<br><br>Beuthner, C., Friedrich, M., Herbes, C. & Ramme, I. Examining survey response styles in cross-cultural marketing research: A comparison between Mexican and South Korean respondents. Int. J. Mark. Res. 60, 257–267 (2018).
5. I also think the authors need to explain earlier in the manuscript what the costs of these technologies are. Why should there be opposition? Is it related to financing, land-use policy, or something else?	It could be all of these factors, or more, which is why some emerging research on carbon removal describes their transition dynamics as sociotechnical, as fusing together economic, political, cultural, and technical dimensions. This means that some social concerns may be over land use impacts, but also financing and other issues. In the introduction, we now explicitly state that costs for different CDR methods vary and point the reader to the State of CDR report for an overview on this topic. Regarding potential sources for opposition, we now, furthermore, provide more information on matters of public concerns they might have regarding different aspects of CDR:<br><br>"Qualitative research shows that public concerns are often tied to technology-specific trade-offs: land use conflicts and distributive injustices for BECCS, energy costs for DACCS, and extractivism and transportation burdens for ERW[44–46]. In addition, concerns about CO_2 leakage, long-term storage integrity, and broader environmental and community safety risks cut across multiple approaches[10,33,44,45], reinforcing perceptions that deployment entails not only technical but also social and ethical uncertainties."
6. Relatedly, the reader would benefit from more information about how the different carbon removal technologies differ from	In the introduction (paragraph 2) we now mention that each CDR method comes with distinct cost profiles as well as specific sets

one another, and what kinds of specific costs might be associated with each technology.	of risks and benefits. We also point the reader to the State of CDR report for further information on the different CDR methods. Space constraints do not allow us to go into the details here.
7. Arnstein's ladder of participation is introduced somewhat abruptly. Its inclusion would benefit from clearer and stronger justification.	We agree and given space constraints we decided to focus in the main text on the description of the key results. Hence, we have removed the reference to Arnstein's ladder of participation. Instead, in the SI we now include an explanation of the item that builds on Arnstein's ladder of participation.
8. Finally, the reader would also like to know more about what types of research and bodies of knowledge this study speaks to. Is it primarily relevant for climate transition research more broadly, for research on public acceptance of new industries and technologies, or for studies of public acceptance in general? The manuscript would benefit from a clearer articulation of its broader scholarly contribution.	Great suggestion. In the Introduction, we have sharpened the research gap as well as our contribution. In the final paragraph of the introduction, we now, furthermore, clarify that the paper is in fact meant for all of these audiences. As we now write:<br><br>"Although the study is most relevant for the climate and energy transition research community, it generates insights for the broader communities researching the public acceptance of new industries and technologies and even studies of public acceptance in general."

Reviewer 3

1. The manuscript is generally sound, with no major methodological concerns. However, a stronger conceptual justification for the choice of Brazil, Malaysia, Saudi Arabia, Italy, Norway, and the UK is required. Explicitly articulating the comparative rationale, whether based on similarities, contrasts, or strategic relevance to carbon removal and fairness considerations, would enhance the robustness and credibility of the analysis.	Thank you very much for the positive feedback on our manuscript and the helpful suggestions which we have implemented in the revised version.<br><br>In the methods section, we now provide a more explicit explanation of the rationale that guided the country selection. We also now state this rationale upfront in the introduction where we write (shorter version from the introduction here for reference):<br><br>"To address these gaps, we examine public support for different implementation pathways of novel CDR methods across six countries that vary markedly in political systems, political economies, and geographic contexts: Brazil, Malaysia, Saudi Arabia, Italy, Norway, and the United

	Kingdom. These countries span liberal democracies and more centralized governance systems, include both major fossil fuel producers and diversified economies, and represent Global North and Global South contexts where CDR governance remains nascent. By encompassing this institutional and socio-political diversity, we are able to assess whether fairness-related determinants of support are context-specific or robust across heterogeneous settings. This comparative design strengthens the external validity of our findings and allows us to identify patterns that may generalize beyond single-country case studies.”<br><br>In addition, in the methods section we refer the reader to the relevant section in the Supplementary Information for more details on the selection criteria.
2. The manuscript could be improved by providing a more comprehensive explanation of the respondent selection procedure in all countries that are included in the study. Particularly, the manner in which respondents were selected within each country is unclear (e.g., whether sampling was conducted nationwide or specific regions or states, as in Malaysia). I suggest that the authors provide a rationale for the selection of respondents across the selected regions in each country and elucidate the sampling procedure."	Thank you for this observation. As we now clarify in the methods section, the sampling and recruitment was conducted by a professional data collection company and it was carried out nation-wide with strict quotas on gender and age and broad quotas on education. Furthermore, quotas for geographic regions were included in order to avoid oversampling on certain areas of each country. These regional quota thresholds were set just as the others, according to the distribution of the population in each country. The survey company reported the comparison of the final sample with the population goals, although this specific variable is not included in the final dataset delivered for privacy reasons.<br>We agree that our initial formulation provided in the section “Data collection and sampling” of the Methods, was not sufficiently clear and detailed. The new text reads:<br><br>“The company recruited participants from their national online panels, took care of coding, translating, distributing and administering the survey. Quotas for national representativeness in terms of age,

	and gender, along with broad quotas for education have been defined according to the survey provider's possibilities. Moreover, in each country, specific quotas for geographic regions were set to avoid oversampling from specific areas. As with gender and age quotas, these regional quotas reflected the population distribution in each country. Researchers were provided with documentation showing how the final sample matched these population targets, which they deemed appropriate."

Reviewer 4

This is a relevant and clearly written contribution to still underrepresented social science perspectives on the CDR and its scaling, demonstrating that societal support for novel CDR methods strongly depends on procedural and distributive fairness. Particularly relevant findings include that socio-technical features are more relevant in determining support than features of individual CDR technologies, and that political orientation exerts only very limited influence on support.<br>However, while the study is of interest and grounded in a novel and extensive set of data from countries in the Global North and South, major revisions are required to better articulate the specific contribution and the novelty of this article, as well as its limitations.	We appreciate the positive feedback and support for the study and have thoroughly implemented the revisions suggested by the reviewer.
Most importantly, the study would benefit from being more precise about the research gap it seeks to address, and the related novelty of its contribution. L. 55-63 correctly argue that qualitative and deliberative research highlights context and governance concerns, but suggests that governance-related factors have not been taken into account in prior quantitative research. However, for instance, Spence et al. (2021) demonstrate that communication and framing critically affects public support for ERW, and Malakar et al. (2025) show that trust in institutions significantly	Thank you for bringing this to our attention, it was not our intention to convey the country and technology scope as the only novelty of our study. In fact, our study is first and foremost novel in its focus on governance set-ups, situating emerging public perceptions in the wider context of socio-technical configurations of CDR deployment (as opposed to perception of the technology per se).<br><br>The main aspects of novelty of our study are: (i) relation between publics' support and governance set-up, with a focus on

influences support for CDR. The contribution of the study could be clarified by engaging more systematically with these findings of previous research and outlining how it goes beyond them, instead of framing the article's novelty primarily in terms of the number of technologies and countries considered.	fairness, (ii) interactions and trade-offs between governance dimensions and removal capacity and (iii) highlighting technology and country diversity.<br><br>We now more clearly articulate the research gaps and novelty of our approach in the introduction.<br>We appreciate the suggested references, which also allow us to reiterate the novel focus of our study on conditions of deployment. Relying on the categorisation of literature used in the State of CDR report, the suggested papers identify factors driving public attitudes towards CDR technologies, but they do not specifically inquire into the conditions of deployment (i.e. the governance set-ups or configurations). The latter is the focus of our analysis.
Moreover, the key finding – i.e. that support hinges on procedural and distributive fairness – is important but hardly surprising. Enhancing participation and sharing profits arguably increases public support for almost any novel technology or infrastructure project, and this has been demonstrated specifically for renewable energy deployment by various studies. Therefore, more could be done to justify why it is not enough to extrapolate factors determining social support from literatures on support for renewables or other novel technologies, i.e. what makes CDR distinct (e.g., mitigation deterrence risks, framing as geo-engineering etc.) and how this inhibits the possibility to draw direct parallels from renewable or low-carbon technologies, warranting additional large quantitative studies like this.	Taking up this suggestion, in the introduction we now explicitly elaborate on why the specificities of CDR (and differences to energy technology) for example regarding mitigation deterrence, diffuse benefits and geoengineering framings call for targeted studies on CDR. The addition reads as follows:<br><br>"While insights from renewable energy provide an important starting point, CDR differs in several structurally significant ways. Unlike renewables, CDR typically delivers diffuse and long-term climate benefits[56] rather than salient local gains such as energy supply or employment. Moreover, it is frequently entangled in debates about mitigation deterrence and moral hazard[57–59], raising concerns that reliance on removals may delay emissions reductions. CDR is also often framed within broader discourses of geoengineering, which can evoke perceptions of technological overreach and tampering with nature[30,34,60]. These distinctive features complicate direct extrapolation from energy acceptance studies and underscore the need for targeted empirical investigation into how governance and implementation arrangements shape public support for CDR specifically"

	On your first point implicitly referring to novelty, in our study we show not only that procedural and distributive considerations matter for publics when it comes to CDR, but also that they matter more than other attributes and that they cannot be compensated for or traded. Such an insight is enabled by our experimental approach, which has not yet been widely utilized in the literature. We also show that this observation holds for diverse countries which are relatively understudied/underrepresented in the survey literature, indeed in the wider field of climate technologies (not just for CDR).
The study should also be more explicit about how and why different dimensions of justice were conceptualized and operationalized. The first para of the results sections mentions a justice framework in which the study is grounded, but this should be unpacked, also justifying why the presentation of results then hinges on the notion of fairness (rather than justice). As the study raises key issues about who decides and benefits, and who may potentially bear harms, the notion of justice may be more appropriate than fairness as the main conceptual angle.	Taking up this comment, we have added a new section "conceptual background informing the vignette design" which, due to space constraints, is located in the Supplementary Information (section 1). Here we unpack the two dimensions of justice that we focus on and explain how we translated and operationalized them in the vignettes. We also provide justification of our focus on these two dimensions.<br>In the manuscript we use the notion of fairness since this is the term used in the survey at various times. When designing the survey and collecting feedback from test respondents and colleagues, we had decided to use this term for simplicity, to enable respondent comprehension, and for translation reasons (notably to avoid confusion with legal terms).
Regarding methodology, country selection criteria are explained in the SI but this should be justified more clearly in the method section and briefly also at the beginning of the result section of the paper. The way participants were recruited for the study and how this may have impacted data quality is currently not explained sufficiently and should be made transparent.	Valid point, thank you for bringing this to our attention. We now explain the rationale guiding the country selection at the beginning of the Methods section and, due to space constraints, refer to the SI for more details. We now also point to the diversity rationale already in the introduction (where following another of your suggestions we no longer use WEIRD terminology as well).<br><br>For what concerns the sampling and recruitment, we have modified the text in the section "Data collection and sampling" in the Methods and added the following clarification:

	“The data collection company recruited participants through their online panels. Panel members are invited to join the survey based on set quotas for each country and join voluntarily. Although online panels allow rapid data collection and targeted quota matching, they introduce certain limitations: respondents must have an internet connection and a suited digital device, thus, they are typically more digitally engaged and more accustomed to survey-taking. These characteristics might imply that they differ from the general population in unobserved ways. To mitigate these drawbacks, the researchers and the survey provider agreed on strict quality-control procedures such as attention checks, response-time monitoring, and removal of low-quality responses. These measures were aimed at enhancing data reliability, yet it remains possible that the panel-based recruitment influenced data quality by favoring individuals more familiar with survey environments.”
Last but not least, the discussion and conclusion could be significantly strengthened by shifting the focus from summarizing the main findings to discussing their implications and limitations. Regarding limitations, the findings are strongly constrained by the fact that most CDR methods remain unfamiliar to the public and largely unpoliticised, especially compared to renewables. While the authors acknowledge unfamiliarity and low level of politicization, the implications for the findings should be discussed more systematically. What lessons from the politicization of renewables or low-carbon technologies may be relevant for CDR if it will eventually be deployed at larger scale, and why? Or is CDR unlikely to become politicized along the same or similar cleavages? (and if so, why?)	We agree that this is a relevant point that helps contextualise and interpret our results. We address the link between (un)familiarity and polarization in a paragraph in the discussion section. In this new paragraph we also consider literature on politicization and controversy around renewable energy technologies and point to learnings.<br><br>We write: “These results, and particularly the currently limited political polarization, need to be interpreted in light of low levels of public familiarity and only nascent CDR sectors in the six countries. Experiences with other climate mitigation technologies caution against interpreting these patterns of moderate support and limited polarization as stable equilibria. Extrapolating from renewable energy technologies[67], controversies and polarization will likely become more marked as real-world implementation advances. Early research on renewable energy and carbon capture and storage frequently reported high or majority support in abstract settings, yet real-world deployment triggered localized opposition,

	safety concerns, and politicization as projects became more tangible and costs or distributional implications became salient[24]. Similarly, public backing for climate policy instruments such as carbon pricing has proven sensitive to framing, perceived fairness, and political mobilization over time[68]. These historical dynamics suggest that attitudes toward CDR may evolve as deployment progresses, particularly if projects become associated with visible trade-offs, incumbent interests, or partisan narratives. The relative insulation of CDR from ideological polarization observed in our data may therefore reflect its current novelty rather than entrenched consensus. Identifying potential, nationally-distinct, controversy spillovers early on[69] can help policy-makers and developers anticipate and prepare for potential contestations around CDR (e.g., conflict around biofuels[70] might spill over to debates on BECCS)."
Moreover, what are the implications of your findings for overall expectations regarding the scalability of CDR technologies? Paying attention to distributive and procedural justice when deploying CDR may enhance the capacity to scale CDR (as you argue based on survey data), but participation in actual project development may also result in more opposition once risk perception becomes grounded in knowledge of implications on the ground. For instance, the BECCS vignette states – given research exploring land use implications in a rather understated way – that "[g]rowing the plants for bioenergy production requires a lot of land and water. This can compete with food production or nature conservation." – but what if such abstract risks turn into real land conflicts affecting rural populations, e.g., in Brazil? Meanwhile, sharing profits or deploying CDR publicly may undermine capacity to scale CDR for other reasons, i.e., because deploying CDR is not perceived as sufficiently profitable by private capital or because of fiscal constraints. These limitations, implications and tensions need to be discussed more thoroughly (while shortening the recapitulation of main results	This is an excellent, apt comment, and one that we have now addressed by by expanding our reflection on tensions:<br><br>"Fourth, tensions between the need for accelerating deployment to close critical carbon removal gaps[14,66] and the safeguarding of procedural considerations warrant attention moving forward[78]. Responding to the preferences for public engagement highlighted in this study raises questions about how to do so meaningfully and effectively. Project-based and developer-led engagement[79] might not be best suited for ensuring just and responsible deployment in the given timeframes. Instead, policymakers could experiment with regional-level and systemic engagement beyond discrete projects and at planning rather than implementation stages. Such upstream practices could allow for deliberating place-based scenarios for CDR in the context of wider climate action portfolios, each with their respective trade-offs around land use, costs, or efficacy from a mitigation perspective, and for productively engaging with controversy as an opportunity for learning[80] rather than as a

in the discussion section) to substantiate the overall argument.	problem downstream. Furthermore, the preference for non-profit configurations points toward the consistently low trust in private companies[81] and the tension this creates for developing business models and raising the investments to deploy CDR at scale. Navigating the inherent tensions between urgency, low trust, and due process also calls for innovations in impact assessment practices in the context of the climate transition[82]. Taken together, these tensions underscore the need for a prudent approach to estimating the future scale and pace of CDR deployment, avoiding overreliance on promises of future removals at the expense of emissions reductions today[3]. "<br><br>We note that these tensions also play a role moving forward, with new text arguing that: "Public support depends not only on the effectiveness of removal technologies but, in large part, on whether their implementation is perceived as fair and on how well the tensions above are discerned and managed."
Minor issues:<br>- l. 72 and following: Denoting countries as WEIRD and non-WEIRD appears odd. If this is a reference to Joseph Henrich, it should be made explicit and the relevance for the study justified. As it stands, the abbreviation seems to run counter to the study's aim to demonstrate how support varies within countries due to socio-demographic characteristics (i.e., people in "WEIRD" countries not being highly educated or rich). Moreover, the implicit assumption of a coupling between the West and democracy (as if a tendency toward authoritarianism were an inherent feature of the Global South) appears inappropriate in light of recent authoritarian developments in the North.	Yes, we did indeed take up terminology by Henrich here. Upon reflection, we realized that it was not necessary for the presentation of our study and decided to remove it from the manuscript.
- l. 294-295 ("Removing carbon dioxide from the atmosphere is emerging as a central pillar of global climate policy, alongside rapid emissions reductions."): Can be misread to imply that rapid	We revised the sentence which now reads "Alongside the **need** for rapid emissions reductions ..:".

emissions reductions are already underway – consider revising.	
- l. 317-319 ("Responding to these widespread preferences for public engagement raises questions about how to do so meaningfully and whether developer-led engagement processes are best suited for ensuring just and responsible deployment"): This reads as convoluted and overly cautious — consider writing in a more direct, plain-spoken manner.	We have revised this sentence and further elaborate on what an alternative could look like.
- l. 322 ("exact modes and timings of engagement are not at the forefront of their concerns."): How is this claim grounded in the data?	This statement is based on the results presented in figure 4 as well as in SI 7.1, 7.2. where we show that no strong differences or clear patterns regarding the stage or timing of participation can be observed. Across all countries, respondents do not strongly differentiate whether engagement happens in relation to permitting, siting, or the distribution of profits, although we do observe a slightly lower desire for direct involvement in decisions on how to distribute profits.<br><br>Regarding modes (or degrees) of engagement we see strong preferences for some vs. no engagement, but comparably small differences between the different degrees (particularly whether population is informed, or asked to comment).
- l. 328: What is a "progressive approach to profit management"? Also a rather convoluted and seemingly avoiding a more direct positioning which would be more appropriate.	For a lack of a better term, we spoke of a "progressive approach to profit management" to include the variation of both profit distribution and not-for-profit approaches. We will gladly consider suggestions for alternatives to this umbrella term.<br>In the meantime, we have reformulated this sentence in the discussion and now say explicitly: "Second, pursuing distributive fairness and not-for profit arrangements also consistently lead to higher support for local carbon removal projects"
- l. 386 ("implementation of of CDR"): remove duplicate words	Thanks, removed.
Spence, E., Cox, E. & Pidgeon, N. Exploring cross-national public support for the use of enhanced weathering as a land-based carbon dioxide removal strategy. Climatic Change 165, 23	We appreciate suggesting these two studies, which we now engage with and cite in the manuscript (see introduction, paragraph 3).

(2021). https://doi.org/10.1007/s10584-021-03050-y<br>Malakar, Y. et al. Beyond environmental identity: Testing public support for novel carbon dioxide removal in Australia using structural modelling. Journal of Environmental Management 394, 127633 (2025). https://doi.org/10.1016/j.jenvman.2025.127633	

Reviewer 1

As noted previously, my reading of the existing research on patterns in CDR policy and governance suggests that national policy trajectories - not only CDR-specific, but the broader policy environment relevant for CDR - do matter for how CDR projects emerge and are discussed. Early debates around novel CDR approaches already demonstrated this. For instance, in Germany, local and subsequently national opposition to CCS projects significantly shaped the discussion around CDR. These cases illustrate how public perceptions of specific project configurations can be influenced not only by their local characteristics, but also by prior policy trajectories, political framing, and national-level controversies.<br><br>Against this background, I do not agree with the statement in the Supplementary Information that "we thus do not expect the national policy context to influence the perceptions of the different local project configurations in our vignette experiment." This assumption appears too strong.<br><br>At the same time, I do not think that this study needs to incorporate a systematic assessment of policy and governance developments related to novel CDR. However, it would strengthen the manuscript to engage more explicitly with the existing literature, particularly (comparative) case studies that analyse patterns in CDR policy and governance.	Thank you for taking the time to engage with our revised manuscript and for clarifying this point.<br><br>We fully agree on the importance of contextualizing CDR developments and considering national "technopolitical identities" in which perceptions of new technologies like CDR emerge. In fact, we take up this point in the discussion section where we dedicate an entire paragraph to "controversy spillovers" (see Cuppen et al. for the conception of "technology spillovers" and "historical spillovers"). We have now expanded these reflections in the discussion section and refer explicitly to studies identifying such potential ripple effects from CCS, shale gas fracking and nuclear waste to CDR. In addition to reflecting on controversy spillovers from other technologies, we also suggest that "historical spillovers" deserve attention here. Future empirical and case study research is needed to understand how prior trajectories and national-level controversies shape public perceptions of CDR moving forward. References added on controversies:<br>Whitmarsh, L., Xenias, D. & Jones, C. R. Framing effects on public support for carbon capture and storage. Palgrave Commun. **5**, 1–10 (2019).<br>Cox, E., Pidgeon, N. & Spence, E. But They Told Us It Was Safe! Carbon Dioxide Removal, Fracking, and Ripple Effects in Risk Perceptions. Risk Anal. **42**, 1472–1487 (2022).<br>Satterfield, T., Nawaz, S., Todd, D., Jewell, K. & Romer, P. Thinking at the megaton scale: Community reflections across three different marine carbon-dioxide removal strategies. Energy Res. Soc. Sci. **134**, 104628 (2026).<br>Scott-Buechler, C. & Wang, K. H. Navigating uncertainty: direct air capture and just transition perspectives in Gulf Coast communities. Environ. Res. Lett. **20**, 094042 (2025).<br><br>On the second point, we have now removed

	the statement from the manuscript and Supplementary Information.<br><br>On the third point, we have taken up your suggestion and now engage with comparative CDR policy work in the introduction, where we synthesise and cite Schenuit et al. 2021 and Schenuit et al. 2024 (in addition to Smith et al. 2024 which we already refer to in the methods and SI descriptions of the case study contexts).<br><br>**In the Introduction:**<br>“Comparative policy work shows currently low levels of governance and regulation for (novel) CDR in both OECD countries[14] and emerging economies[15]. Nonetheless, distinct types of nascent CDR policymaking have been identified, including incremental modification of existing national policy mixes, early integration and fungibility between emissions reductions and removals, proactive CDR policy entrepreneurship[14], as well as repurposing policies, particularly those related to Land Use, Land-Use Change, and Forestry[15].While different methods entail distinct sets of risks, benefits and cost profiles[16], future CDR deployment overall will both shape and be shaped by nationally-varying socio-political, economic, and legal structures[17].”<br><br>We also come back to the importance of the broader environment and the repurposing of existing policy instruments in the discussion section (alongside additional reflections on your first point).<br><br>**In the discussion:**<br>“Because CDR governance will not emerge from a blank slate - and may instead adapt or repurpose existing policy instruments[15] - understanding how historical developments and current debates in adjacent policy fields influence perceptions of CDR is crucial. For

	example, conflict around biofuels[71] might spill over to debates on BECCS, and national policy contexts and controversies on CCS[72] or other technologies like shale gas fracking[73] and nuclear waste[47]might shape perceptions of CDR methods involving geological storage. In addition to such "technology spillovers", "historical spillovers" from past injustices and negative experiences with extractivist and pollutive industries like mining, oil and gas[28,74,75] require attention. Identifying potential nationally-distinct controversy spillovers[76] early on can help policymakers and developers anticipate and prepare for potential contestations around CDR."<br><br>In addition to new references regarding historical spillovers, we have added the following citations to comparative analysis of CDR policy:<br><br>Schenuit F, Colvin R, Fridahl M, McMullin B, Reisinger A, Sanchez DL, Smith SM, Torvanger A, Wreford A and Geden O (2021) Carbon Dioxide Removal Policy in the Making: Assessing Developments in 9 OECD Cases. Front. Clim. 3:638805. doi: 10.3389/fclim.2021.638805<br><br>Schenuit, F., Brutschin, E., Geden, O., Guo, F., Mohan, A., Oliveira Fiorini, A. C., … Riahi, K. (2025). Taking stock of carbon dioxide removal policy in emerging economies: developments in Brazil, China, and India. Climate Policy, 25(1), 89–108. https://doi.org/10.1080/14693062.2024.2353148<br><br>In addition, in the **methods section** we now clarify that "Our experimental design does not estimate the effects of national policy trajectories directly; rather, it tests whether selected implementation attributes are evaluated similarly or differently across countries whose CDR pathways, governance and incentives are nascent."

Reviewer 2

I appreciate the authors' efforts to revise the manuscript and to engage with earlier comments. The paper has improved in several respects. However, I still have some important concerns that I believe need to be addressed.	Thank you for the positive feedback and the additional comments. We have revised the manuscript accordingly.
First, I am not convinced that the highly skewed results for Brazil and Saudi Arabia should be relegated to the supplementary material. These findings raise substantive issues that are central to the interpretation of the study. At least two plausible explanations should be considered more explicitly in the main text: (1) that the skewness reflects data quality concerns, such as inattentive respondents or respondents providing socially desirable answers rather than their genuine views; or (2) that the results reflect meaningful contextual differences between these countries and the others in the sample. In either case, these patterns are sufficiently striking that most readers will notice them, and they therefore warrant fuller discussion in the main manuscript rather than in an appendix.	Thank you for pointing this out. We agree that the skewed distributions need to be addressed more explicitly and in the main text. Following your suggestion, we now discuss this in the results section describing figure 1. Here, we reflect in particular on the possibility of cross-cultural variation in response styles.<br><br>We agree that data-quality artefacts cannot be fully ruled out in cross-country online surveys. We therefore now treat the high-end skew in Brazil and Saudi Arabia as a limitation in interpreting absolute support levels. At the same time, our quality-control procedures, including attention checks, response-time monitoring, and removal of low-quality responses, reduce the likelihood that inattentiveness alone explains the pattern. As highlighted in the Methods, we included three different attention checks along the survey, one of which came directly after the vignette experiment, and excluded fast respondents (i.e. those who took less than 30% of the median time).<br><br>**In the main text, results section describing figure 1:**<br>"When interpreting these results, cross-cultural variation in response tendencies deserves careful consideration. The pronounced skew toward high support particularly for Brazil and Saudi Arabia may reflect contextual differences, cross-cultural response styles, social desirability, or other unobserved factors. While our design and quality-control procedures reduce concerns about data-quality artefacts, they cannot

	eliminate them. We therefore interpret cross-country differences in absolute support cautiously and emphasize the marginal effects of experimentally-varied vignette attributes."<br><br>We also expanded the acknowledgment of limitations in the "Data Analysis" section of the Methods. Despite the space constraints, we hope to have now addressed your concerns.<br><br>**In methods:**<br>"Several caveats of cross-country survey research are noteworthy. Response styles may differ across countries. For instance, Asian respondents are less likely to choose extreme response categories and more prone to acquiescence bias, i.e., agreeing with statements or responding affirmatively, compared to western respondents[93,94]. Caution is, thus, warranted when interpreting responses across contexts as they may partly reflect cultural differences in survey behaviour, rather than underlying preferences. This applies to the interpretation of our findings on overall support. Figure 1 shows higher average support for CDR technologies in Global South countries compared to Global North countries, consistent with previous studies[49]. Response style differences may partly explain this pattern, although our design does not allow us to fully separate response-style effects from substantive contextual differences. As we are aware of this issue, we reported level differences but focused on the interpretation of the differences and similarities in the marginal effects of the vignette attributes."

Second, while I appreciate that the authors now engage more with differences in regime type, I find the terminology somewhat imprecise. Referring to Saudi Arabia as a “more centralized governance” system risks obscuring important distinctions. In the comparative politics literature, Saudi Arabia is more accurately described as a ”closed autocracy” or ”absolute monarchy”.	Thank you for pointing this out. We agree that this formulation was too imprecise and now more accurately speak of “autocratic” political system instead (see Introduction). In the description of the country selection in the Supplementary Information we have also revised the relevant paragraph to describe the sample as spanning liberal democracies, constitutional monarchies, and autocratic regimes.
Third, I remain unconvinced by the argument that if respondents in Saudi Arabia lacked well-formed opinions about public consultations, we would expect null or only very modest effects in the relevant experimental dimension. This inference seems too strong. The presence of variation in responses does not, in itself, demonstrate that respondents interpret the survey items in the intended way. There remains a substantial risk that key concepts, such as public consultation, are not understood equivalently across contexts. This concern is particularly salient in cross-national research that includes both democratic and authoritarian settings. I therefore encourage the authors to acknowledge this limitation more explicitly and to discuss its implications for the interpretation of the findings.	Thank you for this clarification. We certainly agree that the lay understanding of “public consultation” might differ across diverse socio-political contexts. It is for this reason that we decided not to use such terminology in the vignettes and rather say that “the project is permitted by the authorities without asking the opinion of the affected local population…” or “...after listening to the opinion of the affected local population”. As we mentioned, our ambition with the vignettes was to elicit what respondents envisioned to be desirable scenarios, rather than assessing the current status. However, we recognize that “asking the opinion of the local population” could mean different things for people in different socio-political contexts (which the nuanced results in fig. 4 on the engagement item after the vignettes indeed suggest).<br><br>**In the results section** describing figure 2 we now added the following words of caution: “This should not be interpreted as evidence that consultation is understood identically across political contexts; it shows only that the experimentally specified contrast between no opinion-seeking and opinion-seeking increased support in Saudi Arabia as elsewhere.”<br><br>On top of this and the additions to the results section made in the first round of revisions, we now addressed your comments in the following two ways: (i) in the discussion

	section we reflect on the need for further research on what engagement means to publics in different political systems and (ii) in the methods section we explicitly acknowledge the possibility of varying interpretations as a limitation of the study.<br><br>**In the discussion:**<br>"What engagement – operationalised in our experiment as asking for and listening to the opinions of local populations - means to publics in diverse political systems, particularly in authoritarian contexts, requires further attention and qualitative, deliberative research."<br><br>**In methods:**<br>"Cross-country survey research, furthermore, carries the risk that key concepts may not be interpreted equivalently across contexts. When formulating the vignette attributes (see SI 12), we paid due attention to wording that would work across contexts. For example, instead of speaking of "public consultation", we described opening the decision process in the following way: "This project is permitted by the authorities without asking/after listening to the opinion of the affected local population". However, the caveat remains that respondents in democratic vs. authoritarian contexts might envision such processes differently. Future qualitative research is needed to elicit more nuanced, context-specific understandings and expectations regarding formats of public engagement, especially in relation to the emerging area of CDR governance."

Reviewer 4

Thank you for responding to our comments and concerns in a diligent and very constructive way. Through the revisions we think the manuscript has much improved, especially with regard to clarifying its novelty and strengthening the discussion of implications and limitations. The paper now makes an interesting contribution to the literature on governance architecture and public support for the deployment of CDR. In our view it is ready for publication.	Thank you very much for the positive feedback. We are happy to hear that and appreciate your constructive engagement with our manuscript!
